# A delayed translocation into the endoplasmic reticulum controls the post-translational modifications of PD-L1

**DOI:** 10.1038/s41467-026-71760-x

**Published:** 2026-04-11

**Authors:** Magda Cannata Serio, Fulvia Vitale, Gianluca Scerra, Raffaella Bonavita, Patrick Poullet, Maria Gabriella Caporaso, Laura Marrone, Simona Romano, Maurizio Renna, Franck Perez, Massimo D’Agostino

**Affiliations:** 1 Institut Curie, PSL Research University, CNRS UMR144 Paris, France; 2 Department of Molecular Medicine and Medical Biotechnology, University of Naples Federico II, Naples, Italy; 3 Institut Curie, Bioinformatics core facility (CUBIC), INSERM U1331, PSL Research University, Mines Paris Tech, Paris, France

**Keywords:** Protein translocation, Endoplasmic reticulum, Post-translational modifications

## Abstract

N-terminal signal peptides (SPs) are traditionally considered as drivers of co-translational translocation of newly synthesised proteins into the endoplasmic reticulum (ER). However, growing evidences suggest that proteins with SPs can also undergo post-translational insertion into the ER membrane after synthesis is complete. Recently, an intermediate third mechanism has been uncovered where proteins with marginally hydrophobic or suboptimal SPs are translocated following an initial delay after translation initiation. Here, we show that this “delayed translocation” allows a temporary exposure of the nascent chain to the cytosolic environment, enabling exoplasmic domain modifications by cytosolic enzymes. We report that programmed death ligand-1 (PD-L1) follows this pathway, featuring a suboptimal SP that exposes its extracellular domain to the cytosol, enabling AMPK-dependent regulation of PD-L1 function. Importantly, optimising the SP of PD-L1 eliminates the cytosolic exposure, disrupting PD-L1’s trafficking and maturation, highlighting the physiological importance of the delayed translocation mechanism.

## Abstract (web summary)

Here, the authors show that CytoRUSH reveals the weak nature of signal peptides, enabling pre-translocational modification of nascent protein chains. In PD-L1, this mechanism supports AMPK-dependent regulation of its expression.

## Introduction

Approximately one-third of the proteins expressed by eukaryotic cells are transported through the secretory pathway, which plays an essential role in maintaining various cellular functions, including metabolic regulation, signalling, adhesion or migration^1^. A portion of these proteins exhibits an amino-terminal signal peptide (SP) that efficiently guides them for translocation into the endoplasmic reticulum (ER). About 18% of human proteins possess an SP with over >3400 predicted distinct SPs^2^. Despite such a large diversity, SPs share a common structural framework: a central hydrophobic region (H-region) consisting of at least six hydrophobic amino acid residues, a cationic N-terminal domain (N-region) and a C-terminal segment (C-region) located between the H-region and the cleavage site on the N-terminus of the mature protein^3^. During translation, nascent chains emerging from ribosomes are recognised by the signal recognition particle (SRP), stalling protein synthesis. This facilitates the recruitment of the ribosome/nascent chain complex (RNC) to the Sec61 translocon complex, the entry site of the secretory pathway on the ER. Subsequently, the SRP releases the SP, allowing it to be inserted into the Sec61 complex. The translation then resumes as the nascent chain is guided and pushed through the Sec61 channel into the ER lumen. It is widely acknowledged that this process, known as co-translational translocation^4^, is essential to prevent premature protein folding before polypeptides enter the ER. In fact, only unstructured chains can be effectively translocated through the Sec61 channels^5^. The success of the co-translational process underscores the meticulous efficiency with which cells have evolved to ensure that the SRP swiftly recognises all SPs exposed at the N-termini of nascent chains emerging from ribosomes.

It has been shown that some proteins with an SP can be fully synthesised in the cytosol and translocated into the ER only at the end of the synthesis in a post-translational manner. This mechanism applies to small presecretory proteins, with fewer than 100 amino acids, as well as to certain large human presecretory proteins with short, apolar SPs. A less-studied third mechanism involves proteins with marginally hydrophobic or suboptimal SPs. While these proteins are efficiently recruited to the ER, their translocation is initially paused at the Sec61 translocon. This pause allows translation of a substantial portion of the protein, resulting in cytosolic exposure of a nascent-chain segment of approximately 200 amino acids^6–9^.

Thus, we hypothesise that despite bearing an N-terminal SP, nascent chains may undergo post-translational modifications (PTMs) in the cytosol before being translocated to the ER, and suboptimal SPs could enable such “delayed translocation” to provide a necessary time window for the acquisition of these modifications. Indeed, since the H-region of SPs is critical for both SRP recognition and protein translocation^10^, the presence of polar or charged residues within the H-region may reduce binding affinity and delay the initiation of translocation.

We report here that the immune checkpoint programmed death ligand-1 (PD-L1) is a member of this class of proteins that display delayed translocation into the ER. Together with its partner, programmed death-1 (PD-1), expressed on the surface of T cells, PD-L1 plays an essential role in dampening the immune response and preventing excessive immune activity and tissue damage. However, cancer cells can exploit the PD-L1/PD-1 pathway to evade immune control and continue to grow and spread^11^. In addition to transcriptional regulation, PD-L1 cell-surface overexpression can be modulated by various PTMs, including glycosylation, phosphorylation and ubiquitination, as well as by interactions with accessory proteins. Like many proteins, the extracellular domain of PD-L1 is modified during its transport by resident glycosylation enzymes in the Golgi Apparatus (GA), which significantly influences PD-L1 stability^12^. Recent research has reported that additional PTMs of PD-L1 luminal domain, specifically phosphorylation, are mediated by cytosolic kinases, like AMP-activated protein kinase (AMPK) at serine S195^13^, Janus kinase 1 (JAK1) at tyrosine Y112^14^, the never in mitosis gene A-related kinase 2 (NEK2) at tyrosine T194 and T210^15^ and β isoform of glycogen synthase kinase 3 (GSK3ß) at tyrosine T180 and serine S184^15^. These PTMs have profound functional consequences: AMPK phosphorylation earmarks PD-L1 for proteasome-mediated degradation^13^, GSK3ß phosphorylation induces UPS-mediated PD-L1 degradation by ß-TrCP^12^, while phosphorylation by JAK1 and NEK2 enhances PD-L1 glycosylation, stability and maturation^14,15^. Despite lacking a signal sequence for ER translocation, luminal localisation has been proposed for these kinases^12–16^. However, the mechanism and regulation of such cryptic translocation remain enigmatic. Here, we report that PD-L1 possesses a suboptimal SP, which delays its translocation into the ER, exposing its luminal domain to the cytosolic environment and to AMPK-mediated phosphorylation prior to ER translocation.

## Results

### CytoRUSH assay reveals the suboptimal nature of the PD-L1 SP

**Fig. 1:**
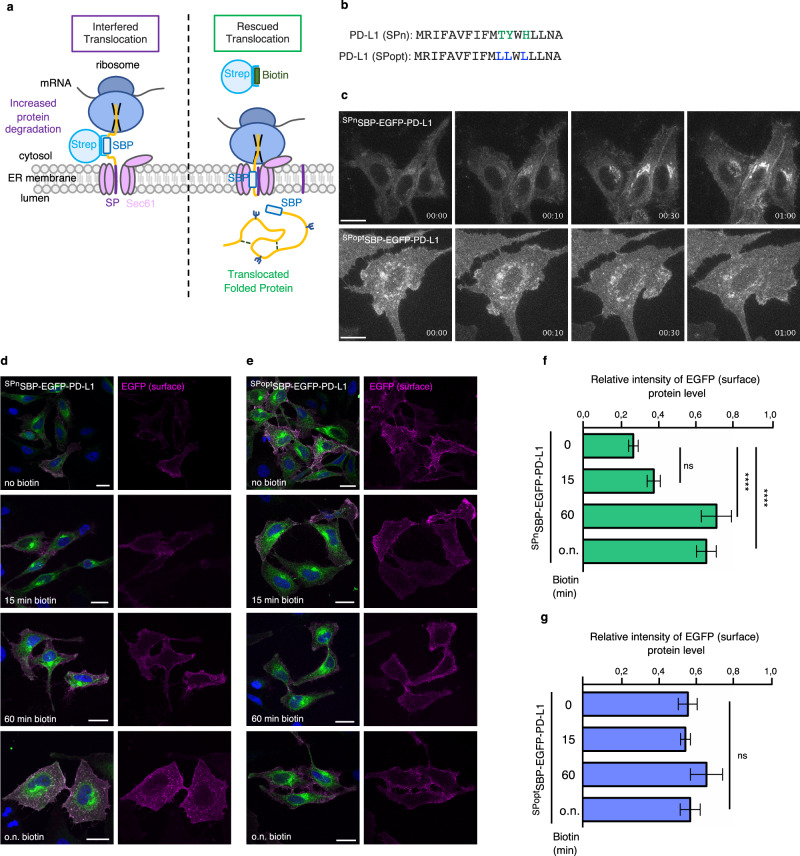
CytoRUSH reveals the suboptimal nature of the PD-L1 SP. **a** Schematic view of the cytosolic version of the cytoRUSH assay. **b** Schematic view of PD-L1 SP mutagenesis. Polar and positively charged amino acids within the SPn of PD-L1 are highlighted in green. Residues mutagenized in the SPopt of PD-L1 are highlighted in blue. **c** HeLa cells were transiently transfected with SPn- or SPopt-SBP-EGFP-PD-L1 expression vectors in combination with the cytosolic streptavidin in a ratio of 1:1 for 24 h. Biotin was added at 0 min (time scale shown as hr: min). Scale bar: 20 µm. **d**, **e** HeLa cells were transiently transfected with the cytosolic streptavidin and SPn-SBP-EGFP-PD-L1 or SPopt-SBP-EGFP-PD-L1 for 24 h. Some cells were treated overnight with 40 µM biotin (o.n. biotin). The following day, the other cells were treated with 40 µM biotin for the specified durations (15 or 60 min) before undergoing surface staining immunofluorescence. Cell surface expression of PD-L1 was analyzed by confocal microscopy using a specific antibody against the EGFP tag on non-permeabilized cells. Single focal sections are shown. Scale bar: 20 µm. **f**, **g** The histograms illustrate the relative intensity of the EGFP (surface) protein level. Statistical analysis was performed based on results obtained from *n* = 10 independent measurements per group from three independent experiments. All data are mean ± SEM; statistical significance was determined by ordinary one-way ANOVA followed by a Bonferroni multiple comparisons test. Levels of significance: **** *P* < 0.0001; ns, not significant. Source data are provided as a Source Data file.

Previous studies have shown that several cytosolic kinases modify the PD-L1 luminal domain^12–15^. Although it was proposed that this was driven by a cryptic presence of these various kinases in the ER, we hypothesised that, conversely, it may be due to an extended exposure of the PD-L1 luminal domain in the cytosol caused by a suboptimal SP. It is known that hydrophobic amino acids in the H-region of a SP, such as leucine^17^, play a major role in different steps of ER-translocation^18^. Intriguingly, the SP of PD-L1 from different species contains several polar or positively charged amino acids (highlighted in red) within the H-region (Fig. S1A). Kyte-Doolittle analysis (Fig. S1B, C) confirmed the lower average hydrophobicity degree of the H-regions of PD-L1 SPs from 91 different species (Table S1) when compared with the equivalent regions of SPs extracted from 100 randomly selected secreted proteins and 100 randomly selected transmembrane proteins (Table S2A, B). This suggests that a suboptimal SP has been selected and conserved during the evolution of PD-L1 and may thus play an essential physiological role. We challenged this hypothesis by optimising the PD-L1 SP (hereafter called SPopt) by replacing the uncommon polar and charged amino acids (T11, Y12 and H14) present in the human native SP (SPn) with leucine residues. Such substitutions are commonly employed to increase the hydrophobicity of SPs, thereby improving their efficiency^19^ (Fig. 1b).

To further dissect the role of these residues, we generated intermediate stepwise mutants (1 L, 2 L and 3L-SPopt) by progressively replacing T11, Y12 and H14 with leucines (Fig. S2A). 1 L and 2 L behaved similarly to the native SPn, displaying normal trafficking through the secretory pathway, whereas 3L-SPopt accumulated in the ER (Fig. S2B, C). Deglycosylation analysis confirmed these observations: SPn, 1 L and 2 L acquired complex glycosylation, while 3L-SPopt displayed a slightly lower apparent molecular weight in the untreated condition but converged to the same size as the other variants upon deglycosylation. This behaviour indicates that 3L-SPopt is only partially glycosylated and remains EndoH-sensitive, consistent with ER retention (Fig. S2D). Because only the full three-residue optimisation profoundly altered SP behaviour, and the intermediate variants were indistinguishable from SPn, we selected SPopt as the representative “efficient SP” for all subsequent analyses.

To analyse the impact of PD-L1 SPn suboptimality and determine whether SPn and SPopt undergo classical co-translational translocation, we developed a modified version of the RUSH assay. In this adaptation (which we called cytoRUSH), we expressed streptavidin in the cytosol and positioned the Streptavidin-Binding Peptide (SBP)^20^ on the luminal side of PD-L1, immediately after the cleavage site of the SP (^SP^SBP-EGFP-PD-L1). According to the co-translational translocation model, highly efficient SPs (such as SPopt) should prevent exposure of the luminal domain in the cytosol before translocation. Thus, the SBP should not be able to bind to cytosolic streptavidin even when positioned close to the SP. Conversely, we reasoned that a suboptimal SP, such as SPn, may delay translocation, exposing the SBP in the cytosol before translocation, thereby enabling capture by cytosolic streptavidin. Streptavidin binding to SBP may then prevent the translocation of PD-L1 into the ER, and this inhibition can be competed out by biotin (Fig. 1a).

Remarkably, the cytoRUSH assay revealed that the SPn of PD-L1, but not the SPopt, enabled biotin-sensitive capture of the PD-L1 luminal domain in the cytosol. This capture induced a pronounced decrease in PD-L1 protein levels, whereas PD-L1 under the SPopt remained unchanged under the same conditions (Fig. S3A, B). Biotin addition induced a rapid appearance of ^SPn^SBP-EGFP-PD-L1 [noted ^SPn^PD-L1 to indicate that the SP has been cleaved] in the GA and then at the cell surface, while it did not affect ^SPopt^SBP-EGFP-PD-L1 [^SPopt^PD-L1]. This suggested that ^SPopt^SBP-EGFP-PD-L1, but not ^SPn^SBP-EGFP-PD-L1, undergoes a classical co-translational translocation. Indeed, ^SPn^SBP-EGFP-PD-L1, but not ^SPopt^SBP-EGFP-PD-L1, had been captured in the cytosol by streptavidin, preventing its translocation into the ER and further transport toward the cell surface (Fig. 1c–g, Movies S1, S2). This capture induced partial degradation, likely via the proteasome, as indicated by MG132 sensitivity (Fig. S3C, D). As expected, biotin addition prevented the degradation of newly expressed ^SPn^SBP-EGFP-PD-L1, while it did not affect ^SPopt^SBP-EGFP-PD-L1 (Fig. S3A, B). To further validate the robustness of the cytoRUSH assay and to confirm its ability to discriminate between co-translational and delayed/post-translational translocation, we tested a panel of SPs identified through an extensive bioinformatic analysis of H-region hydrophobicity across all human SPs (Supplementary Data 1). Each selected SP was cloned upstream of SBP-EGFP-PD-L1 and expressed in HeLa cells in the presence or absence of overnight biotin treatment. CD44 and α-galactosidase A (AGAL), two SPs previously reported to undergo delayed translocation^6^ and characterised by low H-region hydrophobicity and the presence of aromatic amino acids, showed a significant biotin-dependent increase in surface PD-L1, similarly to ^SPn^SBP-EGFP-PD-L1 (Fig. S4A–C). In contrast, SPs with high hydrophobicity and enriched in leucine residues, properties associated with efficient Sec61 engagement^21^, displayed no differences between biotin-treated and untreated cells, consistent with the absence of cytosolic exposure and a fully co-translational mode of translocation (Fig. S4A–C). We next examined SPs from proteins known to undergo post-translational translocation. Pro-thyrotropin-releasing hormone (TRH)^22^ and statherin (STATH)^23^ exhibited a pronounced biotin-dependent rescue of PD-L1 surface expression, further validating the sensitivity of cytoRUSH in detecting post-translational translocation events (Fig. S4A–C). Finally, we tested the programmed cell death 1 ligand 2 (PD-L2), an SP with low hydrophobicity comparable to SPn but containing multiple leucine residues similar to SPopt. Despite its low hydrophobicity, PD-L2 behaved like SPopt and showed no biotin-dependent rescue, indicating efficient co-translational engagement of the translocon, likely driven by its leucine-rich H-region (Fig. S4A–C). Finally, the cytoRUSH assay demonstrated that when fused to a model ER protein (SP-SBP-EGFP-KDEL), SPn, but not SPopt, was able to drive a streptavidin-dependent and biotin-sensitive degradation of the reporter protein (Fig. S3E, F). Altogether, the above results revealed the role of suboptimal SP in enabling delayed translocation and cytosolic exposure of luminal domains.

### The suboptimal SP of PDL1 is essential to allow AMPK-dependent modification

**Fig. 2:**
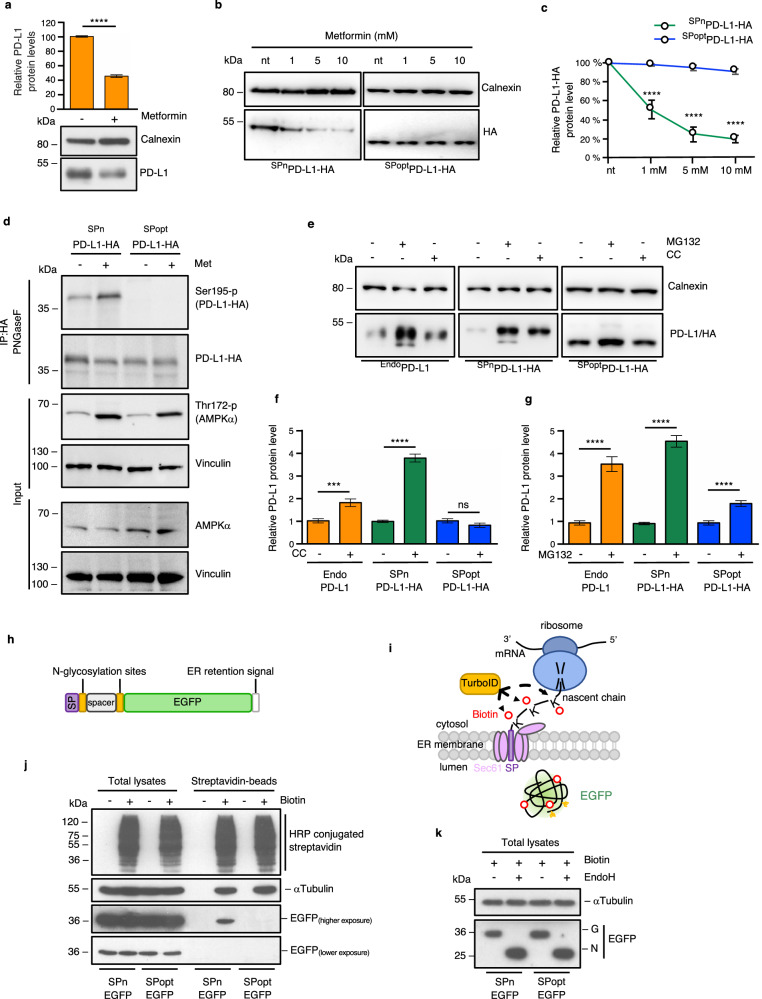
Effect of AMPK modulation activity on SP-variants of PD-L1. **a** Total cell extracts from GB138 cells, treated with or without Metformin (5 mM) for 24 h were analyzed by SDS-PAGE, and endogenous PD-L1 protein levels were analyzed by using a specific antibody against PD-L1. **b** GB138 cells stably expressing SPn- and SPopt-PD-L1-HA were treated with an increasing concentration of Metformin for 24 h, and PD-L1 protein levels were analyzed by SDS-PAGE. **c** Expression levels of PD-L1 relative to those of Calnexin were calculated. **d** HeLa cells stably expressing SPn- and SPopt-PD-L1-HA were treated with or without Metformin (5 mM) for 8 h. PD-L1-HA was then purified by IP and subjected to immunoblotting with an anti phosphoAMPK substrates multimab mix antibody after PNGase F reaction. **e** GB138 cells stably expressing SPn- and SPopt-PD-L1-HA or GB138 wild type cells were treated with 5 µM of the proteasome inhibitor (MG132) or 10 µM of the AMPK-antagonist compound C (CC) for 6 h before processing total cell extracts by SDS-PAGE. **f** Expression levels of PD-L1 treated or not with compound C relative to those of Calnexin were calculated. **g** Expression levels of PD-L1 treated or not with MG132 relative to those of Calnexin were calculated. **h** Schematic overview of plasmids used in the biotinylation experiment: N-glycosylation sites are indicated by yellow boxes, and the ER retention signal KDEL is represented by the white empty box at the C-terminus of the protein. **i** Proposed model illustrating the expected outcome of the biotinylation experiment in the presence of SPn: biotin molecules (indicated by red dots) are shown binding to the exposed Lysine (K) of the SPn-EGFP-KDEL sequence. **j** HeLa cells were transfected with V5-TurboID-NES and either SPn-EGFP-KDEL or SPopt-EGFP-KDEL at a 1:1 ratio. The transfected cells were treated with 150 μM biotin for 10 h. Biotinylated proteins were then pulled down using streptavidin-coated magnetic beads. Total lysates and the streptavidin-pulled-down proteins were analyzed by SDS-PAGE. ^SPn^EGFP-KDEL and ^SPopt^EGFP-KDEL were detected using an anti-EGFP antibody, while α-Tubulin was detected using a specific antibody. Biotinylated proteins were detected using an HRP-conjugated streptavidin. **k** ^SPn^EGFP-KDEL, ^SPopt^EGFP-KDEL and α-Tubulin proteins from the total lysates treated with biotin were subjected to EndoH digestion and analyzed by SDS-PAGE. ^SPn^EGFP-KDEL and ^SPopt^EGFP-KDEL were detected using an anti-EGFP antibody, while α-Tubulin was detected using a specific antibody. G Glycosylated PD-L1, N native PD-L1. All data are mean ± SD; statistical significance was determined by Student’s t-test with data from *n* = 3 independent experiments. Levels of significance: **** *P* value < 0.0001; *** *P* value < 0.001; ns, not significant. Source data are provided as a Source Data file.

Previous studies have shown that AMPK promotes PD-L1 protein destabilisation and proteasomal degradation through PTMs^13^ (Fig. S1D). Because its native SP exposes the luminal domain of PD-L1 to the cytosol, we explored the potential impact of increasing PD-L1's ER translocation rate on its sensitivity to AMPK activity. As shown in Fig. 2a, the level of endogenous PD-L1 was significantly reduced upon treatment with several AMPK activators, metformin^13,24^, A769662^25^ and AICAr^26,27^ (Fig. S5A). Dose-dependent sensitivity to metformin was also observed for recombinant ^SPn^PD-L1-HA ectopically expressed in glioblastoma GB138 cells. Strikingly, a variant of PD-L1 bearing the optimised SPopt (^SPopt^PD-L1-HA) was insensitive to either metformin (Fig. 2b, c) or more selective AMPK activators treatment (Fig. S5B) and indeed was not phosphorylated by AMPK (Fig. 2d). Similarly, treatment with a known less selective AMPK-antagonist, Compound C^26,28^ (Fig. 2e, f), or a more selective AMPK inhibitor, SBI-0206965^29^ (Fig. S5C), induced a significant increase of both the endogenous PD-L1 and exogenous ^SPn^PD-L1 protein levels, while no such increase was observed in the case of the ^SPopt^PD-L1. Notably, the non-phosphorylable (S195A) or pseudo-phosphorylated (S195E) PD-L1-HA mutants, bearing either SPn or SPopt, were totally resistant to all AMPK activators and inhibitors (Fig. S5A–C). Additionally, to discriminate the contribution of S195 phosphorylation from SP-dependent effects, we analysed the behaviour of the S195A and S195E PD-L1 mutants expressed under the control of either SPn or SPopt (Fig. S6A–C). ^SPn^S195A behaved similarly to ^SPn^PD-L1 in terms of trafficking and glycosylation, whereas ^SPn^S195E was fully retained in the ER, displayed altered glycosylation and remained EndoH/PNGaseF-sensitive. In contrast, both ^SPopt^S195A and ^SPopt^S195E accumulated in the ER and showed complete EndoH and PNGaseF sensitivity, comparable to ^SPopt^PD-L1 (Fig. S6, A–C). These results indicate that abolishing the delayed translocation mediated by SPn engages PD-L1 into a strictly co-translational pathway, preventing cytosolic access to S195 and suppressing phospho-dependent regulation.

Using MG132 to inhibit proteasome-mediated degradation, we showed that the observed PD-L1 instability was due to proteolysis (Fig. 2e, g). Thus, PD-L1 expression depends on proteasome-dependent degradation regulated by AMPK and is influenced by the nature of the SP: a suboptimal SP allows a less efficient ER translocation, which favours PD-L1 expression modulation by AMPK and proteasome-dependent degradation, while an optimised SP protects PD-L1 from such modulation.

### SPn-mediated exposure to the cytosol enables PTMs without impeding ER translocation

Our data thus suggested that AMPK got access to the luminal portion of ^SPn^PD-L1 but not ^SPopt^PD-L1. To confirm this was not specific to AMPK, we investigated whether a nascent chain, during its transient exposure to the cytosol, can undergo PTMs by cytosolic enzymes other than AMPK prior to translocation completion. To this aim, we transiently expressed in the cytosol V5-TurboID, a highly efficient promiscuous biotinylation enzyme^30^, together with a reporter protein *2N-glyc*-EGFP-KDEL bearing an N-terminal SPn- or SPopt. This reporter contains two N-glycosylation sites and the ER-retention signal (KDEL) and should be stably localised in the ER (Fig.  2h). This experiment questions whether, depending on the nature of the SP, proteins can be modified in the cytosol, even by a promiscuous enzyme like TurboID, before being translocated and glycosylated in the ER (Fig. 2i). A cytosolic protein, α−Tubulin, was efficiently biotinylated in both conditions and used as a positive control (Fig. 2j). Consistent with the co-translational translocation model, reporter proteins bearing an N-terminal SPopt were not biotinylated in the cytosol. In contrast, SPn-bearing proteins were efficiently modified by cytosolic V5-TurboID (Fig. 2j). EndoH digestion confirmed the presence of the N-glycans for either SPn- or SPopt-equipped version of the EGFP reporter proteins (Fig. 2k), indicating that both were translocated into the ER. The size of the biotinylated reporter protein pulled down by streptavidin beads indicated that the proteins were glycosylated. Therefore, SPn-*2N-glyc*-EGFP-KDEL proteins were translocated into the ER after cytosolic modification by TurboID, thereby confirming that a suboptimal SP, like SPn, but not an optimised one like SPopt, enables the modification of the nascent chain by cytosolic enzymes before translocation into the ER lumen.

### Translation rate influences PD-L1 expression modulation

**Fig. 3:**
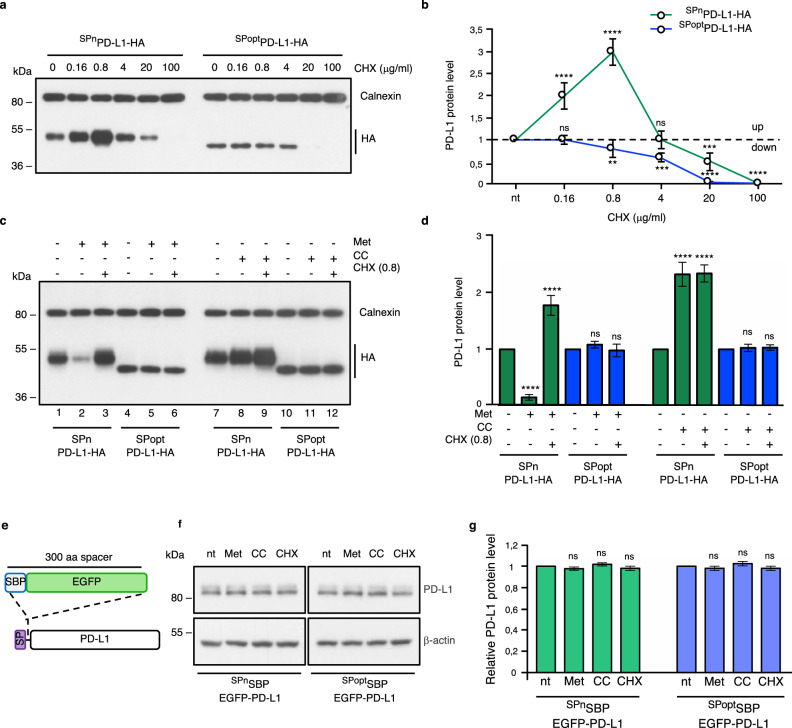
Effect of slowing translation on modulation of PD-L1 protein expression. **a** GB138 cells stably expressing SPn- and SPopt-PD-L1-HA were treated with increasing amounts of cycloheximide (CHX, from 0.16 to 100 μg/ml) for 8 h, and PD-L1 protein levels were analyzed by SDS-PAGE. **b** Expression levels of PD-L1 relative to those of Calnexin were calculated. **c** GB138 cells stably expressing SPn- and SPopt-PD-L1-HA were treated with 5 mM Metformin (Met) and 10 μM of compound C, either alone or in combination with 0.8 μg/ml of CHX, for 8 h. PD-L1 protein levels were analyzed by SDS-PAGE. **d** Expression levels of PD-L1 relative to those of Calnexin were calculated. **e** Schematic representation of SP-SBP-EGFP-PD-L1 expressing cassette. **f** GB138 cells were transiently transfected with SPn-SBP-EGFP-PD-L1 or SPopt-SBP-EGFP-PD-L1, and after 24 h treated with 5 mM Metformin (Met), 10 μM of compound C, or 0.8 μg/ml of CHX for 8 h. Then cells were lysed, and protein expression levels were analyzed by SDS-PAGE. PD-L1 proteins were revealed by using a specific antibody against the EGFP tag. **g** Expression levels of PD-L1 relative to those of β-actin were calculated. All data are mean ± SD; statistical significance was determined by Student’s t-test with data from *n* = 3 independent experiments. Levels of significance: **** *P* value < 0.0001; *** *P* value < 0.001; ** *P* < 0.01; ns, not significant. Source data are provided as a Source Data file.

The modification of nascent PD-L1 before its translocation indicates that PD-L1 is not subject to a conventional co-translational translocation into the ER. Because of the suboptimal nature of the PD-L1 SP, we tested whether it undergoes post-translational translocation or delayed translocation, imposing a temporal window between translation and translocation of the newly synthesised polypeptide into the ER. SPopt would alleviate this lagging time, protecting PD-L1 from metformin-induced degradation. We thus investigated whether slowing translation could similarly reduce or even eliminate this delay, rendering SPn-bearing proteins resistant to metformin. We used low doses of the protein synthesis inhibitor cycloheximide (CHX) to slow down protein synthesis rather than fully blocking it^14,31^. As expected, when using high doses of CHX (between 20 and 100 μg/mL), we observed that the levels of both PD-L1 variants were significantly reduced (Fig. 3a, b). Strikingly, at lower doses of CHX (between 0.16 and 0.8 µg/mL), we observed a significant increase in the protein levels of ^SPn^PD-L1, while ^SPopt^PD-L1 remained unaffected (Fig. 3a, b). At this lower translation rate (0.8 μg/mL of CHX), ^SPn^PD-L1 became insensitive to metformin (Fig. 3c, d). Accordingly, CC did not show any synergistic effect with CHX treatment, resulting in the same increase in ^SPn^PD-L1 protein levels, whether treated alone or in combination (Fig. 3c, d). These data indicate that reducing translation speed prevents transient exposure of the luminal domain to the cytosol, thereby preventing phosphorylation of S195 by AMPK and protecting it from proteasomal degradation. Using the cytoRUSH, we confirmed this model by observing that a low dose of CHX (0.8 μg/mL) resulted in a moderate yet significant increase in ^SPn^PD-L1 protein levels in the absence of biotin (Fig. S7A–C).

This suggested that metformin’s effect on PD-L1 depends on a transient presence of the luminal domain in the cytosol due to a delayed recognition, or docking, of a suboptimal SP and not to a fully post-translational translocation. To confirm this hypothesis, we assessed whether sensitivity to AMPK depends on the distance separating the AMPK-phosphorylated S195 from the SP. We inserted a long spacer (about 300 amino acids coding for the streptavidin-binding peptide (SBP) and EGFP) between the SPn and the beginning of the PD-L1 nascent chain (Fig. 3e) and measured the effects of metformin and CC (Fig. 3f, g). The introduction of the spacer effectively rendered PD-L1 resistant to all treatments, likely preventing its exposure and phosphorylation at S195 in the cytosol and proteasomal degradation (Fig. 3f, g). These results suggest that the presence of a suboptimal SP (SPn) induces a delay in completing the translocation of PD-L1 into the ER. This provides enough time to allow the nascent chain to be exposed to the cytosolic environment, which is required for AMPK-dependent down-modulation of PD-L1. The presence of the more efficient SPopt, or a slower translation rate, would prevent S195 exposure in the cytosol and its modification by AMPK before translocation. Altogether, our findings indicate that the AMPK-dependent down-modulation of PD-L1 is not due to a cryptic translocation of the kinase into the ER but rather to the presence of the suboptimal SPn, which determines the appropriate temporal window between translation and translocation to the ER.

### SP influences PD-L1 glycosylation and trafficking through the secretory pathway

Our analysis done using two different prediction tools (SignalP 6.0, which is able to predict the presence of SPs and the location of their cleavage sites in proteins, and Phobius, which predicts transmembrane topology and SPs in proteins) suggested that the optimisation of the SP of PD-L1 is unlikely to affect SP cleavage (see for example Fig. S8A, B)^32,33^. Moreover, we observed comparable secretion kinetics of EGFP reporter proteins equipped with both versions of PD-L1 SPs, (Fig. S8C), while defects in SP hydrolysis would strongly affect ER export and trafficking and prevent release in the medium. To further confirm this, we mutated Ala18 into Tyr^34^, which was predicted to abolish SP cleavage by both SignalP 6.0 and Phobius (Fig. S9A, B). Accordingly, when expressed in HeLa cells, ^SPn^A18Y-PD-L1 and ^SPopt^A18Y-PD-L1 were not able to reach the cell surface, as shown by endpoint (Fig. S9C, D) and dynamic studies using the RUSH assay^35^ (Fig. S9E–G, Movies S3-4), supporting the idea that SPopt did not lead to SP cleavage defects.

**Fig. 4:**
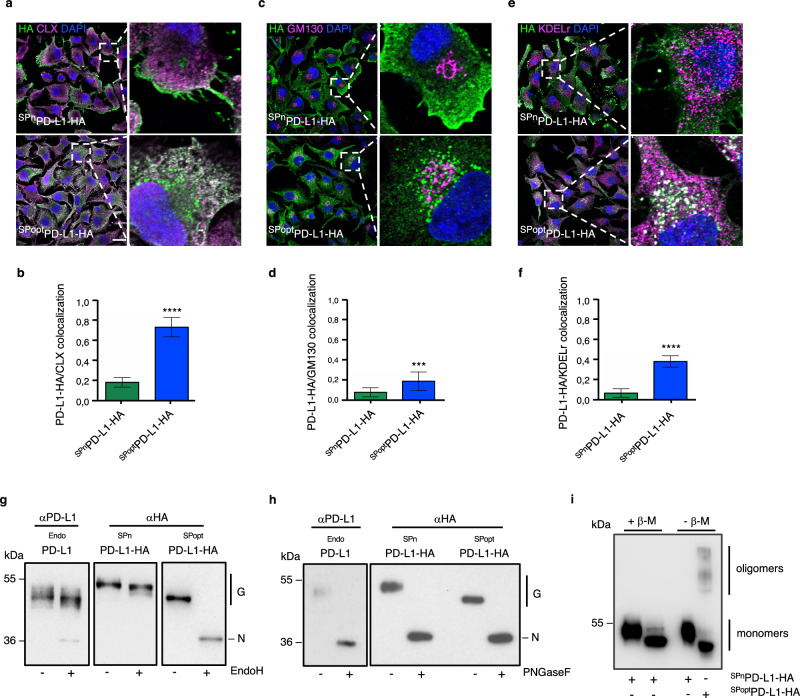
Effect of SP optimisation on PD-L1 glycosylation, maturation and cellular distribution. **a**, **c**, **e** GB138 stably expressing SPn- and SPopt-PD-L1-HA were seeded on coverslips and fixed before being processed by indirect immunofluorescence with a specific antibody against HA tag and different intracellular markers calnexin (CLX, marker of the ER), GM130 (a marker of the cis-Golgi), and KDELr (a marker of the Golgi-ER retrograde transport). Magnifications are shown in dashed white squares on the right of each panel. Single focal sections are shown. Scale bar: 20 μm. **b**, **d**, **f** The histograms illustrate the colocalization, expressed as Pearson’s coefficient, between PD-L1-HA variants and the above-mentioned intracellular markers. 50 randomly selected cells from each experiment were measured for co-localisation. Mean values were obtained from three independent experiments. **g** Total cell extracts of GB138 cells, as well as SPn- and SPopt-PD-L1-HA stably expressing GB138 cells, were separated on SDS-PAGE before and after EndoH digestion. Detection was performed using specific antibodies against the HA tag or the endogenous PD-L1 protein. G: Glycosylated PD-L1, N: native PD-L1. **h** Total cell extracts of GB138 cells, as well as SPn- and SPopt-PD-L1-HA stably expressing GB138 cells, were separated on SDS-PAGE before and after PNGaseF digestion. Detection was performed using specific antibodies against the HA tag or the endogenous PD-L1 protein. G: Glycosylated PD-L1, N: native PD-L1. **i** Total cell extracts of GB138 cells, as well as SPn- and SPopt-PD-L1-HA stably expressing GB138 cells, were separated on SDS-PAGE in the presence (+ ß-M) or absence of ß-merkaptoethanol (- ß-M). All data are mean ± SD; statistical significance was determined by Student’s t-test with data from *n* = 3 independent experiments. Levels of significance: Levels of significance: **** *P* < 0.0001; *** *P* < 0.001; ns, not significant. Source data are provided as a Source Data file.

Intriguingly, as shown in Fig. 2b, e, ^SPopt^SBP-EGFP-PD-L1 displayed a different molecular weight compared to ^SPn^SBP-EGFP-PD-L1, which may be due to trafficking defects or alteration in glycosylation. We first investigated whether the difference in the molecular weight of PD-L1 proteins translocated into the ER under the control of SPn and SPopt may be due to differences in trafficking through the secretory pathway. In cells stably expressing either ^SPn^PD-L1-HA or ^SPopt^PD-L1-HA, ^SPn^PDL1-HA was primarily located at the plasma membrane (Fig. 4a, c and e, Fig. S10A and B), whereas ^SPopt^PDL1-HA predominantly resided in the ER, as confirmed by its pronounced co-localisation with Calnexin (CLX), an ER resident transmembrane protein (Fig. 4a, b, Fig. S10A-C). To further characterise the trafficking behaviour of the two variants, we assessed co-localisation with TANGO1, a marker of ER exit sites (ERES) (Fig. S10A, B and D) and ERGIC-53, a marker of the ER-Golgi Intermediate Compartment (ERGIC) (Fig. S10A, B and F). ^SPn^PD-L1-HA showed minimal overlap with both markers and was mainly detected at the plasma membrane, consistent with efficient ER export and trafficking (Fig. S10A, D and F). In contrast, ^SPopt^PD-L1-HA exhibited markedly increased co-localisation with both TANGO1 and ERGIC-53, indicating an accumulation in the early secretory pathway (Fig. S10B, D and F). Co-localisation with GM130, a cis-Golgi marker, was also observed, suggesting that a small fraction of ^SPopt^PD-L1-HA can progress beyond the ER (Fig. 4 a, b and d, Fig. S10A, B and E). Moreover, we observed a significant increase in co-localisation with KDEL receptor (KDELr) (Fig. 4e, f, Fig. S10A, B and G), a marker of the retrieval pathway from the GA to the ER. To further investigate the observed trafficking defects of ^SPopt^PDL1, we employed a conventional RUSH assay^35^. Upon the addition of biotin, ^SPopt^SBP-EGFP-PDL1 displayed delayed trafficking kinetics compared to the ^SPn^SBP-EGFP-PDL1 (Fig. S11A–C, Movie S5-6), suggesting a physiological relevance of the suboptimal SPn in the efficacy of PD-L1 trafficking.

Lastly, we investigated the glycosylation state of both PD-L1 variants by treating protein lysates with different deglycosylation enzymes. Recombinant ^SPn^PD-L1 as well as endogenous PD-L1 were found to be sensitive to treatment with peptide-N-glycosidase F (PNGase F) but resistant to N-glycosidases endoglycosidase H (Endo H), indicating proper transport to the GA. In contrast, ^SPopt^PD-L1 glycosylation was efficiently cleaved by both enzymes, confirming that transport to the GA was strongly altered (Fig. 4g, h, Fig. S10A). Note that the sizes of ^SPn^PDL1 and ^SPopt^PDL1 were found to be identical after deglycosylation by PNGase F, further confirming that the size difference observed is due to glycosylation defects and not to partial proteolysis or improper SP cleavage. Finally, western blotting analysis under non-reducing conditions revealed the propensity of the ^SPopt^PD-L1-HA to form high-molecular-weight oligomers (Fig. 4i), indicating a folding defect of the mutant. These data suggest that optimising the SP impairs the folding and transport of PD-L1 and that the presence of suboptimal SP is essential to ensure efficient transport and maturation of PD-L1 along the secretory pathway.

## Discussion

**Fig. 5:**
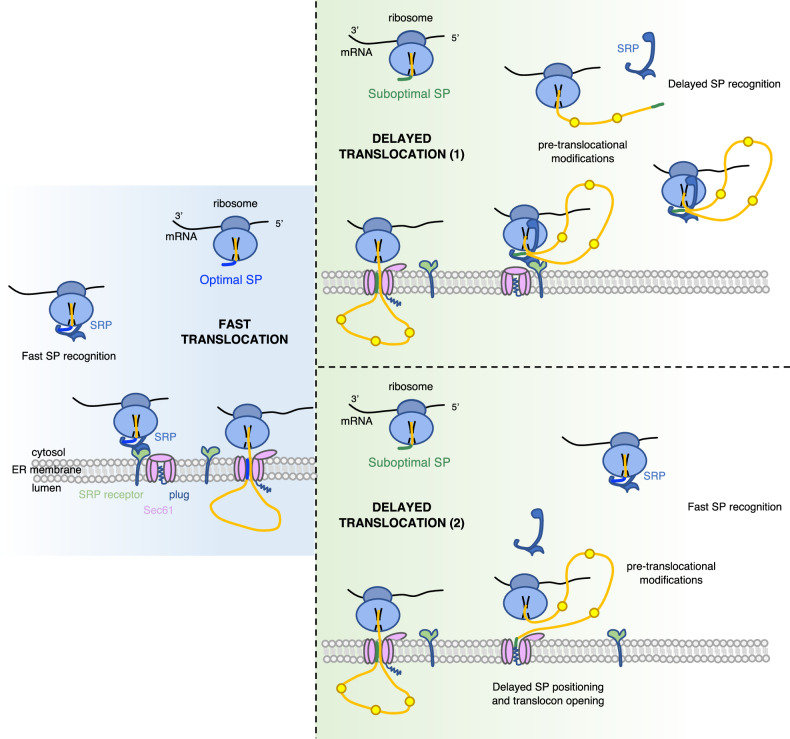
Different kinetics and potential for PTMs associated with optimal and suboptimal SPs during the translocation process. In this model, we illustrate the distinction between optimal (fast translocation, left part of the drawing) and suboptimal (delayed translocation (1) and (2), right part of the drawing) SPs during protein synthesis. For nascent chains with an optimal SP, translation initiates and the SRP binds to the nascent chain, pausing translation. This allows the ribosome-mRNA-nascent chain complex to redistribute on the cytosolic face of the ER by interacting with the SRP receptor. Subsequently, the SP inserts into the Sec61 channel, opening the lateral gate and displacing the plug domain, facilitating co-translational translocation. On the other hand, nascent chains with a suboptimal SP can undergo delayed translocation. This delay can be attributed to two different mechanisms: either a slowed recognition of the SP by the SRP, as depicted in “delayed translocation (1)” (right upper panel), or a delayed positioning of the SP and subsequent opening of the translocon, as illustrated in “delayed translocation (2)” (right lower panel). This delayed translocation permits the synthesis and exposure of a longer nascent chain to cytosolic enzymes capable of performing pre-translocation modifications (indicated by the yellow dots) prior to the initiation of translocation. Once the modified nascent chain is recruited to the ER membrane (delayed translocation (1)) or the SP opens the translocon (delayed translocation (2)), it is translocated into the ER lumen.

PD-L1 is a major player in immune control and requires fine-tuning of its activity in vivo. Among the various PTMs controlling its functions, it has been observed that PD-L1 extracellular domain is modified by several cytosolic kinases, such as AMPK, JAK1, NEK2 and GSK3β. To explain such a topology challenge, it was proposed that a fraction of these cytosolic kinases is translocated into the lumen of the ER, despite the absence of SP^12–16^, following a yet unidentified mechanism. On the other hand, recent evidence indicates that a substantial number of phosphorylated amino acids in the human proteome are cryptic, residing within the inner core of proteins’ native states and not exposed to solvent, suggesting that their modification occurs during the folding pathway^36^. Interestingly, the AMPK-targeted Serine 195 of PD-L1 seemed to fall into this category. Here, we report that PD-L1 SP drives a delayed ER translocation, allowing a transient exposure of the nascent chain to the cytosol. This discovery opens an alternative possibility for the PD-L1 protein’s expression control through the acquisition of co-translational modifications (CTMs) before its translocation into the ER. Our biotinylation assay demonstrates that such a transient exposure is sufficient to allow modification of a reporter protein bearing the SP of PD-L1 (SPn) before its translocation into the ER is completed. Importantly, optimising the SP (SPopt) abolished the acquisition of such a modification (Fig. 2). Therefore, it becomes a reasonable hypothesis that the PD-L1 luminal domain modifications depend on the transient exposure of its nascent chain to the cytosol before effective translocation (Fig. 5). Several lines of evidence support the fact that metformin-sensitive phosphorylation of the luminal S195 by AMPK depends on the delayed ER translocation driven by the suboptimal SPn and not on cryptic translocation of the kinase: (1) optimising PD-L1 SP; (2) increasing the distance between SPn and S195; (3) slowing down translation using low doses of CHX, all these measures alleviate metformin sensitivity. Through the development of a cytoRUSH assay, we also showed that SPn, but not SPopt, enables efficient interaction of a portion of the luminal domain with cytosolic factors. Altogether, our data indicate that, in addition to the classical co-translational and post-translational translocation models, some proteins can be translocated into the ER via a delayed translocation mechanism imposed by suboptimal SPs, providing a time window for the acquisition of pre-translational modifications.

Co-translational translocation involves an elongation delay induced by SRP after the emergence of the SP, allowing sufficient time for attachment to the Sec61 translocon and preventing folding of the chain before translocation^37,38^. Interestingly, studies have shown that many RNCs, isolated from yeast, encoding for secretory proteins, remain soluble for hundreds of residues even after the SP exposure^39^. Our data suggest an alternative model in which less hydrophobic or weak SPs may not be recognised as efficiently by SRP, leading to an extended delay in its elongation pausing activity. Alternatively, these less hydrophobic SPs might encounter challenges in engaging with the Sec61 translocon. In either scenario, the nascent chains could be transiently exposed to the cytosolic environment, allowing cytosolic enzymes to access them for modifications prior to translocation into the ER. In this framework, a more hydrophobic SP such as SPopt would alleviate this lagging time by promoting faster and more efficient engagement with SRP and the Sec61 translocon, thereby reducing or eliminating the window during which the emerging luminal region can be exposed to the cytosol. Conversely, decreasing the translation rate extends the time available for the suboptimal SPn to productively engage Sec61 before the S195-containing region emerges from the ribosome, preventing its transient cytosolic exposure.

Consistent with this interpretation, we find that reducing the translation rate diminishes the cytosolic exposure of the PD-L1 mature domain, indicating that a longer time window increases the probability of productive engagement of the suboptimal SP with the Sec61 translocon before the luminal region emerges from the ribosome. However, our data cannot resolve whether the native SP is held in a stalled conformation at the Sec61 translocon or whether it undergoes several low-efficiency insertion attempts before successful translocation. In addition, the translocation process through the Sec61 translocon involves multiple stages of activation, and the presence of polar residues in an SP could not only delay ER-membrane targeting through SRP, but may also result in slower translocation kinetics, as the SP passes through the translocon and in a proper insertion of the SP into the ER membrane. In recent years, it has been shown that precursor proteins with slowly gating SPs rely on allosteric effectors, such as TRAP or the Sec62/Sec63/BiP complex, to facilitate the opening of the Sec61 channel^22,40–45^. The hydrophobicity of SPs plays a crucial role in the dwell time at the cytosolic funnel of the Sec61 channel. If the interactions between the SP and the hydrophobic patch are too strong or not strong enough, it can hinder the spontaneous opening of the lateral gate and complete channel opening^43,44,46^.

Accessory proteins may also impose a lag in the translocation of polypeptides. Among the ribosome-bound proteins, the nascent polypeptide-associated complex (NAC) has been shown to act in the ER-targeting quality control by counteracting SRP binding to the signal-less RNCs and impeding those ribosomes from binding to the Sec61 translocon^47^. In this context, weak or suboptimal SPs could transiently delay SRP recruitment to the ribosome, thereby allowing NAC to keep RNCs far from the Sec61 translocon. Since NAC lacks the pausing-translation effect of SRP, PD-L1 nascent chain could then be cytosolic and prone to PTMs. Although less hydrophobic, the PD-L1 SP still needs to be bound and protected to remain soluble in the hydrophilic cytosolic environment, and NAC lacks this capability. However, the SRP-dependent protein translocation is conserved in prokaryotes, where a ribosome-associated protein, named trigger factor, has been discovered whose function is to compete with SRP for binding to the ribosome L23 subunit^48^, thereby retarding protein secretion by competing for SP recognition^49^. The trigger factor recognises a distinct class of SPs, characterised by an H-region rich in aromatic residues and flanked by positively charged residues^50^. Intriguingly, the H-region of the PD-L1 SP contains four aromatic residues (F4, F7, F9 and W13) flanked by two positively charged residues (R2 and H14) (Fig. S1A). A still elusive homolog of the prokaryotic trigger factor may thus play a crucial role in eukaryotes as well, by delaying translocation and allowing modification of cryptic phosphorylation sites required for the stability, folding and function of mature proteins.

Delayed translocation might play a key functional role, subjecting extracellular domains of proteins to both cytosolic and luminal modifications. We showed here that optimising the PD-L1 SP led to a variant defective for glycosylation, folding, maturation, trafficking and cellular localisation. This suggests that delayed translocation is essential to control PD-L1 function and may explain why a suboptimal SP has been conserved throughout evolution. It may be essential in the context of PD-L1 functions as rapid modulation of its expression is crucial for maintaining organismal homoeostasis. Indeed, PD-L1 expression on the cell surface not only enables cancer cells to evade immune surveillance but also physiologically controls and prevents prolonged activation of the immune system^51^. A delayed translocation driven by its suboptimal SPn allows the integration of the modulation of PD-L1 functions in cytosolic signaling circuits controlled, for example, by the AMPK, JAK1, NEK2 and GSK3β kinases. In line with these observations, our findings emphasize that the use of native, non-optimized SPs in recombinant constructs is important to faithfully reproduce the natural maturation, trafficking and regulatory properties of the corresponding proteins and that the use of optimized SPs in recombinantly produced proteins may lead to the loss of some of these features. The pronounced trafficking defect observed in the SPopt variant further suggests that abolishing the transient cytosolic exposure of the nascent luminal domain may prevent the acquisition of additional early PTMs or chaperone interactions required for proper folding and ER export. Although our data do not allow us to pinpoint the specific event, the loss of this pre-translocational window likely uncouples PD-L1 from regulatory inputs essential for its maturation and forward trafficking.

Finally, the suboptimal nature of certain SPs raises intriguing possibilities for therapeutic targeting. Indeed, recent findings have shown that small molecules or cyclic small peptides can interfere with the translocation of specific nascent chains, such as CD4 and VCAM1, resulting in the proteasomal degradation of the mature protein^52^. Moreover, recent studies have demonstrated that small-molecule inhibitors of the Sec61 translocon can efficiently reduce PD-L1 expression, further supporting the feasibility of targeting its translocation step^53^. However, given the peculiar features of the PD-L1 SP, additional studies will be essential to determine whether selective and reversible modulation of PD-L1 expression can be achieved through SP-directed strategies.

## Methods

### Reagents

Metformin (ab120847, Abcam) was added at the indicated concentrations and left on the cells for 24 h.

Compound C (171260; Sigma-Aldrich) was added at 10 μM and incubated with the cells for 8  h.

SBI-0206965 (AMPK inhibitor), AICAr and A769662 (AMPK activators) were purchased from SelleckChemicals and used at the indicated concentrations (0 to 1 µM) for 16 h.

MG132 (474790; Sigma-Aldrich) was added at 5 μM and incubated with the cells for 6  h.

Cycloheximide (01810; Sigma-Aldrich) was added at indicated concentrations (0.16 to 100 μg/mL and left on the cells for 8 h.

D-biotin (ref B4501; Sigma-Aldrich) was prepared and used as previously described^35^.

### Cell culture and transfection experiments

GB138 cells, established in 2011 from a resected adult GBM sample, were kindly provided by Bernard Rogister^54^. They were routinely grown at 37 °C in Dulbecco’s modified essential medium (DMEM; Sigma-Aldrich), containing 10% foetal bovine serum (FCS; Sigma-Aldrich S), 100 U/mL Penicillin/Streptomycin, 2 mM l-Glutamine (l-Gln; Sigma-Aldrich).

GB138 cells stably expressing the SPn- and SPoptPD-L1-HA protein were grown in the same medium, supplemented with 400 μg/ml G418 (Gibco).

HeLa cells were cultured in Dulbecco’s modified Eagle’s medium (DMEM; Thermo Fisher Scientific) supplemented with 10% foetal bovine serum (FCS; GE Healthcare), 1 mM sodium pyruvate, and penicillin and streptomycin (100 μg/mL) (Thermo Fisher Scientific). HeLa cells stably expressing the SPn-, 1L-, 2L-, 3L- SPopt-PD-L1-HA, PD-L1(S195A) and PD-L1(S195E) proteins were obtained by transduction with lentiviral particles produced in human embryonic kidney 293 T^55^. A clonal population was then selected using neomycin resistance and limiting dilution. The cells were then grown in the same medium, supplemented with 400 μg/m G418 (Gibco).

GB138 cells were always transfected by using X-tremeGENE HP DNA transfection reagent (Merck) according to the manufacturer’s instructions.

For RUSH live-imaging and cytoRUSH immunofluorescence experiments, HeLa cells were transfected using calcium phosphate as described previously^56^.

### Antibodies

The following antibodies were used: peroxidase-conjugated anti-mouse and anti-rabbit IgG (GTxMu-003-DHRPX; GTxRb-003-DHRPX; ImmunoReagents); Texas-Red-conjugated anti-mouse (AB_2338756) and anti-rabbit (AB_2338022) IgG, FITC-conjugated goat anti-mouse (AB_2238589) and anti-rabbit (AB_2337972) IgG, (Jackson ImmunoResearch Laboratories); mouse monoclonal (H9658) and rabbit polyclonal (H6908) anti-HA antibody (Sigma-Aldrich); rabbit polyclonal anti-CD274/PD-L1 antibody (NBP2-15791; Novus Biological); rabbit polyclonal anti-EGFP antibody (SC-9996; SantaCruz); rabbit polyclonal anti-Calnexin antibody (C4731; Sigma-Aldrich); anti β-actin (TA310155; OriGene); anti-GM130 (12480; Cell Signalling); anti-KDELr (10C3; Enzo); Streptavidin-HRP (21130; Pierce); anti-αTubulin (T5168; Sigma-Aldrich); rabbit polyclonal anti-streptavidin (S6390; Sigma-Aldrich); rabbit polyclonal anti-Calreticulin antibody (ab2907; Abcam); rat monoclonal anti-HA antibody (11867423001; Roche); rabbit polyclonal anti-Calnexin (2679; Cell Signalling Technology); mouse monoclonal anti-GFP (11814460001; Roche); multiMab® rabbit monoclonal antibodies mix anti-phospho-AMPK Substrate Motif [LXRXX(pS/pT) (5759; Cell Signalling Technology); rabbit monoclonal anti-AMPKα (5831; Cell Signalling Technology); rabbit monoclonal anti-AMPKα Thr172-p (2535; Cell Signalling Technology); mouse monoclonal anti-Vinculin (V9264; Sigma Aldrich); rabbit polyclonal anti-RTN4 (10950-1-AP; Proteintech); rabbit polyclonal anti-giantin (clone TA10, recombinant antibody facility of Institut Curie, Paris, France); rabbit polyclonal anti-TANGO1 (HPA055922; Sigma-Aldrich); mouse monoclonal anti-ERGIC-53 (sc-365158; Santa Cruz). Alexa Fluor 488 AffiniPure Donkey Anti-Mouse IgG (H + L) (715-545-151; Jackson ImmunoResearch); Cy3 AffiniPure Donkey Anti-Rabbit IgG (H + L) (711-165-152; Jackson ImmunoResearch). For dilutions, see Supplementary Data 4.

### Constructs, cDNA cloning and plasmid construction

PD-L1-turboGFP^57^ (Origene) was used as template for generating PD-L1-HA constructs. SPn-PD-L1-HA was generated by PCR using the following couple of oligos: Fw (EcoRI): 5’-CGGAATTCCCACCATGAGGATATTTGCTGTCTTTATATTC-3’; Rev (XbaI): 5’-GCTCTAGATTAAGCGTAATCTGGAACATCGTATGGGTATCCTCCTCCCCGTCTCCTCCAAATGTGTATCACTTTGC-3’. SPopt-PD-L1-HA was generated by PCR using the following couple of oligos: Fw (EcoRI): 5’-CGGAATTCATGAGGATATTTGCTGTCTTTATATTCATGACCCTGTGGCTGTTGCTGAACGCATTTACTGTCACGGTTCCCAAGG-3’; Rev (XbaI): 5’-GCTCTAGATTAAGCGTAATCTGGAACATCGTATGGGTATCCTCCTCCCCGTCTCCTCCAAATGTGTATCACTTTGC-3’. SP and HA tags were included in the oligo sequences.

pCI-NEO vector plasmids used for transient expression of SPn-SBP-EGFP-KDEL, SPopt-SBP-EGFP-KDEL, SPn-2N-glyc-EGFP-KDEL, SPopt-2N-glyc-EGFP-KDEL, SPn-EGFP, and SPopt-EGFP were generated by GenScript Biotech Corporation.

The plasmid FP2072 encoding for cytosolic streptavidin has been described before^58^. The plasmid FP4946 and FP4947 encoding for SPn- and SPopt-PD-L1-SBP-EGFP-PD-L1 were generated as follows: the SPn, the SPopt and PD-L1 sequences were generated from synthetic genes (IDT Integrated DNA Technologies) and cloned into a RUSH plasmid using the NheI and SbfI restriction sites (SP) or and FseI and PacI restriction sites (PD-L1). Both SPn and PD-L1 correspond to the native DNA sequences extracted from NCBI. For the plasmid FP4909 and FP4910 encoding for Strep-KDEL-SPn- and SPopt-SBP-EGFP-PD-L1, the PD-L1 sequence was extracted from SPn- and SPopt-SBP-EGFP-PD-L1with FseI and PacI restriction sites and inserted into Strep-KDEL_SPn- and SPopt-SBP-EGFP-GPI. For the plasmid FP5053 and FP5054 encoding for Strep-KDEL_SPnA18Y- and SPoptA18Y-SBP-EGFP-PD-L1, the sequences corresponding to SPnA18Y and the SPoptA18Y were generated from synthetic genes (IDT Integrated DNA Technologies) and cloned into the FP4909 and FP4910 plasmids using the AscI and SbfI restriction sites. For the plasmid FP5436 and FP5437 encoding for SPn- and SPopt-PD-L1-HA, the SPn-PD-L1-HA and the SPopt-PD-L1-HA sequences were generated from synthetic genes (IDT Integrated DNA Technologies) and cloned into a RUSH plasmid using the NheI and PacI restriction sites. For the plasmid FP5438 and FP5439 encoding for SPnA18Y- and SPoptA18Y-PD-L1-HA, the sequences corresponding to SPnA18Y and SPoptA18Y were extracted from FP5053 and FP5054 with NheI-EcoRI restriction sites and inserted into FP5437 and FP5438. The plasmids FP5605 and FP5606 encoding for pCDH1_SPn-PD-L1-HA and pCDH1_SPopt-PD-L1-HA, used to transduce HeLa cells, were obtained by cloning SPn-PD-L1-HA and the SPopt-PD-L1-HA sequences from FP5436 and FP5437 into the pCDH1 vector using the NheI and PacI restriction sites. V5-TurboID-NES_pCDNA3 was a gift from Alice Ting (Addgene plasmid #107169).

For the plasmid FP5950 and FP5951 encoding for SPn- and SPopt-PD-L1-HA, the SPn-PD-L1-HA and the SPopt-PD-L1-HA sequences were generated from synthetic genes (IDT Integrated DNA Technologies) and cloned into a lentiviral plasmid using the NheI and PacI restriction sites. For the plasmid FP5952 and FP5953 encoding 1L- and 2L-PD-L1-HA, the 1L-PD-L1 and the 2L-PD-L1 sequences were generated from synthetic genes (IDT Integrated DNA Technologies) and cloned into the FP5950 plasmid using the NheI and BamHI restriction sites.

For the plasmids FP5954, FP5955, FP5956, and FP5957 encoding SPnPD-L1-S195A-HA, SPnPD-L1-S195E-HA, SPoptPD-L1-S195A-HA, and SPoptPD-L1-S195E-HA, the S195A and S195E sequences were generated from synthetic genes (IDT Integrated DNA Technologies) and cloned into the FP5950 or FP5951 plasmids using the BamHI and SbfI restriction sites.

For more information regarding the sequences, please refer to Supplementary Data 2 and for experimental conditions Supplementary Data 3.

### Glycosidase inhibitor treatments

For the deglycosylation digestion, 10 µg of protein extracts were treated according to the manufacturer’s instructions and incubated for 1 h at 37 °C with 500 U of Endo H, 500 U of PNGase F or 50 U O-glycosidase (New England BioLabs).

### CytoRUSH assay western blotting

HeLa cells were grown in 60 mm dishes for 24 h and transfected with plasmidic expression vectors encoding cytosolic streptavidin and SBP-bearing reporter proteins at 1:1 ratio. After 6 h, cells were trypsinised and equally distributed into 24-multiwell plates. After 16 h, cells were treated with biotin (B4501; Sigma-Aldrich) 40 µM, lysed and processed for Western Blot analysis.

### Nascent chain-biotinylation assay

HeLa cells were grown in 35 mm dishes and transiently transfected with plasmid expression vectors encoding cytosolic V5-TurboID-NES and SPn- or SPopt- SBP-EGFP-KDEL at 1:1 ratio. After 24 h, cells were treated with biotin (B4501; Sigma-Aldrich) 150 μM for 10 h, lysed and protein extracts were incubated with streptavidin-coated magnetic beads (Sigma-Aldrich) overnight. Next, magnetic beads were extensively washed with lysis buffer, resuspended in Laemmli buffer, boiled at 95 °C for 5 min, and samples were analysed by SDS-PAGE.

### Immunofluorescence and confocal microscopy

Indirect immunofluorescence was performed as previously described^59–61^. Briefly, cells were grown on a coverslip for 24 h before fixation in 3.7% formaldehyde dissolved in phosphate buffer (PBS) for 30 min at room temperature. Fixation was blocked by incubating cells with ice-cold PBS containing glycine 0.1 M for 5 min and then washed three times with PBS buffer. Next, cells were permeabilized with a blocking buffer (B buffer) containing saponin as a non-ionic detergent (PBS, BSA1%, Saponin 0.05% and sodium azide 0.01%) for 15 min at room temperature. After permeabilization, cells were incubated with primary antibodies diluted in B buffer for 1 hour, washed in PBS for 5 min each, and incubated with secondary antibodies diluted in B buffer for 45 min. Finally, cells were washed three times with PBS and once with deionized distilled water before mounting on a slide. Single confocal images were acquired at 63x magnification on an LSM700 (Carl Zeiss, Jena, Germany). Co-localisation was measured using ImageJ Biophotonics software represented by measuring the Pearson’s Coefficient of 50 cells for each experimental point.

For cytoRUSH immunofluorescence and SPnA18Y-/SPoptA18Y-PD-L1-HA experiments, HeLa cells were transfected using calcium phosphate, as described previously^56^. The localisation of the PD-L1 construct was analyzed 24 h after transfection. For this, cells were fixed with 3% paraformaldehyde in PBS for 14 min at room temperature. Cells were permeabilised with Saponin 1x in PBS 15 min at room temperature. Cells were labelled with the appropriate primary antibodies overnight at 4 °C in PBS-supplemented Saponin 1x. After washing, cells were incubated 1 h at room temperature with secondary antibodies and DAPI (0.5 µg/mL) in PBS. Coverslips were mounted in Mowiol. Single confocal images were acquired at 63x magnification on an Inverted Leica TCS SP8 MP (Carl Zeiss). Co-localisation was measured using ImageJ Biophotonics software represented by measuring the Pearson’s Coefficient of 50 cells for each experimental point.

For TANGO1 and ERGIC-53 immunofluorescence cells were fixed as previously described. Cells were permeabilised with Triton 0,1% in PBS 5 min at room temperature. Cells were labelled with the appropriate primary antibodies overnight at 4 °C in PBS.

For surface staining, cells were incubated with an anti-GFP antibody (11814460001; Roche) on ice for 1 hour, then fixed with 2% paraformaldehyde in PBS for 14 min on ice. Then, immunofluorescence was performed as described above.

For quantification, surface EGFP-PD-L1 intensity was measured as the mean fluorescence signal from the anti-GFP staining, while total EGFP-PD-L1 intensity was obtained from the intrinsic EGFP signal. The “relative” intensity corresponds to the ratio between surface and total EGFP–PD-L1 intensity for each cell.

### Real-time imaging of the synchronised secretion

HeLa cells expressing RUSH constructs were grown on 25-mm glass coverslips. Before imaging, coverslips were transferred to an L-shape tubing-equipped Chamlide chamber (Live Cell Instrument). Trafficking was induced by exchanging Leibovitz’s L-15 medium (Life Technologies) with prewarmed Leibovitz’s L-15 medium supplemented with 40 μM biotin (ref B4501; Sigma-Aldrich). Imaging was performed at 37 °C in a thermostat-controlled chamber using an Eclipse 80i microscope (Nikon) equipped with a spinning-disk confocal head (Perkin Elmer) and an Ultra897 iXon camera (Andor). Image acquisition was performed using MetaMorph software (Molecular Devices).

### Preparation of cell extracts, immunoprecipitation SDS-PAGE and western blotting

Preparation of cell extracts, SDS-PAGE and Western blot analysis were performed as previously detailed^62,63^. Briefly, total protein cell extracts were performed using a B-Buffer (50 mM HEPES, 150 mM NaCl, 1 mM EDTA, 1 mM EGTA, 10% glycerol and 1% Triton-X-100) supplemented with protease and phosphatase inhibitors. Protein concentration was measured by the Bradford assay. Protein samples were prepared by using Laemmli buffer 3X (150 mM Tris-HCl pH 6.8, SDS 6%, Glycerol 30%, 0.006% Bromophenol Blue) in the presence or absence of the reducing agent ß-Merkaptoethanol (30% ß-M). Proteins were separated on SDS gels and transferred to PVDF membranes. Membranes were then treated with a blocking buffer (25 mM Tris, pH 7.4, 200 mM NaCl, 0.5%, 0.025% TWEEN20) containing 5% non-fat powdered milk and incubated overnight with primary antibodies. Membranes were finally incubated with an HRP-conjugated secondary antibody, and chemiluminescence was determined using the ECL detection system. Densitometric analysis was performed using the NIH Image software (Bethesda, MD, USA).

For immunoprecipitation from HeLa stably expressing PD-L1, cells were harvested in 200 μL of EBC lysis buffer (0.5% Nonidet NP-40, 50 mM Tris-Cl [pH 8.0], 120 mM NaCl, 5 mM EDTA, 50 mM Hepes) supplemented with protease and phosphatase inhibitors. After 1 h incubation in lysis buffer at 4 °C, the lysates were then centrifuged for 10 min at 13,200 rpm at 4 °C, and the protein concentration was determined using a Pierce BCA Protein Assay kit (Thermo Scientific). For each sample, 2 mg of protein was incubated with 50 μL of Pierce Anti-HA Magnetic Beads (Thermo Scientific) according to the manufacturer’s instructions. The immunoprecipitated proteins were then deglycosylated with 500 U of PNGase F (New England Biolabs) for 1  h at 37 °C. The immunoprecipitated proteins were then eluted by adding 30 μL of 3X Laemmli Buffer and incubating for 5 min at 95 °C. Initial lysates and immunoprecipitated proteins were analysed by SDS-PAGE and immunoblotted with specific antibodies.

### EGFP secretion assay

GB138 cells were grown in 60 mm dishes and transiently transfected with plasmid expression vectors encoding SPn- or SPopt-EGFP. After 24 h, cells were split into a 12-well plate and incubated for 24 h at 37 °C. Equal amounts (100 μL) of the culture media^64^ from each well were collected at different time points (from 0 to 5 h), and EGFP fluorescent signal intensity was measured by using a plate reader spectrofluorometer. Intracellular EGFP signal levels (IN) from each corresponding well were also determined from total cell extracts. EGFP secretion rate was represented as the OUT/IN ratio.

### Cleavage prediction

To investigate whether optimising the H-region of PD-L1’s SP affects its cleavage, we conducted a cleavage prediction using two servers: SignalP 6.0^32^, which can predict all types of signal peptides and cleavage site, and Phobius, which can identify transmembrane domains that may be mistaken for SPs, in addition to detecting true SPs.

### Quantification and statistical analysis

All the results are obtained from independent experiments. Statistical analysis was performed as indicated in each figure. *P* values are shown as asterisks: **** for *P* value < 0.0001, *** for *P* value < 0.001, ** for *P* value < 0.01, * for *P* value < 0.05 and ns for data not statistically significant.

### Bioinformatic analysis of human signal peptides

All human protein entries carrying a signal peptide (SP) were retrieved from the Signal Peptide Database (SPdb; (http://www.signalpeptide.de/?m=searchspdb)) and their up-to-date full-length canonical sequences were obtained from the UniProt resource (https://www.uniprot.org/id-mapping). For each protein, the H-region was predicted using SignalP 6.0 (https://services.healthtech.dtu.dk/services/SignalP-6.0/) with the following settings:

Organism: Eukarya / Model mode: Slow / Output format: Long output

The hydrophobicity of each predicted H-region was calculated using the Kyte-Doolittle hydropathy scale^65^. Individual residue scores were averaged across the H-region to obtain a hydrophobicity index for each SP. SPs were then ranked from least to most hydrophobic based on their H-region Kyte–Doolittle values.

## Supplementary information

Supplementary Information

Description of Additional Supplementary Information

Supplementary Data 1

Supplementary Data 2

Supplementary Data 3

Supplementary Data 4

Movie S1

Movie S2

Movie S3

Movie S4

Movie S5

Movie S6

Transparent Peer Review file

## Source data

Source Data

## Data Availability

All data are available in the article and supplementary information. The source data for all data presented in the figures are provided with this paper as a Source Data file. Original uncropped gels are provided in the supplementary information file. Material is available upon request. The presented research complies with all relevant ethical regulations. Source data are provided with this paper.

## References

[1] Juszkiewicz, S. & Hegde, R. S. Quality control of orphaned proteins. *Mol. Cell* **71**, 443–457 (2018). 30075143 10.1016/j.molcel.2018.07.001 PMC6624128

[2] Wenzell, N. A. et al. Global signal peptide profiling reveals principles of selective Sec61 inhibition. *Nat. Chem. Biol.* **20**, 1154–1163 (2024). 38519575 10.1038/s41589-024-01592-7

[3] von Heijne, G. Signal sequences. * limits Var. J. Mol. Biol.* **184**, 99–105 (1985). 10.1016/0022-2836(85)90046-4 4032478

[4] Zimmermann, R., Eyrisch, S., Ahmad, M. & Helms, V. Protein translocation across the ER membrane. *Biochim Biophys. Acta* **1808**, 912–924 (2011). 20599535 10.1016/j.bbamem.2010.06.015

[5] Hosomi, A. et al. The ER-associated protease Ste24 prevents N-terminal signal peptide-independent translocation into the endoplasmic reticulum in Saccharomyces cerevisiae. *J. Biol. Chem.* **295**, 10406–10419 (2020). 32513868 10.1074/jbc.RA120.012575 PMC7383372

[6] Sun, S., Li, X. & Mariappan, M. Signal sequences encode information for protein folding in the endoplasmic reticulum. *J. Cell Biol.* **222**, e202203070 (2023). 36459117 10.1083/jcb.202203070 PMC9723807

[7] Perara, E., Rothman, R. E. & Lingappa, V. R. Uncoupling translocation from translation: implications for transport of proteins across membranes. *Science* **232**, 348–352 (1986). 3961485 10.1126/science.3961485

[8] Kim, S. J., Mitra, D., Salerno, J. R. & Hegde, R. S. Signal sequences control gating of the protein translocation channel in a substrate-specific manner. *Dev. Cell* **2**, 207–217 (2002). 11832246 10.1016/s1534-5807(01)00120-4

[9] Ainger, K. J. & Meyer, D. I. Translocation of nascent secretory proteins across membranes can occur late in translation. *EMBO J.* **5**, 951–955 (1986). 3087745 10.1002/j.1460-2075.1986.tb04308.x PMC1166887

[10] Hatsuzawa, K., Tagaya, M. & Mizushima, S. The hydrophobic region of signal peptides is a determinant for SRP recognition and protein translocation across the ER membrane. *J. Biochem* **121**, 270–277 (1997). 9089400 10.1093/oxfordjournals.jbchem.a021583

[11] Pardoll, D. M. The blockade of immune checkpoints in cancer immunotherapy. *Nat. Rev. Cancer* **12**, 252–264 (2012). 22437870 10.1038/nrc3239 PMC4856023

[12] Li, C.-W. et al. Glycosylation and stabilization of programmed death ligand-1 suppresses T-cell activity. *Nat. Commun.* **7**, 12632 (2016). 27572267 10.1038/ncomms12632 PMC5013604

[13] Cha, J.-H. et al. Metformin Promotes Antitumor Immunity via Endoplasmic-Reticulum-Associated Degradation of PD-L1. *Mol. Cell* **71**, 606–620.e7 (2018). 30118680 10.1016/j.molcel.2018.07.030 PMC6786495

[14] Chan, L.-C. et al. IL-6/JAK1 pathway drives PD-L1 Y112 phosphorylation to promote cancer immune evasion. *J. Clin. Invest* **129**, 3324–3338 (2019). 31305264 10.1172/JCI126022 PMC6668668

[15] Zhang, X. et al. NEK2 inhibition triggers anti-pancreatic cancer immunity by targeting PD-L1. *Nat. Commun.* **12**, 4536 (2021). 34315872 10.1038/s41467-021-24769-3 PMC8316469

[16] Hsu, J.-M., Li, C.-W., Lai, Y.-J. & Hung, M.-C. Posttranslational Modifications of PD-L1 and Their Applications in Cancer Therapy. *Cancer Res* **78**, 6349–6353 (2018). 30442814 10.1158/0008-5472.CAN-18-1892 PMC6242346

[17] Zheng, T. & Nicchitta, C. V. Structural determinants for signal sequence function in the mammalian endoplasmic reticulum. *J. Biol. Chem.* **274**, 36623–36630 (1999). 10593964 10.1074/jbc.274.51.36623

[18] Nilsson, I. et al. The code for directing proteins for translocation across ER membrane: SRP cotranslationally recognizes specific features of a signal sequence. *J. Mol. Biol.* **427**, 1191–1201 (2015). 24979680 10.1016/j.jmb.2014.06.014 PMC4277940

[19] Celińska, E., Borkowska, M., Białas, W., Korpys, P. & Nicaud, J.-M. Robust signal peptides for protein secretion in Yarrowia lipolytica: identification and characterization of novel secretory tags. *Appl Microbiol Biotechnol.* **102**, 5221–5233 (2018). 29704042 10.1007/s00253-018-8966-9 PMC5959983

[20] Barrette-Ng, I. H., Wu, S.-C., Tjia, W.-M., Wong, S.-L. & Ng, K. K. S. The structure of the SBP-Tag–streptavidin complex reveals a novel helical scaffold bridging binding pockets on separate subunits. *Acta Crystallogr D. Biol. Crystallogr* **69**, 879–887 (2013). 23633599 10.1107/S0907444913002576 PMC3640474

[21] White, S. H. & Von Heijne, G. How Translocons Select Transmembrane Helices. *Annu. Rev. Biophys.* **37**, 23–42 (2008). 18573071 10.1146/annurev.biophys.37.032807.125904

[22] Lakkaraju, A. K. K. et al. Efficient secretion of small proteins in mammalian cells relies on Sec62-dependent posttranslational translocation. *Mol. Biol. Cell* **23**, 2712–2722 (2012). 22648169 10.1091/mbc.E12-03-0228 PMC3395660

[23] Haßdenteufel, S., Nguyen, D., Helms, V., Lang, S. & Zimmermann, R. ER import of small human presecretory proteins: components and mechanisms. *FEBS Lett.* **593**, 2506–2524 (2019). 31325177 10.1002/1873-3468.13542

[24] Zhou, G. et al. Role of AMP-activated protein kinase in mechanism of metformin action. *J. Clin. Invest.* **108**, 1167–1174 (2001). 11602624 10.1172/JCI13505 PMC209533

[25] Cool, B. et al. Identification and characterization of a small molecule AMPK activator that treats key components of type 2 diabetes and the metabolic syndrome. *Cell Metab.* **3**, 403–416 (2006). 16753576 10.1016/j.cmet.2006.05.005

[26] Dai, R. Y. et al. Implication of transcriptional repression in compound C-induced apoptosis in cancer cells. *Cell Death Dis.* **4**, e883 (2013). 24157877 10.1038/cddis.2013.419 PMC3920957

[27] Kai, Y., Kawano, Y., Yamamoto, H. & Narahara, H. A possible role for AMP-activated protein kinase activated by metformin and AICAR in human granulosa cells. *Reprod. Biol. Endocrinol.* **13**, 27 (2015). 25889494 10.1186/s12958-015-0023-2 PMC4397678

[28] Pascarella, A. et al. Vacuolated PAS-positive lymphocytes as an hallmark of Pompe disease and other myopathies related to impaired autophagy. *J. Cell Physiol.* **233**, 5829–5837 (2018). 29215735 10.1002/jcp.26365

[29] Dite, T. A. et al. AMP-activated protein kinase selectively inhibited by the type II inhibitor SBI-0206965. *J. Biol. Chem.* **293**, 8874–8885 (2018). 29695504 10.1074/jbc.RA118.003547 PMC5995511

[30] Branon, T. C. et al. Efficient proximity labeling in living cells and organisms with TurboID. *Nat. Biotechnol.* **36**, 880–887 (2018). 30125270 10.1038/nbt.4201 PMC6126969

[31] Goder, V. & Spiess, M. Molecular mechanism of signal sequence orientation in the endoplasmic reticulum. *EMBO J.* **22**, 3645–3653 (2003). 12853479 10.1093/emboj/cdg361 PMC165631

[32] Teufel, F. et al. SignalP 6.0 predicts all five types of signal peptides using protein language models. *Nat. Biotechnol.* **40**, 1023–1025 (2022). 34980915 10.1038/s41587-021-01156-3 PMC9287161

[33] Käll, L., Krogh, A. & Sonnhammer, E. L. L. A combined transmembrane topology and signal peptide prediction method. *J. Mol. Biol.* **338**, 1027–1036 (2004). 15111065 10.1016/j.jmb.2004.03.016

[34] Choo, K. H. & Ranganathan, S. Flanking signal and mature peptide residues influence signal peptide cleavage. *BMC Bioinforma.* **9**, S15 (2008). 10.1186/1471-2105-9-S12-S15 PMC2638155 19091014

[35] Boncompain, G. et al. Synchronization of secretory protein traffic in populations of cells. *Nat. Methods* **9**, 493–498 (2012). 22406856 10.1038/nmeth.1928

[36] Gasparotto, D. et al. Mapping cryptic phosphorylation sites in the human proteome. *EMBO J.* **44**, 6704–6731 (2025). 41044218 10.1038/s44318-025-00567-1 PMC12624043

[37] Walter, P. & Blobel, G. Translocation of proteins across the endoplasmic reticulum III. Signal recognition protein (SRP) causes signal sequence-dependent and site-specific arrest of chain elongation that is released by microsomal membranes. *J. Cell Biol.* **91**, 557–561 (1981). 7309797 10.1083/jcb.91.2.557 PMC2111983

[38] Lakkaraju, A. K. K., Mary, C., Scherrer, A., Johnson, A. E. & Strub, K. SRP keeps polypeptides translocation-competent by slowing translation to match limiting ER-targeting sites. *Cell* **133**, 440–451 (2008). 18455985 10.1016/j.cell.2008.02.049 PMC2430734

[39] Chartron, J. W., Hunt, K. C. L. & Frydman, J. Cotranslational signal-independent SRP preloading during membrane targeting. *Nature* **536**, 224–228 (2016). 27487213 10.1038/nature19309 PMC5120976

[40] Meyer, H. A. et al. Mammalian Sec61 is associated with Sec62 and Sec63. *J. Biol. Chem.* **275**, 14550–14557 (2000). 10799540 10.1074/jbc.275.19.14550

[41] Fons, R. D., Bogert, B. A. & Hegde, R. S. Substrate-specific function of the translocon-associated protein complex during translocation across the ER membrane. *J. Cell Biol.* **160**, 529–539 (2003). 12578908 10.1083/jcb.200210095 PMC2173754

[42] Tyedmers, J. et al. Homologs of the yeast Sec complex subunits Sec62p and Sec63p are abundant proteins in dog pancreas microsomes. *Proc. Natl. Acad. Sci. USA* **97**, 7214–7219 (2000). 10860986 10.1073/pnas.97.13.7214 PMC16525

[43] Haßdenteufel, S. et al. Chaperone-mediated sec61 channel gating during er import of small precursor proteins overcomes sec61 inhibitor-reinforced energy barrier. *Cell Rep.* **23**, 1373–1386 (2018). 29719251 10.1016/j.celrep.2018.03.122 PMC5946456

[44] Ziska, A. et al. The signal peptide plus a cluster of positive charges in prion protein dictate chaperone-mediated Sec61 channel gating. *Biol. Open* **8**, bio040691 (2019). 30745438 10.1242/bio.040691 PMC6451349

[45] Nguyen, D. et al. Proteomics reveals signal peptide features determining the client specificity in human TRAP-dependent ER protein import. *Nat. Commun.* **9**, 3765 (2018). 30217974 10.1038/s41467-018-06188-z PMC6138672

[46] Schorr, S. et al. Identification of signal peptide features for substrate specificity in human Sec62/Sec63-dependent ER protein import. *FEBS J.* **287**, 4612–4640 (2020). 32133789 10.1111/febs.15274

[47] Gamerdinger, M., Hanebuth, M. A., Frickey, T. & Deuerling, E. The principle of antagonism ensures protein targeting specificity at the endoplasmic reticulum. *Science* **348**, 201–207 (2015). 25859040 10.1126/science.aaa5335

[48] Ullers, R. S. et al. Interplay of signal recognition particle and trigger factor at L23 near the nascent chain exit site on the Escherichia coli ribosome. *J. Cell Biol.* **161**, 679–684 (2003). 12756233 10.1083/jcb.200302130 PMC2199365

[49] Lee, H. C. & Bernstein, H. D. Trigger factor retards protein export in Escherichia coli. *J. Biol. Chem.* **277**, 43527–43535 (2002). 12205085 10.1074/jbc.M205950200

[50] Patzelt, H. et al. Binding specificity of *Escherichia coli* trigger factor. *Proc. Natl. Acad. Sci. Usa.* **98**, 14244–14249 (2001). 11724963 10.1073/pnas.261432298 PMC64667

[51] Qin, W. et al. The diverse function of pd-1/pd-l pathway beyond cancer. *Front Immunol.* **10**, 2298 (2019). 31636634 10.3389/fimmu.2019.02298 PMC6787287

[52] Besemer, J. et al. Selective inhibition of cotranslational translocation of vascular cell adhesion molecule 1. *Nature* **436**, 290–293 (2005). 16015337 10.1038/nature03670

[53] Vitale, F. et al. A light-resuming strategy as a screening method for selecting Sec61 inhibitors down-modulating PD-L1 expression. *Nat. Commun.* **16**, 7243 (2025). 40770233 10.1038/s41467-025-62439-w PMC12328631

[54] Kroonen, J. et al. Human glioblastoma-initiating cells invade specifically the subventricular zones and olfactory bulbs of mice after striatal injection. *Int J. Cancer* **129**, 574–585 (2011). 20886597 10.1002/ijc.25709

[55] Brodsky, J. L., Goeckeler, J. & Schekman, R. BiP and Sec63p are required for both co- and posttranslational protein translocation into the yeast endoplasmic reticulum. *Proc. Natl. Acad. Sci. USA* **92**, 9643–9646 (1995). 7568189 10.1073/pnas.92.21.9643 PMC40858

[56] Jordan, M., Schallhorn, A. & Wurm, F. M. Transfecting mammalian cells: optimization of critical parameters affecting calcium-phosphate precipitate formation. *Nucleic Acids Res.* **24**, 596–601 (1996). 8604299 10.1093/nar/24.4.596 PMC145683

[57] D’Arrigo, P. et al. A regulatory role for the co-chaperone FKBP51s in PD-L1 expression in glioma. *Oncotarget* **8**, 68291–68304 (2017). 28978117 10.18632/oncotarget.19309 PMC5620257

[58] Abraham, O. et al. Control of protein trafficking by reversible masking of transport signals. *MBoC* **27**, 1310–1319 (2016). 26941332 10.1091/mbc.E15-07-0472 PMC4831884

[59] D’Agostino, M. et al. ER reorganization is remarkably induced in cos-7 cells accumulating transmembrane protein receptors not competent for export from the endoplasmic reticulum. *J. Membr. Biol.* **247**, 1149–1159 (2014). 25086772 10.1007/s00232-014-9710-8

[60] Caiazza, C. et al. Effects of long-term citrate treatment in the pc3 prostate cancer cell line. *IJMS* **20**, 2613 (2019). 31141937 10.3390/ijms20112613 PMC6600328

[61] Allocca, S. et al. An αB-Crystallin Peptide Rescues Compartmentalization and Trafficking Response to Cu Overload of ATP7B-H1069Q, the Most Frequent Cause of Wilson Disease in the Caucasian Population. *IJMS* **19**, 1892 (2018). 29954118 10.3390/ijms19071892 PMC6073935

[62] D’Agostino, M. et al. Unconventional secretion of α-Crystallin B requires the Autophagic pathway and is controlled by phosphorylation of its serine 59 residue. *Sci. Rep.* **9**, 16892 (2019). 31729431 10.1038/s41598-019-53226-x PMC6858465

[63] D’Agostino, M., Risselada, H. J., Endter, L. J., Comte-Miserez, V. & Mayer, A. SNARE -mediated membrane fusion arrests at pore expansion to regulate the volume of an organelle. * EMBO J.* **37**, e99193 (2018). 30120144 10.15252/embj.201899193 PMC6166129

[64] Xie, W. et al. A Luciferase Reporter Gene System for High-Throughput Screening of γ-Globin Gene Activators. in *High Throughput Screening* (ed. Janzen, W. P.) vol. 1439 207–226 (Springer New York, New York, NY, 2016). 10.1007/978-1-4939-3673-1_14 27316998

[65] Kyte, J. & Doolittle, R. F. A simple method for displaying the hydropathic character of a protein. *J. Mol. Biol.* **157**, 105–132 (1982). 7108955 10.1016/0022-2836(82)90515-0

