# Supplementary material for: A delayed translocation into the endoplasmic reticulum controls the post-translational modifications of PD-L1
Source: Nat Commun. 2026 Apr 11;17:5059. doi: 10.1038/s41467-026-71760-x (PMC13243477; doi:10.1038/s41467-026-71760-x)
Supplement: Supplementary file 1 — Supplementary Information [file 41467_2026_71760_MOESM1_ESM.pdf]

Supplementary Information for

A delayed translocation into the endoplasmic reticulum controls the post-translational modifications of PD-L1.

Magda Cannata Serio[1]†, Fulvia Vitale[2]†, Gianluca Scerra[2], Raffaella Bonavita[2], Patrick Poullet[3], Maria Gabriella Caporaso[2], Laura Marrone[2], Simona Romano[2], Maurizio Renna[2], Franck Perez[1], Massimo D'Agostino[2]

[1] Institut Curie, PSL Research University, CNRS UMR144, Paris, France.

[2] Department of Molecular Medicine and Medical Biotechnology, University of Naples Federico II, Italy.

[3] Institut Curie, Bioinformatics core facility (CUBIC), INSERM U1331, PSL Research University, Mines Paris Tech, Paris, France.

†These authors contributed equally to this work.

Corresponding authors. Email: massimo.dagostino@unina.it, franck.perez@curie.fr and magda.cannata-serio@curie.fr

The PDF file includes:

Fig. S1 to 11

Tables S1 to S2

Uncropped blots

Fig. S1. Analysis and optimization of the PD-L1 signal peptide.

(A) Multi-alignment of PD-L1 SPs among species. Amino acid residues highlighted in yellow indicate the H-region. Polar and charged residues are indicated in red. **(B, C)** Analysis of the hydrophobicity index of the H-regions of PD-L1 SPs belonging to 91 different species in comparison to the SPs of a set of 100 randomly selected secreted proteins (B) and 100 randomly selected transmembrane proteins (C) using the Kyte-Doolittle scale. **(D)** Representation of the topology of PD-L1 and the PTMs modulating its intracellular fate. N-linked glycosylation; P, phosphorylation. All data are mean ± SEM; statistical significance was determined by Student's t test. Levels of significance: **** P value < 0.0001.

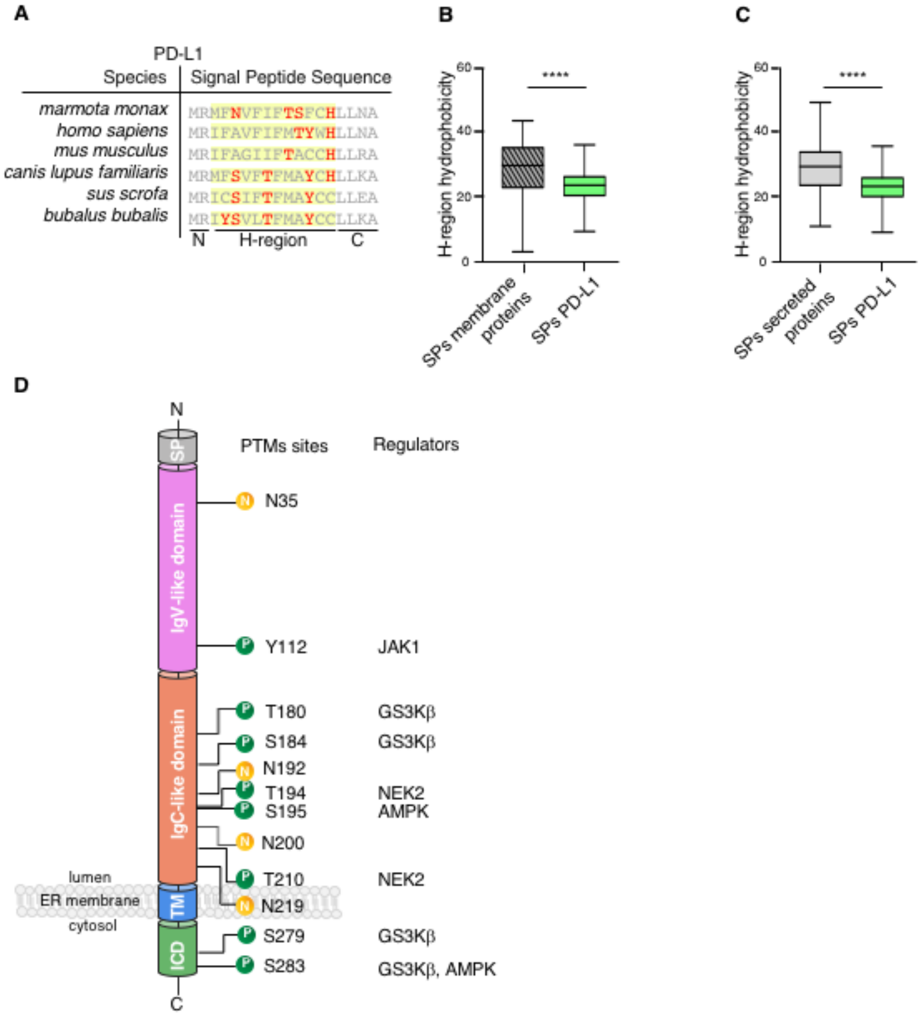

Fig. S2. Stepwise mutagenesis of the PD-L1 SP and its impact on traffic and glycosylation. **(A)** Schematic representation of the stepwise mutagenesis performed in the H-region of the PD-L1 SP. Three polar/charged residues (T11, Y12, H14) were individually or combinatorially replaced by leucines, generating 1L (T11L), 2L (T11L/Y12L), and 3L (T11L/Y12L/H14L, corresponding to SPopt). For each variant, the corresponding h-region hydrophobicity is indicated. **(B, C)** Confocal microscopy analysis of HeLa cells stably expressing PD-L1 carrying SPn, 1L, 2L or 3L-SPopt, stained with the ER marker RTN4 and Golgi marker Giantin. Expression of PD-L1 was analyzed by using a specific antibody against the HA tag. Single focal sections are shown. Scale bar: 20 μm. Images are representative of three independent experiments (n = 30 cells). **(D)** Total cell extracts of HeLa cells stably expressing PD-L1 carrying SPn, 1L, 2L or 3L-SPopt were separated on SDS-PAGE before and after EndoH, PNGaseF and O-glycosidase digestion. Detection was performed using specific antibodies against HA tag. Vinculin was used as a loading control. G: Glycosylated PD-L1, PG: partially glycosylated PD-L1, N: native PD-L1. All data are representative of n = 3 independent experiments.

A

Signal Peptide	Sequence	H-region hydrophobicity
PD-L1 (SPn):	MRIFAVFIFMTYWHLLNA	23
PD-L1 (1L):	MRIFAVFIFMLYWHLLNA	27,5
PD-L1 (2L):	MRIFAVFIFMLLWHLLNA	32,6
PD-L1 (3L-SPopt):	MRIFAVFIFMLLWLLLNA	39,6

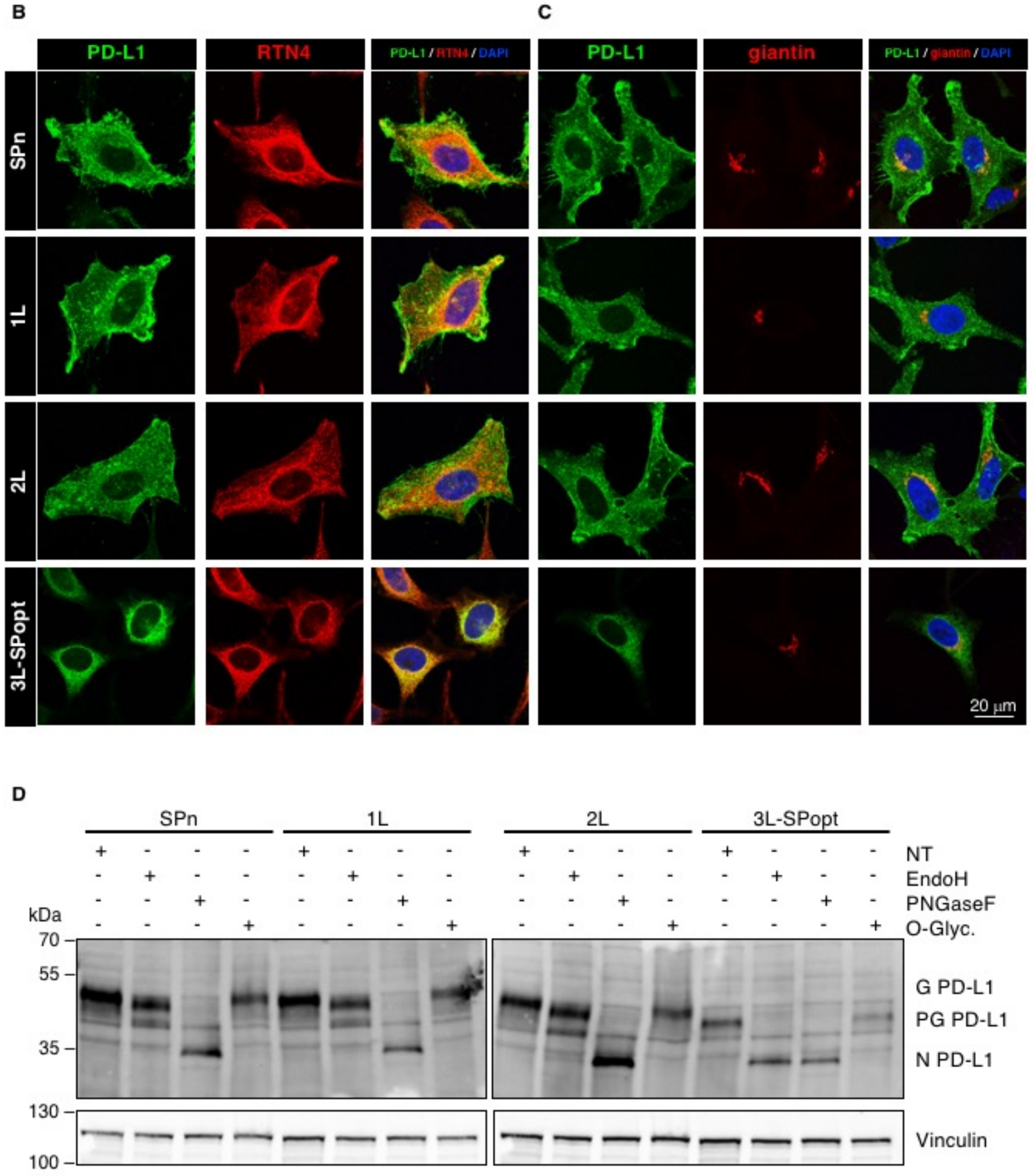

Fig. S3. Effect of cytoRUSH assay on SPn-SBP-EGFP-PD-L1 and ER-resident reporter protein.

(A) HeLa cells were transiently transfected with SPn- or SPopt-SBP-EGFP-PD-L1 expression vectors in combination with the cytosolic streptavidin in a ratio 1:1 for 24 hours. After that, cells were treated with biotin (40 μM) for 6 hours and PD-L1 protein levels were analyzed by SDS-PAGE. PD-L1 and streptavidin proteins were revealed using specific antibodies against EGFP and streptavidin, respectively. **(B)** Expression levels of PD-L1 relative to those of β-actin were calculated. **(C)** GB138 were transiently transfected with SPn-SBP-EGFP-PDL1 in the presence or absence of cytosolic streptavidin for 24 hours. Cells were then treated with either 40 μM of biotin or 5 μM of MG132, or both. Total cell extracts were obtained and subjected to immunoblotting analysis using antibodies against β-actin, streptavidin and EGFP. **(D)** Protein levels of each construct under different conditions were normalized to the protein levels under untreated conditions. **(E)** HeLa cells were transiently transfected with the cytosolic streptavidin and SPn-SBP-EGFP-KDEL or SPopt-SBP-EGFP-KDEL in a ratio of 1:1 for 24 hours. Protein levels were analyzed by SDS-PAGE before and after 8 hours of biotin treatment (40 μM). Specific antibodies were used to detect EGFP-KDEL, streptavidin and Calnexin proteins. **(F)** Expression levels of EGFP-KDEL relative to those of Calnexin were calculated. All data are mean ± SD; statistical significance was determined by Student's t test with data from n = 3 independent experiments. Levels of significance: **** $P < 0.0001$; *** $P < 0.001$; ns, not significant.

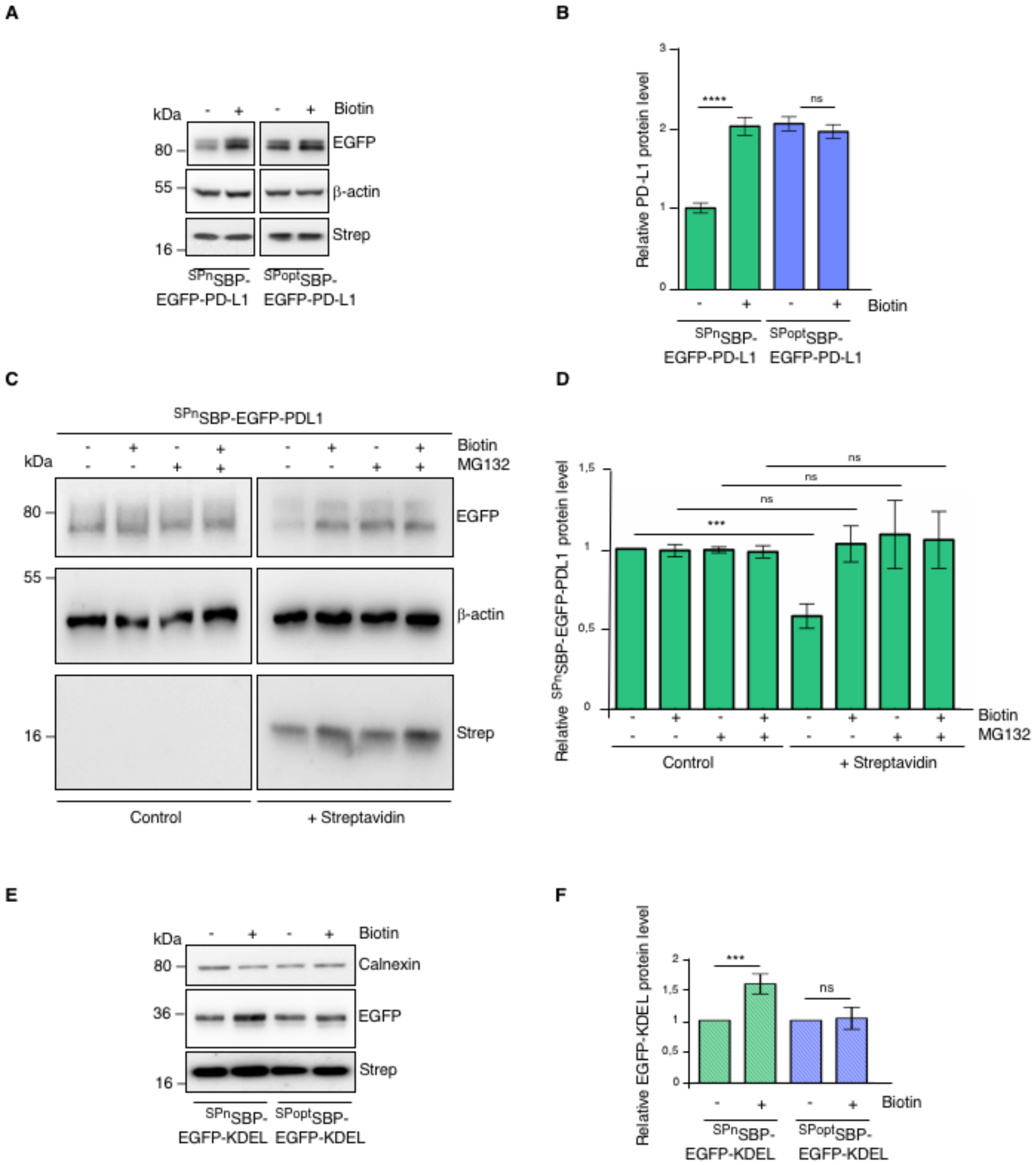
A
kDa
Biotin
EGFP
β-actin
Strep
80
55
16
SPnSBP-EGFP-PD-L1
SPoptSBP-EGFP-PD-L1
B
Relative PD-L1 protein level
****
ns
Biotin
SPnSBP-EGFP-PD-L1
SPoptSBP-EGFP-PD-L1
C
SPnSBP-EGFP-PDL1
Biotin
MG132
kDa
EGFP
β-actin
Strep
80
55
16
Control
+ Streptavidin
D
Relative SPnSBP-EGFP-PDL1 protein level
***
ns
ns
ns
Biotin
MG132
Control
+ Streptavidin
E
kDa
Biotin
Calnexin
EGFP
Strep
80
36
16
SPnSBP-EGFP-KDEL
SPoptSBP-EGFP-KDEL
F
Relative EGFP-KDEL protein level
***
ns
Biotin
SPnSBP-EGFP-KDEL
SPoptSBP-EGFP-KDEL

Fig. S4. CytoRUSH assay to discriminate the mode of translocation.

(A) Schematic representation of the SPs variants cloned into the cytoRUSH constructs. For each variant, the corresponding h-region hydrophobicity is indicated. **(B)** HeLa cells were transiently transfected for 24 h with SBP-EGFP-PD-L1 constructs containing the indicated signal peptides, together with cytosolic streptavidin at a 1:1 ratio. Where indicated, cells were treated overnight with 40 µM biotin (o.n. biotin). Cell-surface PD-L1 levels were assessed by confocal microscopy using an anti-EGFP antibody on non-permeabilized cells. Single optical sections are shown. Scale bar: 20 µm. **(C)** Quantification of surface EGFP fluorescence intensity. All data are mean ± SEM; statistical significance was determined by Student's t test. Statistical analysis was performed based on results obtained from three independent experiments with n = 10. Levels of significance: **** $P < 0.0001$; *** $P < 0.001$; ** $P < 0.01$; ns, not significant.

A

Signal Peptide	Sequence	H-region hydrophobicity
SPn-PD-L1:	MRIFAVFIFMTYWHLLNA	23
CD44:	MDKFWWHAAWGLCLVPLSLA	16,6
AGAL:	MQLRNPELHLGCALALRFLALVSWDIPGARA	24,4
CD8α:	MALPVTALLLPLALLLHAARP	34,4
IL-2:	MYRMQLLSCIALSLALVTNS	32,2
TRH:	MPGPWLLLALALTLNLTGVPGGRA	22,8
STATH:	MKFLVFAFILALMVSMIGA	37,4
PD-L2:	MIFLLLMLSLELQLHQIAA	23,5

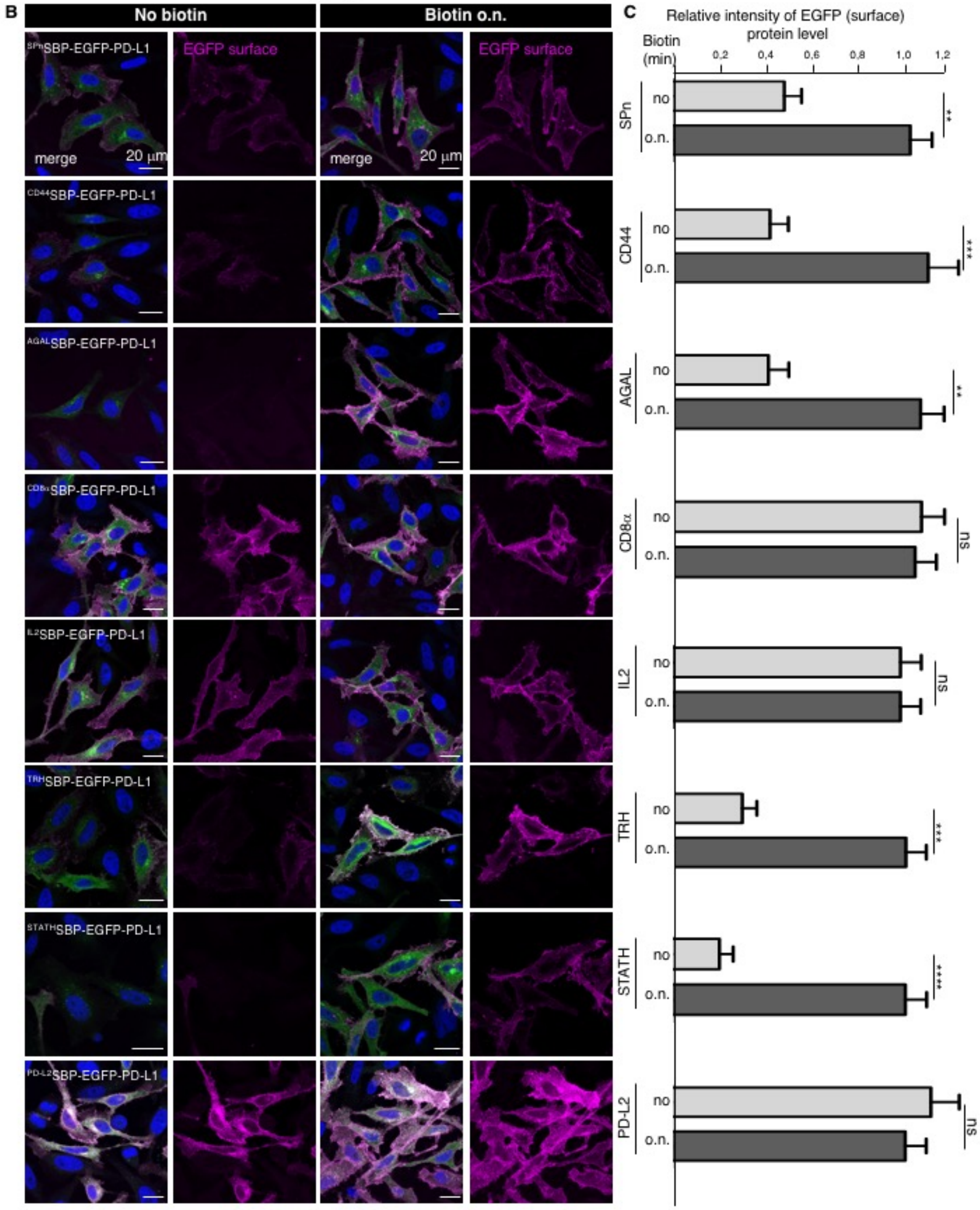

Fig. S5. SP-dependent PD-L1 expression control by pharmacological AMPK modulation. (A) HeLa cells, stably expressing ^{SPn}PD-L1-HA, ^{SPn}PD-L1-HA-S/A, and ^{SPn}PD-L1-HA-S/E, were treated with the indicated concentrations of AMPK activators (A769662 and AICAr) for 16 hours, and PD-L1 protein levels from total cell extracts were analysed by SDS-PAGE using a mouse monoclonal antibody against the HA tag. Calnexin (ER marker) was used as a loading control. (B) HeLa cells, stably expressing ^{SPopt}PD-L1-HA, ^{SPopt}PD-L1-HA-S195A and ^{SPopt}PD-L1-HA-S195E, were treated and handled as in A. (C) HeLa cells, stably expressing PD-L1-HA, PD-L1-HA-S195A and PD-L1-HA-S195E, equipped with either SPn or SPopt, were treated with the indicated concentrations of AMPK inhibitor (AMPKi or SBI-0206965) and PD-L1 protein levels were analysed as in A. All data are mean ± SD; statistical significance was determined by Student’s t test with data from n = 3 independent experiments.

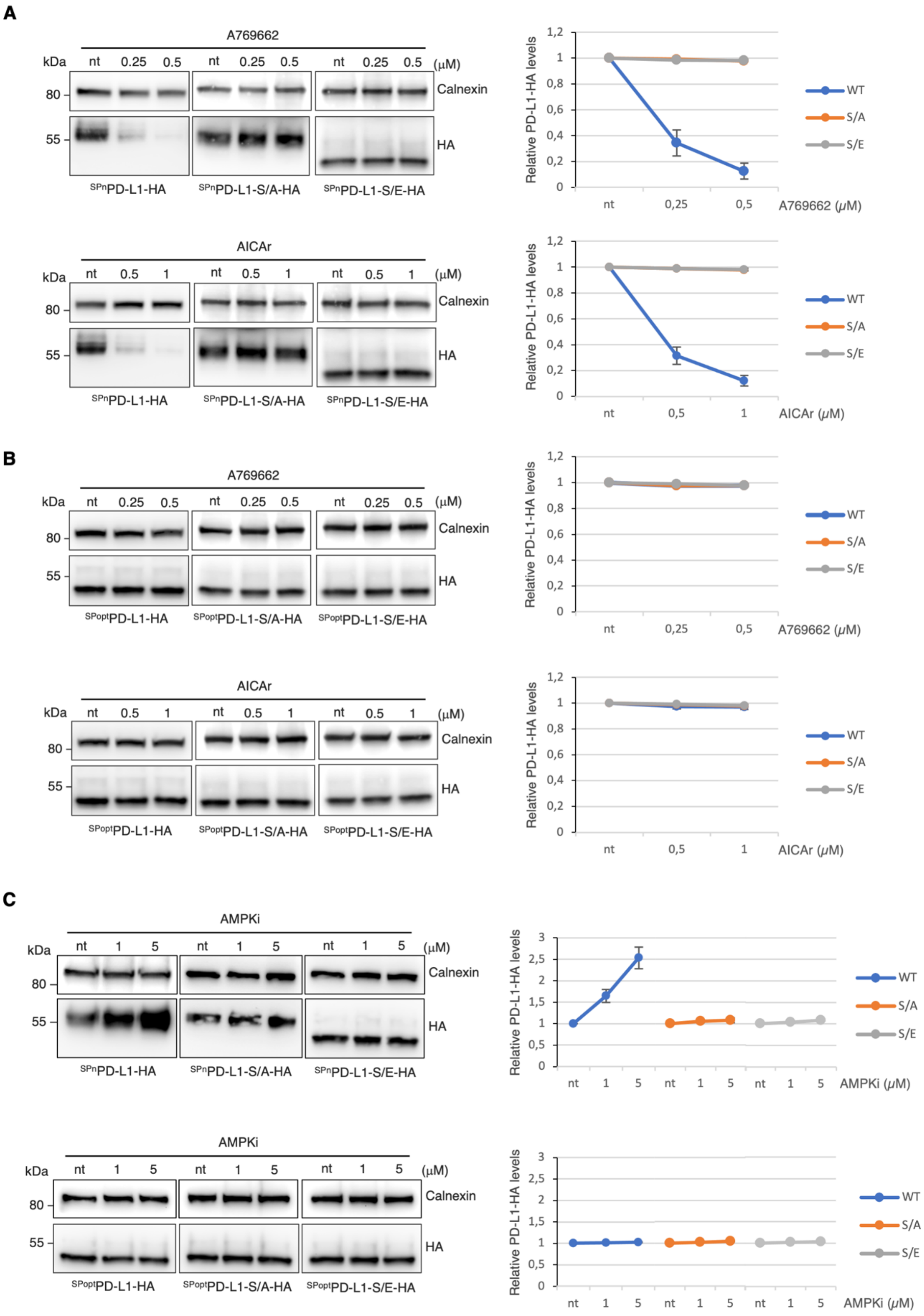
A
A769662
kDa nt 0.25 0.5 nt 0.25 0.5 nt 0.25 0.5 (μM)
80
55
Calnexin
HA
SPnPD-L1-HA
SPnPD-L1-S/A-HA
SPnPD-L1-S/E-HA
Relative PD-L1-HA levels
WT
S/A
S/E
nt 0,25 0,5 A769662 (μM)
AICAr
kDa nt 0.5 1 nt 0.5 1 nt 0.5 1 (μM)
nt 0,5 1 AICAr (μM)
B
SPoptPD-L1-HA
SPoptPD-L1-S/A-HA
SPoptPD-L1-S/E-HA
C
AMPKi
kDa nt 1 5 nt 1 5 nt 1 5 (μM)
nt 1 5 nt 1 5 nt 1 5 AMPKi (μM)

Fig. S6. Analysis of S195 phospho-mutants of PD-L1 and their impact on trafficking and glycosylation.

(A, B) Confocal microscopy analysis of HeLa cells stably expressing PD-L1 carrying ^{SPn}S195, ^{SPn}S195A, ^{SPn}S195E, ^{SPopt}S195, ^{SPopt}S195A, or ^{SPopt}S195E, stained with the ER marker RTN4 (A) or the Golgi marker Giantin (B). Expression of PD-L1 was detected using a specific antibody against the HA tag. Single focal sections are shown. Scale bar: 20 µm. Images are representative of three independent experiments (n = 30 cells). **(C)** Total cell extracts of HeLa cells stably expressing PD-L1 ^{SPn}S195, ^{SPn}S195A, ^{SPn}S195E, ^{SPopt}S195, ^{SPopt}S195A, or ^{SPopt}S195E were separated on SDS-PAGE before and after EndoH, PNGaseF and O-glycosidase digestion. Detection was performed using specific antibodies against the HA tag. Vinculin was used as a loading control. G: glycosylated PD-L1; PG: partially glycosylated PD-L1; N: native PD-L1. Data are representative of n = 3 independent experiments.

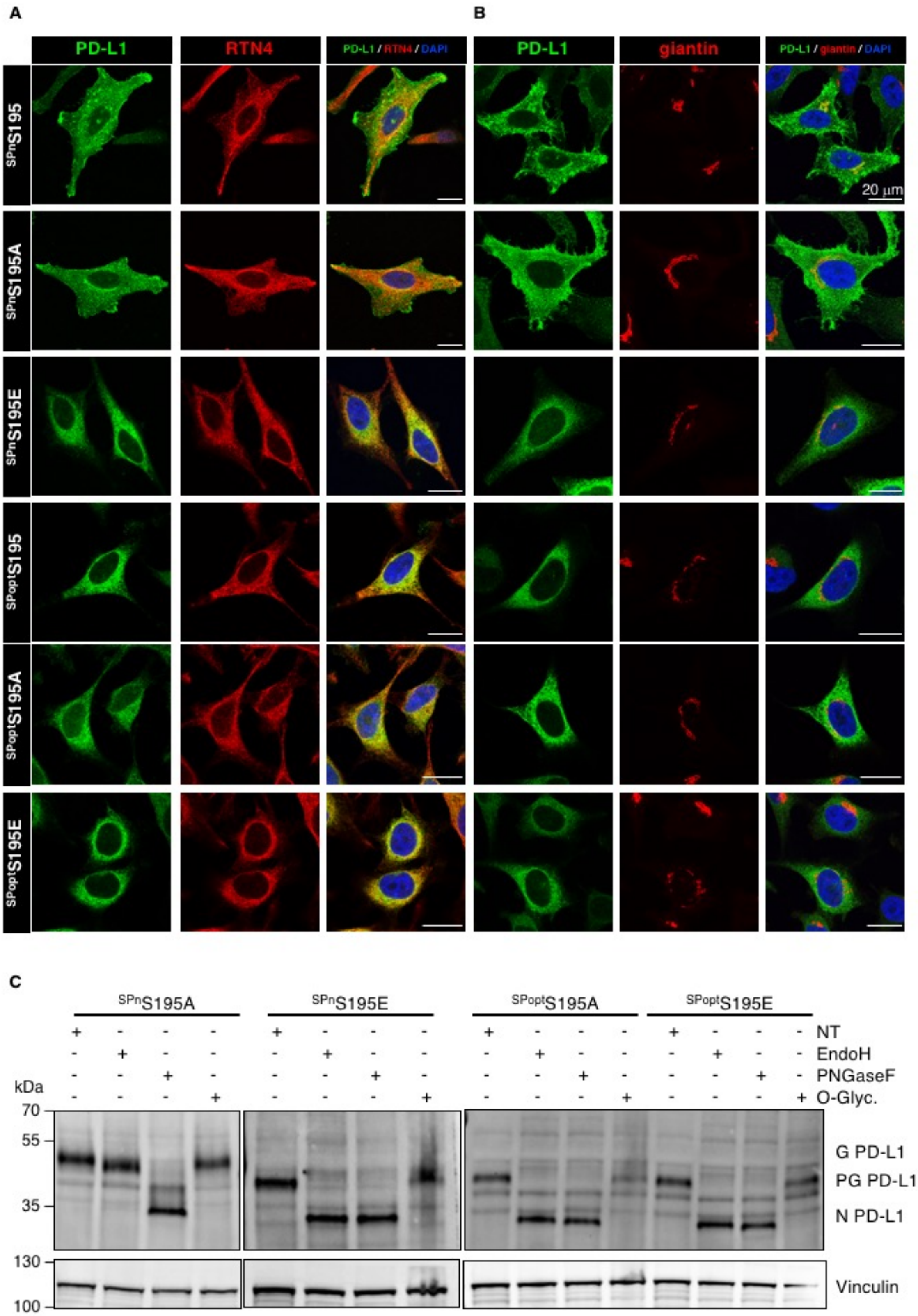
A
B
PD-L1
RTN4
PD-L1 / RTN4 / DAPI
giantin
PD-L1 / giantin / DAPI
SPnS195
SPnS195A
SPnS195E
SPoptS195
SPoptS195A
SPoptS195E
20 μm
C
SPnS195A
SPnS195E
SPoptS195A
SPoptS195E
NT
EndoH
PNGaseF
O-Glyc.
kDa
70
55
35
130
100
G PD-L1
PG PD-L1
N PD-L1
Vinculin

Fig. S7. Effect of slow translation on cytoRUSH assay.

(A) Schematic representation of SP-SBP-EGFP-PD-L1 expressing cassette. **(B)** GB138 cells were transfected with SPn-SBP-EGFP-PD-L1 or SPopt-SBP-EGFP-PD-L1 and treated with low dose of CHX (0.8 µg/mL) for 6 hours with or without biotin (40 µM). PD-L1 protein levels were analyzed by SDS-PAGE and revealed with an anti-EGFP antibody. **(C)** Expression levels of PD-L1 relative to those of streptavidin and β-actin were calculated. Mean values were obtained from three independent experiments. All data are mean ± SD; statistical significance was determined by Student's t test with data from n = 3 independent experiments. Levels of significance: **** $P < 0.0001$; *** $P < 0.001$; ** $P < 0.01$; ns, not significant.

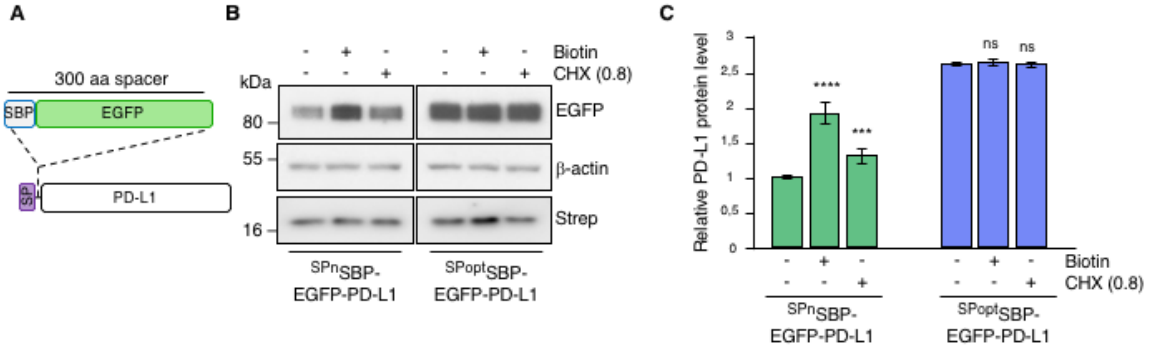

Fig. S8. Effect of the optimization on PD-L1 SP cleavage.

(A, B) Graphs generated through the Phobius server predict the SP, the cleavage site and the different topological regions of the SPn and SPopt-PD-L1-HA proteins. The tables below each graph generated by combining predictions from both the Phobius and SignalP6.0 servers, provide details on the topological domains of PD-L1, along the different regions of the SP (N-, H- and C-regions) and the cleavage site, including their corresponding amino acid positions. **(C)** GB138 cells were transfected with plasmid encoding for SPn- (green line) or SPopt-EGFP (blue line). Equal amounts (100 μL) of the culture media (*12*) from each well were collected at different time points (from 0 to 5 hours) and EGFP fluorescent signal intensity was measured by using a plate reader spectrofluorometer. Intracellular EGFP signal levels (IN) from each corresponding well were determined. EGFP secretion rate was represented as the OUT/IN ratio. All data are mean ± SD. Data are representative of n = 3 independent experiments.

A

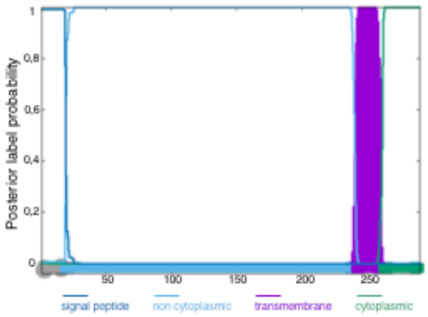

ID	SPn-PDL1			
FT	Signal	1	18	
FT	Region	1	2	N-region
FT	Region	3	14	H-region
FT	Region	15	18	C-region
FT	Topo_dom	19	240	Non cytoplasmic
FT	Transmem	241	261	
FT	Topo_dom	262	299	Cytoplasmic

B

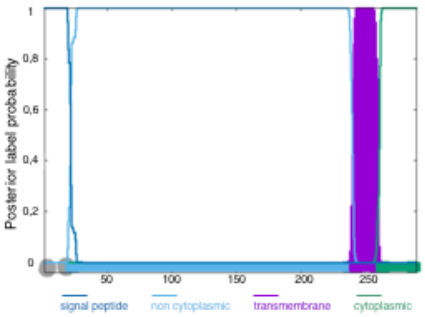

ID	SPopt-PDL1			
FT	Signal	1	18	
FT	Region	1	3	N-region
FT	Region	4	16	H-region
FT	Region	15	18	C-region
FT	Topo_dom	19	240	Non cytoplasmic
FT	Transmem	241	261	
FT	Topo_dom	262	299	Cytoplasmic

C

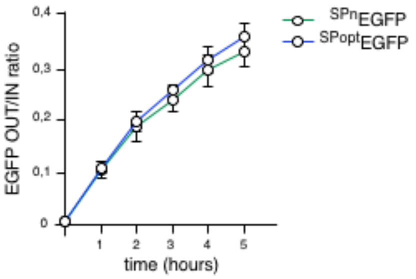

Fig. S9. Mutation of the PD-L1 cleavage site alters its trafficking along the secretory pathway.

(A, B) Graphs generated through the Phobius server predict the SP, the cleavage site and the different topological regions of the SPnA18Y and SPoptA18Y-PD-L1-HA proteins. The tables below each graph generated by combining predictions from both the Phobius and SignalP6.0 servers, provide details on the topological domains of PD-L1, along the different regions of the SP (N-, H- and C-regions) and the cleavage site, including their corresponding amino acid positions. **(C, D)** HeLa cells were transiently transfected with the indicated constructs for 24 hours before being fixed and processed for indirect immunofluorescence. Expression of PD-L1 was analyzed by confocal microscopy by using a specific antibody against the HA tag. Single focal sections are shown. Scale bar: 15 µm. Images are representative of three independent experiments (n = 50 cells). **(E)** Schematic representation of the expression cassettes used for the RUSH assay. **(F)** Schematic view of the RUSH assay principle. Plasma membrane (PM), endoplasmic reticulum (ER), Golgi (G), nucleus (N). **(G)** Imaging of the synchronized transport of SPnA18Y-SBP-EGFP-PD-L1 and SPoptA18Y-SBP-EGFP-PD-L1 transiently expressed in HeLa cells. Streptavidin-KDEL was used as an ER hook. Release from the ER was induced by addition of biotin at 0 min (time scale shown as hr:min). Scale bar: 20 µm. All data are representative of three independent experiments with n = 10.

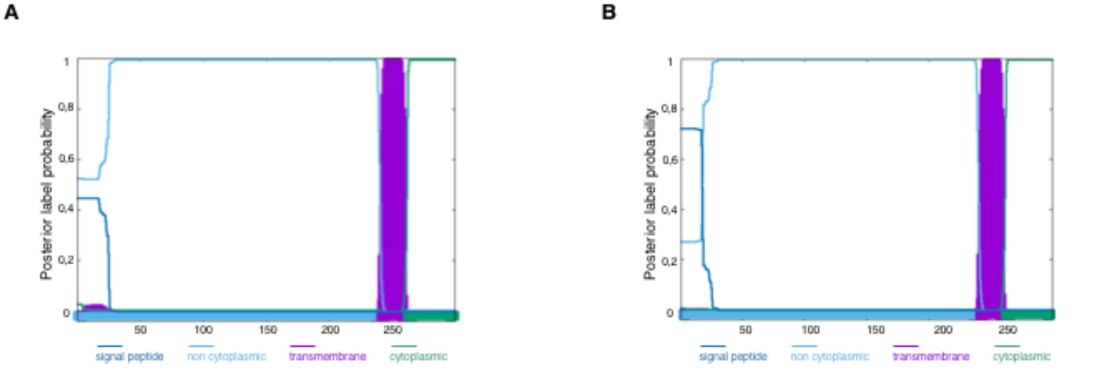

ID	SPn-A18Y-PDL1			
FT	Topo_dom	1	240	Non cytosolic
FT	Transmem	241	261	
FT	Topo_dom	262	299	Cytosolic

ID	SPopt-A18Y-PDL1			
FT	Topo_dom	1	240	Non cytosolic
FT	Transmem	241	261	
FT	Topo_dom	262	299	Cytosolic

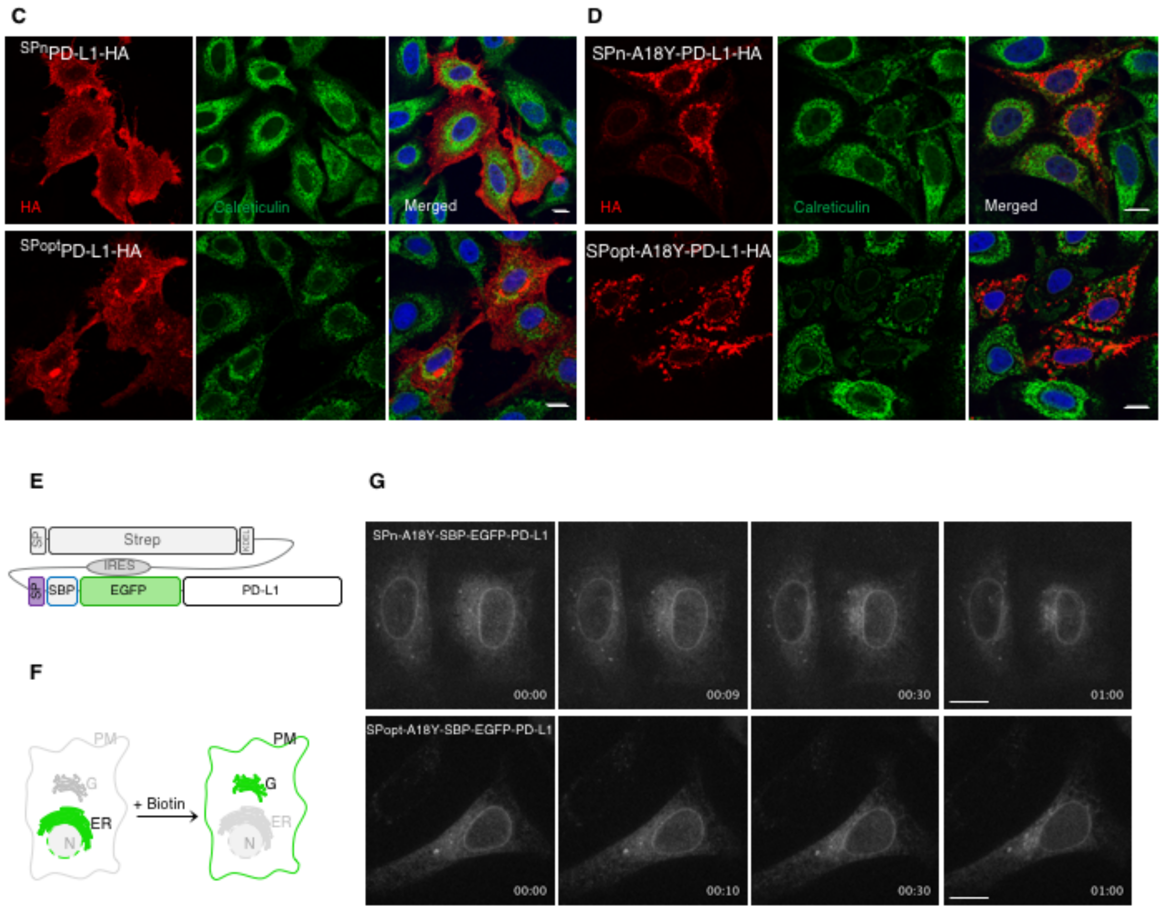

Fig. S10. Effect of SP optimization on PD-L1 glycosylation, maturation and cellular distribution.

(A, B) HeLa stably expressing SPn- and SPopt-PD-L1-HA were seeded on coverslips and fixed before being processed by indirect immunofluorescence with a specific antibody against HA tag and different intracellular markers calnexin (CLX, marker of the ER), TANGO1 (a marker of the ER-exit sites ERES), ERGIC-53 (a marker for ER-Golgi Intermediate Compartment ERGIC), GM130 (a marker of the cis-Golgi) and KDELr (a marker of the Golgi-ER retrograde transport). Magnifications are shown in dashed white squares on the right of each panel. Single focal sections are shown. Scale bar: 20 µm. **(C-F)** The histograms illustrate the colocalization, expressed as Manders coefficient M1, between PD-L1-HA variants and the above-mentioned intracellular markers. 20 randomly selected cells from each experiment were measured for co-localization. All data are mean ± SEM; statistical significance was determined by Student's t test. All data are representative of three independent experiments with n = 10. Levels of significance: **** P < 0.0001.

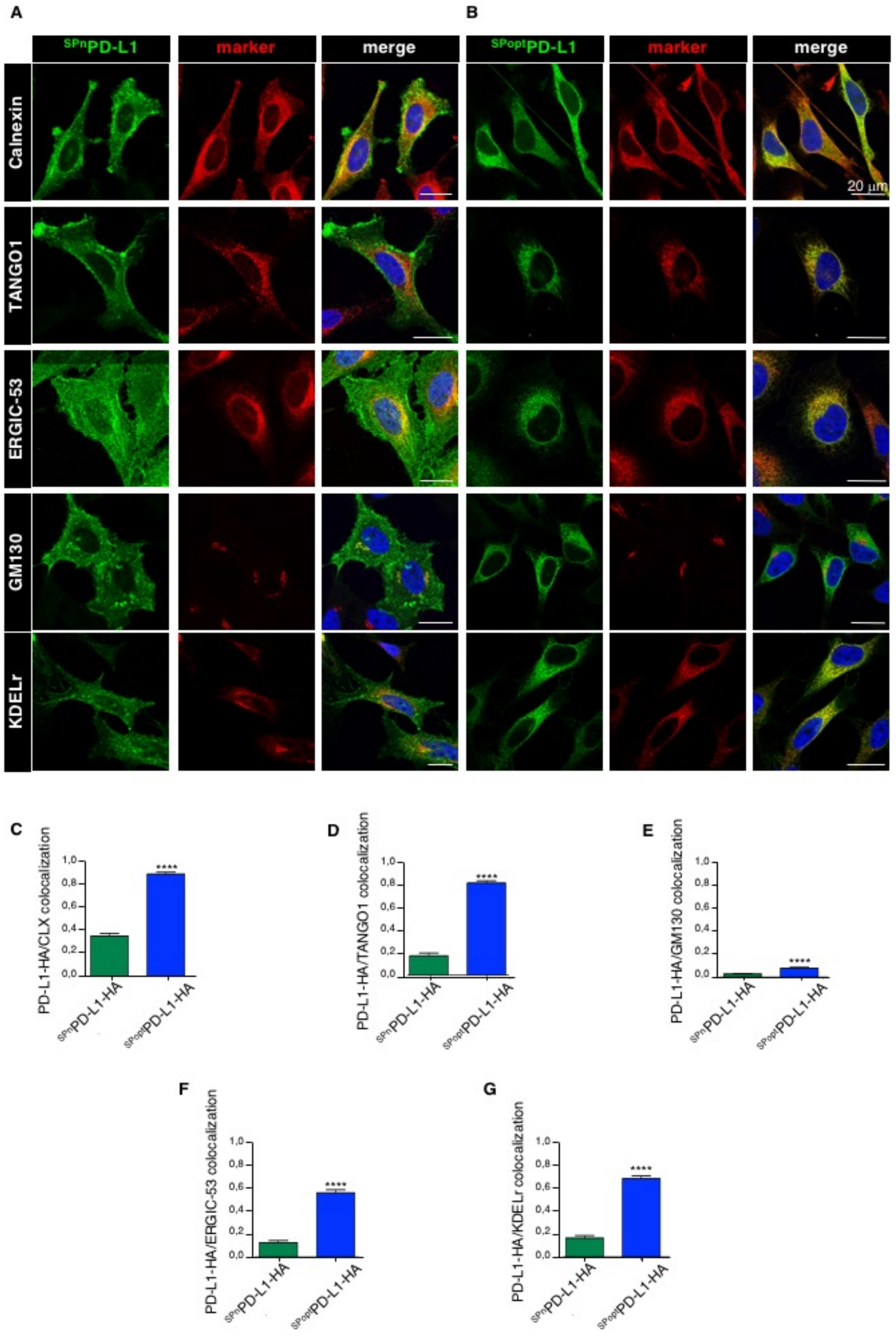
A
B
SPnPD-L1
marker
merge
SPoptPD-L1
marker
merge
Calnexin
TANGO1
ERGIC-53
GM130
KDELr
20 µm
C
PD-L1-HA/CLX colocalization
D
PD-L1-HA/TANGO1 colocalization
E
PD-L1-HA/GM130 colocalization
F
PD-L1-HA/ERGIC-53 colocalization
G
PD-L1-HA/KDELr colocalization
1,0
0,8
0,6
0,4
0,2
0,0
****
SPnPD-L1-HA
SPoptPD-L1-HA

Fig. S11. Effect of different SP on PD-L1 trafficking by RUSH assay.

(A) Synchronized transport of ^{SPn}-SBP-EGFP-PD-L1 and ^{SPopt}-SBP-EGFP-PD-L1 in HeLa cells. Streptavidin-KDEL was used as an ER hook. Trafficking was induced by the addition of biotin at 0 min (time scale shown as hr:min). Scale bar, 20 µm. (**B**) Representative curves of mean Golgi-region intensity change over time are displayed. Images were captured every 2 minutes and intensities were normalized to the maximal Golgi intensity with arbitrary units (a. u.). (^{SPn}-SBP-EGFP-PD-L1 n = 19 and ^{SPopt}-SBP-EGFP-PD-L1 = 21 independent cells ± SEM). **(C)** Comparison of ^{SPn}-SBP-EGFP-PD-L1 and ^{SPopt}-SBP-EGFP-PD-L1 fluorescence intensity in the Golgi with arbitrary units (arb. un.). All data are mean ± SEM; statistical significance was determined by Student's t test with data from three independent experiments with n = 10. Levels of significance: ** P < 0.01.

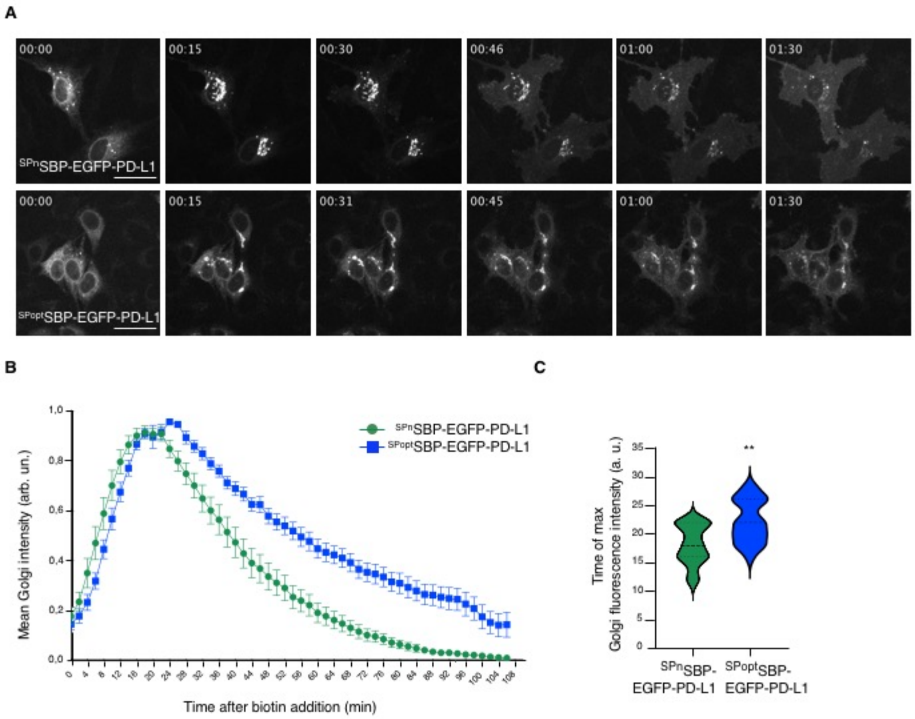

Table S1. Calculation of the hydrophobicity of the H-regions of PD-L1 signal peptides across different species.

The degree of hydrophobicity of the H-region of PD-L1 was determined from SPs belonging to 91 different PD-L1 species, using the Kyte-Doolittle scale. The mean values were normalized according to the different lengths of the H-regions of each SP.

Organism	Entry	SP Length	SP Sequence	H-Region Length	H-Region	H-Region Hydrophobicity
Otolemur garnettii (Small-eared galago) (Garnett's greater bushbaby)	H0WMG4	18	MKISNVFTFTAYWHLLNA	13	ISNVFTFTAYWHL	8,80
Microcebus murinus (Gray mouse lemur) (Lemur murinus)	A0A8C5VLG4	18	MRIFHVFTFTTYWHLLNA	13	IFHVFTFTTYWHL	10,20
Rousettus aegyptiacus (Egyptian rousette) (Egyptian fruit bat)	A0A7J8IG79	18	MRTFSGFTFMAYCHLLKA	13	TFSGFTFMAYCHL	11,30
Propithecus coquereli (Coquerel's sifaka) (Propithecus verreauxi coquereli)	A0A2K6F2X4	18	MKIFNVFTFTAYWHLLNA	13	IFNVFTFTAYWHL	12,40
Orycteropus afer afer	A0A8B7A983	18	MRIFNVFTFMTYSHLLNA	13	IFNVFTFMTYSHL	12,60
Prolemur simus (Greater bamboo lemur) (Hapalemur simus)	A0A8C9AQD7	18	MRIFDIFTFTAYWHLLNA	13	IFDIFTFTAYWHL	12,70
Loxodonta africana (African elephant)	G3STX2	18	MRILNVLTFMTYSHFLNA	13	ILNVLTFMTYSHF	13,60
Carlito syrichta (Philippine tarsier) (Tarsius syrichta)	A0A1U7TSZ1	18	MRILPVFTFTAYWHLLNA	13	ILPVFTFTAYWHL	15,30
Chrysochloris asiatica (Cape golden mole)	A0A6I9JQK5	18	MRIFNVLTFLTYSHLLNA	13	IFNVLTFLTYSHL	15,50
Rhinolophus ferrumequinum (Greater horseshoe bat)	A0A671F8A8	18	MRIFSGFTFMAYCHLLKA	13	IFSGFTFMAYCHL	16,50
Ornithorhynchus anatinus (Duckbill platypus)	F6TJX0	18	MKILPVFTFMLDWQLLNA	13	ILPVFTFMLDWQL	17,40
Pipistrellus kuhlii (Kuhl's pipistrelle)	A0A7J7W2C4	18	MRILTSFTLMAYCHLLKA	13	ILTSFTLMAYCHL	18,20
Suricata suricatta (Meerkat)	A0A673VEH7	18	MRIFSVFEFMAYCHLLKA	13	IFSVFEFMAYCHL	18,30
Canis lupus dingo (dingo)	A0A8C0JVJ1	18	MRMFSVFTFMAYCHLLKA	13	MFSVFTFMAYCHL	18,50
Canis lupus familiaris (Dog) (Canis familiaris)	E2RKZ5	18	MRMFSVFTFMAYCHLLKA	13	MFSVFTFMAYCHL	18,50
Myotis lucifugus (Little brown bat)	G1PE22	18	MRILGSFTLMAYCHLLKA	13	ILGSFTLMAYCHL	18,50
Myotis myotis (Greater mouse-eared bat)	A0A7J7UNA3	18	MRILGSFTLMAYCHLLKA	13	ILGSFTLMAYCHL	18,50
Vulpes vulpes (Red fox)	A0A3Q7SP91	18	MRMFSVFTFMAYCHLLKA	13	MFSVFTFMAYCHL	18,50
Nannospalax galili (Northern Israeli blind subterranean mole rat) (Spalax galili)	A0A8C6R534	18	MRIFNIFTFITYCHLLNA	13	IFNIFTFITYCHL	18,80
Oryctolagus cuniculus (Rabbit)	G1SUI3	18	MRIFNVFTFIAHCHLLNA	13	IFNVFTFIAHCHL	19,10
Tursiops truncatus (Atlantic bottle-nosed dolphin) (Delphinus truncatus)	A0A2U3V0G5	18	MRIYSIFTFMAYSCLLKA	13	IYSIFTFMAYSCL	19,70
Ictidomys tridecemlineatus (Thirteen-lined ground squirrel) (Spermophilus tridecemlineatus)	I3MQF2	18	MRMFNVFIFTSFCHLLNA	13	MFNVFIFTSFCHL	19,90
Marmota marmota marmota (Alpine marmot)	A0A8C6EMT0	18	MRMFNVFIFTSFCHLLNA	13	MFNVFIFTSFCHL	19,90
Marmota monax (Woodchuck)	F5A531	18	MRMFNVFIFTSFCHLLNA	13	MFNVFIFTSFCHL	19,90
Spermophilus dauricus (Daurian ground squirrel)	A0A8C9Q092	18	MRMFNVFIFTSFCHLLNA	13	MFNVFIFTSFCHL	19,90
Urocitellus parryii (Arctic ground squirrel) (Spermophilus parryii)	A0A8D2HNL7	18	MTMFNVFIFTSFCHLLNA	13	MFNVFIFTSFCHL	19,90
Gorilla gorilla gorilla (Western lowland gorilla)	G3QZN5	18	MRIFAVFIFTTYWHLLNA	13	IFAVFIFTTYWHL	20,40
Castor canadensis (American beaver)	A0A8B7U6V9	18	MRIFNAFVFTVYCHLLNA	13	IFNAFVFTVYCHL	20,70
Chinchilla lanigera (Long-tailed chinchilla) (Chinchilla villidera)	A0A8C2V5L3	18	MRILGIFIFTAYYDLLHA	13	ILGIFIFTAYYDL	21,30
Pongo abelii (Sumatran orangutan) (Pongo pygmaeus abelii)	H2PS75	18	MRIFAVLIFTTYWHLLNA	13	IFAVLIFTTYWHL	21,40
Neovison vison (American mink) (Mustela vison)	U6CTF8	18	MRIFSVFAFMAHCHLLKA	13	IFSVFAFMAHCHL	21,70
Equus asinus asinus	A0A8C4LWJ8	18	MRIVSVFTFMAYCHLLKA	13	IVSVFTFMAYCHL	22,50
Equus caballus (Horse)	F7DZ76	18	MRIVSVFTFMAYCHLLKA	13	IVSVFTFMAYCHL	22,50
Vicugna pacos (Alpaca) (Lama pacos)	A0A6J0AR17	18	MRIYSVFTFMAYCCLLKA	13	IYSVFTFMAYCCL	22,70
Aotus nancymaae (Ma's night monkey)	A0A2K5DL24	18	MRIFAVFIFMTYWHLLNA	13	IFAVFIFMTYWHL	23,00
Balaenoptera acutorostrata scammoni (North Pacific minke whale) (Balaenoptera davidsoni)	A0A384A4I4	18	MRIYSIFTFMAYCCLLKA	13	IYSIFTFMAYCCL	23,00
Balaenoptera musculus (Blue whale)	A0A8B8XZR8	18	MRIYSIFTFMAYCCLLKA	13	IYSIFTFMAYCCL	23,00
Callithrix jacchus (White-tufted-ear marmoset)	F7GIW2	18	MRIFAVFIFMTYWHLLNA	13	IFAVFIFMTYWHL	23,00
Cebus imitator (Panamanian white-faced capuchin) (Cebus capucinus imitator)	A0A2K5PDS0	18	MRIFAVFIFMTYWHLLNA	13	IFAVFIFMTYWHL	23,00
Homo sapiens (Human)	Q9NZQ7	18	MRIFAVFIFMTYWHLLNA	13	IFAVFIFMTYWHL	23,00
Lipotes vexillifer (Yangtze river dolphin)	A0A340X5J4	18	MRIYSIFTFMAYCCLLKA	13	IYSIFTFMAYCCL	23,00
Monodon monoceros (Narwhal) (Ceratodon monodon)	A0A8C6FDS8	18	MRIYSIFTFMAYCCLLKA	13	IYSIFTFMAYCCL	23,00
Nomascus leucogenys (Northern white-cheeked gibbon) (Hylobates leucogenys)	G1RCP1	18	MRIFAVFIFMTYWHLLNA	13	IFAVFIFMTYWHL	23,00
Pan paniscus (Pygmy chimpanzee) (Bonobo)	A0A2R9B063	18	MRIFAVFIFMTYWHLLNA	13	IFAVFIFMTYWHL	23,00
Pan troglodytes (Chimpanzee)	H2QWZ8	18	MRIFAVFIFMTYWHLLNA	13	IFAVFIFMTYWHL	23,00
Phocoena sinus (Vaquita)	A0A8C9D0L1	18	MRIYSIFTFMAYCCLLKA	13	IYSIFTFMAYCCL	23,00
Physeter macrocephalus (Sperm whale) (Physeter catodon)	A0A2Y9SVI0	18	MRIYSIFTFMAYCCLLKA	13	IYSIFTFMAYCCL	23,00
Saimiri boliviensis boliviensis (Bolivian squirrel monkey)	A0A2K6SKE1	18	MRIFAVFIFMTYWHLLNA	13	IFAVFIFMTYWHL	23,00
Odobenus rosmarus divergens (Pacific walrus)	A0A2U3ZNI8	18	MKIFSLFAFMAYCHLLKA	13	IFSLFAFMAYCHL	23,20
Acinonyx jubatus (Cheetah)	A0A6I9ZHR3	18	MRIFSVFAFMAYCHLLKA	13	IFSVFAFMAYCHL	23,60
Felis catus (Cat) (Felis silvestris catus)	A0A5F5XRI1	18	MRIFSVFAFMAYCHLLKA	13	IFSVFAFMAYCHL	23,60
Lynx canadensis (Canada lynx)	A0A667HJA0	18	MRIFSVFAFMAYCHLLKA	13	IFSVFAFMAYCHL	23,60
Mustela putorius furo (European domestic ferret) (Mustela furo)	M3Y0R4	18	MRIFSVFAFMAHCYLLKA	13	IFSVFAFMAHCYL	23,60
Panthera leo (Lion)	A0A8C8XWQ3	18	MRIFSVFAFMAYCHLLKA	13	IFSVFAFMAYCHL	23,60
Panthera pardus (Leopard) (Felis pardus)	A0A6P4XL56	18	MRIFSVFAFMAYCHLLKA	13	IFSVFAFMAYCHL	23,60
Panthera tigris altaica (Siberian tiger)	A0A8C9K110	18	MRIFSVFAFMAYCHLLKA	13	IFSVFAFMAYCHL	23,60
Puma concolor (Mountain lion) (Felis concolor)	A0A6P6IH14	18	MRIFSVFAFMAYCHLLKA	13	IFSVFAFMAYCHL	23,60
Ursus maritimus (Polar bear) (Thalarctos maritimus)	A0A384C2Q2	18	MKIFSVFAFMAYCHLLKA	13	IFSVFAFMAYCHL	23,60
Bos mutus grunniens (Wild yak) (Bos grunniens)	A0A1Y1C055	18	MRIYSVLTFMAYCCLLKA	13	IYSVLTFMAYCCL	23,70
Bos taurus (Bovine)	C5NU11	18	MRIYSVLTFMAYCCLLKA	13	IYSVLTFMAYCCL	23,70
Capra hircus (Goat)	A0A452F002	18	MRIYSVLTFMAYCCLLKA	13	IYSVLTFMAYCCL	23,70
Moschus moschiferus (Siberian musk deer) (Moschus sibiricus)	A0A8C6CQF5	18	MRIYSVLTFMAYCCLLKA	13	IYSVLTFMAYCCL	23,70
Ovis aries (Sheep)	W5PVJ5	18	MRIYSVLTFMAYCCLLKA	13	IYSVLTFMAYCCL	23,70
Neomonachus schauinslandi (Hawaiian monk seal) (Monachus schauinslandi)	A0A2Y9HEA8	18	MKIFSIFAFMAYCHLLKA	13	IFSIFAFMAYCHL	23,90
Phyllostomus discolor (pale spear-nosed bat)	A0A7E6DEY4	18	MRILSSAILMAYCYLLKA	13	ILSSAILMAYCYL	24,20
Sarcophilus harrisii (Tasmanian devil) (Sarcophilus laniarius)	A0A7N4P6H3	19	MIWILLVLAFMFSWHLLNA	14	WILLVLAFMFSWHL	27,40
Cercocebus atys (Sooty mangabey) (Cercocebus torquatus atys)	A0A2K5P1K8	18	MRIFAVFIFTIYWHLLNA	13	IFAVFIFTIYWHL	25,60
Chlorocebus sabaeus (Green monkey) (Cercopithecus sabaeus)	A0A0D9R951	18	MRIFAVFIFTIYWHLLNA	13	IFAVFIFTIYWHL	25,60
Colobus angolensis palliatus (Peters' Angolan colobus)	A0A2K5JDJ4	18	MRIFAVFIFTIYWHLLNA	13	IFAVFIFTIYWHL	25,60
Macaca fascicularis (Crab-eating macaque) (Cynomolgus monkey)	G7PSE7	18	MRIFAVFIFTIYWHLLNA	13	IFAVFIFTIYWHL	25,60
Macaca mulatta (Rhesus macaque)	F6VEW6	18	MRIFAVFIFTIYWHLLNA	13	IFAVFIFTIYWHL	25,60
Macaca nemestrina (Pig-tailed macaque)	A0A2K6E3F1	18	MRIFAVFIFTIYWHLLNA	13	IFAVFIFTIYWHL	25,60
Mandrillus leucophaeus (Drill) (Papio leucophaeus)	A0A2K5YFJ3	18	MRIFAVFIFTIYWHLLNA	13	IFAVFIFTIYWHL	25,60
Papio anubis (Olive baboon)	A0A096P1L0	18	MRIFAVFIFTIYWHLLNA	13	IFAVFIFTIYWHL	25,60
Piliocolobus tephrosceles (Ugandan red Colobus)	A0A8C9GJZ8	18	MRIFAVFIFTIYWHLLHA	13	IFAVFIFTIYWHL	25,60
Rhinopithecus roxellana (Golden snub-nosed monkey) (Pygathrix roxellana)	A0A2K6PJ04	18	MRIFAVFIFTIYWHLLNA	13	IFAVFIFTIYWHL	25,60
Theropithecus gelada (Gelada baboon)	A0A8D2FM35	18	MRIFAVFIFTIYWHLLNA	13	IFAVFIFTIYWHL	25,60
Peromyscus maniculatus bairdii (Prairie deer mouse)	A0A8C8U5P5	18	MRTSIIVTFTVCCGLLNA	13	TSIIVTFTVCCGL	25,70
Ailuropoda melanoleuca (Giant panda)	G1LTC6	18	MKIFSVFAFMVYCHLLKA	13	IFSVFAFMVYCHL	26,00
Sus scrofa (Pig)	Q4QTK1	18	MRICSIFTFMAYCCLLEA	13	ICSIFTFMAYCCL	26,80
Sus scrofa domesticus (domestic pig)	A0A0B8RT47	18	MRICSIFTFMAYCCLLEA	13	ICSIFTFMAYCCL	26,80
Vombatus ursinus (Common wombat)	A0A4X2KHJ2	19	MGISLVFVFMFSWHLLINA	14	ISLVFVFMFSWHLL	28,90
Mus musculus (Mouse)	Q9EP73	18	MRIFAGIIFTACCHLLRA	13	IFAGIIFTACCHL	27,20
Sciurus vulgaris (Eurasian red squirrel)	A0A8D2DYS3	18	MRIFNVCIFISFCHLLNA	13	IFNVCIFISFCHL	27,40
Rattus norvegicus (Rat)	D4AE25	17	MRIFAVLIVTACSHVLA	12	IFAVLIVTACSH	25,40
Cavia porcellus (Guinea pig)	H0V301	18	MRIFAIFIFTFCYHLLHA	13	IFAIFIFTFCYHL	27,60
Catagonus wagneri (Chacoan peccary)	A0A8C3YT23	18	MRIFSIFAFMAYCCLLEA	13	IFSIFAFMAYCCL	29,60
Mus spicilegus (Steppe mouse)	A0A8C6N227	18	MRIFAVIIFTACCHLLQA	13	IFAVIIFTACCHL	31,80
Mus spretus (Western Mediterranean mouse) (Algerian mouse)	E2D891	18	MRIFAVIIFTACCHLLQA	13	IFAVIIFTACCHL	31,80
Cricetulus griseus (Chinese hamster) (Cricetulus barabensis griseus)	A0A8C2QPR3	19	MRVCAIVAIFTVCCHLVNA	14	VCAIVAIFTVCCHL	35,40
Mus caroli (Ryukyu mouse) (Ricefield mouse)	A0A6P5P6X5	18	MRIFAVIIVTACCHLLRA	13	IFAVIIVTACCHL	33,20

Table S2A and B. Calculation of the hydrophobicity of the H-regions of human model signal peptides.

The degree of hydrophobicity of the H-regions of human model SPs was determined for 100 randomly selected human secreted proteins and 100 randomly selected human transmembrane proteins using the Kyte-Doolittle scale. The mean values were normalized according to the different lengths of the H-regions of each SP.

Table S2A

Entry	Entry Name	Gene Names	Protein Len	SP Length	SP sequence	H-Region L	H-Region	Hydrophob	norm
O60667	FCMR_HUMAN	FCMR	390	16	MDFWLWPLYFLPVSGA	10	FWLWPLYFLP	10,7	1,07
P11464	PSG1_HUMAN	PSG1	419	34	MGTLSAPPCTQRIKWKGLLLTASLLNFWNLPTTA	18	IKWKGLLLTASLLNFWNL	13,4	0,74
P13591	NCAM1_HUMAN	NCAM1	858	19	MLQTKDLIWTLFFLGTAVS	10	DLIWTLFFLG	16	1,6
P21860	ERBB3_HUMAN	ERBB3	1342	19	MRANDALQVLGLLFSLARG	13	NDALQVLGLLFSL	16,1	1,24
O75339	CILP1_HUMAN	CILP	1184	21	MVGTKAWVFSFLVLEVTSVLG	14	TKAWVFSFLVLEVT	17,1	1,22
P13688	CEAM1_HUMAN	CEACAM1	526	34	MGHLSAPLHRVRVPWQGLLLTASLLTFWNPPTTA	25	LSAPLHRVRVPWQGLLLTASLLTFW	17,3	0,69
P04180	LCAT_HUMAN	LCAT	440	24	MGPPGSPWQWVTLLLGLLLPPAAP	14	PWQWVTLLLGLLLP	17,4	1,24
P25942	TNR5_HUMAN	CD40 TNFRSF5_iso2	277	20	MVRLPLQCVLWGCLLTAVHP	9	VLWGCLLTA	17,90	1,99
O60469	DSCAM_HUMAN	DSCAM	2012	17	MWILALSLFQSFANVFS	10	ILALSLFQSF	18,2	1,82
O14960	LECT2_HUMAN	LECT2	151	18	MFSTKALLLAGLISTALA	10	TKALLLAGLI	18,3	1,83
Q6UW88	EPGN_HUMAN	EPGN UNQ3072/PRO9904	154	22	MALGVPISVYLLFNAMTALTEE	11	VYLLFNAMTAL	18,40	1,67
P02679	FIBG_HUMAN	FGG	453	26	MSWSLHPRNLILYFYALLFLSSTCVA	17	LHPRNLILYFYALLFLS	18,5	1,09
Q9Y6W8	ICOS_HUMAN	ICOS AILIM_iso2	199	20	MKSGLWYFFLFCLRIKVLTG	8	FFLFCLRI	18,50	2,31
O60687	SRPX2_HUMAN	SRPX2	465	23	MASQLTQRGALFLLFFLTPAVTP	14	LTQRGALFLLFFLT	19,4	1,39
O60687	SRPX2_HUMAN	SRPX2	465	23	MASQLTQRGALFLLFFLTPAVTP	14	LTQRGALFLLFFLT	19,4	1,39
O60568	PLOD3_HUMAN	PLOD3	738	24	MTSSGPGPRFLLLLPLLLPPAASA	13	GPRFLLLLPLLLP	19,7	1,52
O75594	PGRP1_HUMAN	PGLYRP1	196	21	MSRRSMLLAWALPSLLRLGAA	14	SMLLAWALPSLLRL	19,7	1,41
P21583	SCF_HUMAN	KITLG	273	25	MKKTQTWILTCIYLQLLLFNPLVKT	19	TQTWILTCIYLQLLLFNPL	20,7	1,09
P08572	CO4A2_HUMAN	COL4A2	1712	33	MGRDQRAVAGPALRRWLLLGTVTVGFLAQSVLA	14	WLLLGTVTVGFLAQ	21,6	1,54
P20851	C4BPB_HUMAN	C4BPB	252	17	MFFWCACCLMVAWRVSA	11	FWCACCLMVAW	22	2
P02743	SAMP_HUMAN	APCS	223	19	MNKPLLWISVLTSLLEAFA	12	PLLWISVLTSLL	22,9	1,91
P09544	WNT2_HUMAN	WNT2	360	25	MNAPLGGIWLWLPLLLTWLTPEVNS	17	PLGGIWLWLPLLLTWLT	23	1,35
P20061	TCO1_HUMAN	TCN1	433	23	MRQSHQLPLVGLLLFSFIPSQLC	15	SHQLPLVGLLLFSFI	23	1,53
O43155	FLRT2_HUMAN	FLRT2	660	35	MGLQTTKWPSHGAFFLKSWLIISLGLYSQVSKLLA	16	GAFFLKSWLIISLGLY	23,1	1,44
P18510	IL1RA_HUMAN	IL1RN	177	25	MEICRGLRSHLITLLLFLFHSETIC	14	GLRSHLITLLLFLF	23,3	1,66
O75610	LFTY1_HUMAN	LEFTY1	366	21	MQPLWLCWALWVLPLASPGAA	14	PLWLCWALWVLPLA	23,4	1,67
P05451	REG1A_HUMAN	REG1A	166	22	MAQTSSYFMLISCLMFLSQSQG	14	TSSYFMLISCLMFL	23,4	1,67
P01615	KVD28_HUMAN	IGKV2D-28	120	20	MRLPAQLLGLLMLWVSGSSG	13	LPAQLLGLLMLWV	24,3	1,87
P02771	FETA_HUMAN	AFP	609	18	MKWVESIFLIFLLNFTES	13	WVESIFLIFLLNF	24,3	1,87
P09683	SECR_HUMAN	SCT	121	18	MAPRPLLLLLLLLGGSAA	10	RPLLLLLLLL	24,3	2,43
P10646	TFPI1_HUMAN	TFPI	304	28	MIYTMKKVHALWASVCLLLNLAPAPLNA	14	VHALWASVCLLLNL	25,1	1,79
P18075	BMP7_HUMAN	BMP7	431	29	MHVRSLRAAAPHSFVALWAPLFLLRSALA	19	LRAAAPHSFVALWAPLFLL	25,2	1,33
P21815	SIAL_HUMAN	IBSP	317	16	MKTALILLSILGMACA	11	TALILLSILGM	26	2,36
P01185	NEU2_HUMAN	AVP	164	19	MPDTMLPACFLGLLAFSSA	13	TMLPACFLGLLAF	26,1	2,01
P01597	KV139_HUMAN	IGKV1-39	117	22	MDMRVPAQLLGLLLLWLRGARC	13	VPAQLLGLLLLWL	26,2	2,02
P08253	MMP2_HUMAN	MMP2	660	29	MEALMARGALTGPLRALCLLGCLLSHAAA	18	GALTGPLRALCLLGCLLS	26,4	1,47
P19021	AMD_HUMAN	PAM	973	20	MAGRVPSLLVLLVFPSSCLA	11	VPSLLVLLVFP	26,6	2,42
O43854	EDIL3_HUMAN	EDIL3	480	23	MKRSVAVWLLVGLSLGVPQFGKG	12	SVAVWLLVGLSL	26,7	2,23
O75443	TECTA_HUMAN	TECTA	2155	24	MNYSSFLRIWVSFIFALVQHQAQP	15	SSFLRIWVSFIFALV	27,4	1,83
P13671	CO6_HUMAN	C6	934	21	MARRSVLYFILLNALINKGQA	12	SVLYFILLNALI	27,4	2,28
O00622	CCN1_HUMAN	CCN1	381	24	MSSRIARALALVVTLLHLTRLALS	17	IARALALVVTLLHLTRL	27,5	1,62
O43897	TLL1_HUMAN	TLL1	1013	30	MGLGTLSPRMLVWLVASGIVFYGELWVCAG	16	MLVWLVASGIVFYGEL	27,7	1,73
P03956	MMP1_HUMAN	MMP1	469	19	MHSFPPLLLLLFWGVVSHS	14	SFPPLLLLLFWGVV	27,7	1,98
P01308	INS_HUMAN	INS	110	24	MALWMRLLPLLALLALWGPDPAAA	15	LWMRLLPLLALLALW	28	1,87
P01579	IFNG_HUMAN	IFNG	166	23	MKYTSYILAFQLCIVLGSLGCYC	16	TSYILAFQLCIVLGSL	28	1,75
P10909	CLUS_HUMAN	CLU	449	22	MMKTLLLFVGLLLTWESGQVLG	11	TLLLFVGLLLT	28	2,55
P10645	CMGA_HUMAN	CHGA	457	18	MRSAAVLALLLCAGQVTA	11	SAAVLALLLCA	28,3	2,57
P05997	CO5A2_HUMAN	COL5A2	1499	26	MMANWAEARPLLILIVLLGQFVSIKA	18	WAEARPLLILIVLLGQFV	28,4	1,58
O15232	MATN3_HUMAN	MATN3	486	28	MPRPAPARRLPGLLLLLWPLLLLPSAAP	15	RLPGLLLLLWPLLLL	29	1,93
B2RUY7	VWC2L_HUMAN	VWC2L	222	21	MALHIHEACILLLVIPGLVTS	16	LHIHEACILLLVIPGL	29,1	1,82
P19835	CEL_HUMAN	CEL	753	20	MGRLQLVVLGLTCCWAVASA	14	LQLVVLGLTCCWAV	29,1	2,08
O60938	KERA_HUMAN	KERA	352	20	MAGTICFIMWVLFITDTVWS	12	TICFIMWVLFIT	29,2	2,43
P01764	HV323_HUMAN	IGHV3-23	117	19	MEFGLSWLFLVAILKGVQC	12	FGLSWLFLVAIL	29,2	2,43
P15941	MUC1_HUMAN	MUC1	1255	23	MTPGTQSPFFLLLLLTVLTVVTG	15	QSPFFLLLLLTVLTV	29,5	1,97
P05452	TETN_HUMAN	CLEC3B	202	21	MELWGAYLLLCLFSLLTQVTT	15	LWGAYLLLCLFSLLT	29,6	1,97
P15814	IGLL1_HUMAN	IGLL1	213	44	MRPGTGQGGLEAPGEPGPNLRQRWPLLLLGLAVVTHGLLRPTAA	16	WPLLLLGLAVVTHGLL	29,6	1,85
P08700	IL3_HUMAN	IL3	152	19	MSRLPVLLLLQLLVRPGLQ	11	LPVLLLLQLLV	29,9	2,72
P04090	REL2_HUMAN	RLN2	185	24	MPRLFFFHLLGVCLLLNQFSRAVA	16	LFFFHLLGVCLLLNQF	30,1	1,88
P01571	IFN17_HUMAN	IFNA17	189	23	MALSFSLLMAVLVLSYKSICSLG	14	LSFSLLMAVLVLSY	30,2	2,16
P02649	APOE_HUMAN	APOE	317	18	MKVLWAALLVTFLAGCQA	12	VLWAALLVTFLA	30,2	2,52
P07942	LAMB1_HUMAN	LAMB1	1786	21	MGLLQLLAFSFLALCRARVRA	13	LLQLLAFSFLALC	30,2	2,32
P02745	C1QA_HUMAN	C1QA	245	20	MEGPRGWLVLCVLAISLASM	12	GWLVLCVLAISL	30,3	2,52
P08637	FCG3A_HUMAN	FCGR3A	254	20	MWQLLLPTALLLLVSAGMRT	11	LLLPTALLLLV	30,3	2,75
O43866	CD5L_HUMAN	CD5L	347	19	MALLFSLILAICTRPGFLA	10	LLFSLILAIC	30,5	3,05
P08887	IL6RA_HUMAN	IL6R	468	19	MLAVGCALLAALLAAPGAA	12	AVGCALLAALLA	30,5	2,54
P09603	CSF1_HUMAN	CSF1	554	32	MTAPGAAGRCPPTTWLGSLLLLVCLLASRSIT	18	CPPTTWLGSLLLLVCLLA	30,9	1,72
P01011	AACT_HUMAN	SERPINA3	423	23	MERMLPLLALGLLAAGFCPAVLC	14	LPLLALGLLAAGFC	31,1	2,22
P01275	GLUC_HUMAN	GCG	180	20	MKSIYFVAGLFVMLVQGSWQ	13	SIYFVAGLFVMLV	31,5	2,42
P07492	GRP_HUMAN	GRP	148	23	MRGRELPLVLLALVLCLAPRGRA	15	RELPLVLLALVLCLA	31,5	2,1
P02748	CO9_HUMAN	C9	559	26	MSACRSFAVAICILEISILTAQYTTS	16	CRSFAVAICILEISIL	31,6	1,98
P16112	PGCA_HUMAN	ACAN	2530	22	MTTLLWVFVTLRVITAAVTVET	15	LLWVFVTLRVITAAV	32,3	2,15
O76093	FGF18_HUMAN	FGF18	207	27	MYSAPSACTCLCLHFLLLCFQVQVLVA	17	SACTCLCLHFLLLCFQV	32,4	1,91
O95750	FGF19_HUMAN	FGF19	216	22	MRSGCVVVHVWILAGLWLAVAG	16	GCVVVHVWILAGLWLA	33	2,06
P09466	PAEP_HUMAN	PAEP	180	18	MLCLLLTLGVALVCGVPA	12	CLLLTLGVALVC	33,1	2,76
P16870	CBPE_HUMAN	CPE	476	27	MAGRGGSALLALCGALAACGWLLGAEA	19	GGSALLALCGALAACGWLL	33,5	1,76
P19875	CXCL2_HUMAN	CXCL2	107	34	MARATLSAAPSNPRLLRVALLLLLLVAASRRAAG	24	TLSAAPSNPRLLRVALLLLLLVAA	33,6	1,4
O00300	TR11B_HUMAN	TNFRSF11B	401	21	MNNLLCCALVFLDISIKWTTQ	13	LLCCALVFLDISI	33,7	2,59
P04275	VWF_HUMAN	VWF	2813	22	MIPARFAGVLLALALILPGTLC	13	FAGVLLALALILP	33,9	2,61
P02452	CO1A1_HUMAN	COL1A1	1464	22	MFSFVDLRLLLLLAATALLTHG	16	FVDLRLLLLLAATALL	34,1	2,13
P02747	C1QC_HUMAN	C1QC	245	28	MDVGPSSLPHLGLKLLLLLLLLPLRGQA	18	SLPHLGLKLLLLLLLLPL	34,1	1,89
P02774	VTDB_HUMAN	GC	474	16	MKRVLVLLLAVAFGHA	10	VLVLLLAVAF	34,2	3,42
P05814	CASB_HUMAN	CSN2	226	15	MKVLILACLVALALA	10	VLILACLVAL	34,2	3,42
P05814	CASB_HUMAN	CSN2	226	15	MKVLILACLVALALA	10	VLILACLVAL	34,2	3,42
O60882	MMP20_HUMAN	MMP20	483	29	MKVLPASGLAVFLIMALKFSTAAPSLVAA	17	VLPASGLAVFLIMALKF	34,3	2,02
P01732	CD8A_HUMAN	CD8A	235	21	MALPVTALLLPLALLLHAARP	14	LPVTALLLPLALLL	34,3	2,45
P02812	PRB2_HUMAN	PRB2	416	16	MLLILLSVALLALSSA	11	LILLSVALLAL	34,3	3,12
O15230	LAMA5_HUMAN	LAMA5	3695	35	MAKRLCAGSALCVRGPRGPAPLLLVGLALLGAARA	26	LCAGSALCVRGPRGPAPLLLVGLALL	34,8	1,34
P04155	TFF1_HUMAN	TFF1	84	26	MATMENKVICALVLVSMLALGTLAEA	15	KVICALVLVSMLALG	35,2	2,35
P21128	ENDOU_HUMAN	ENDOU	410	18	MRACISLVLAVLCGLAWA	13	ACISLVLAVLCGL	35,5	2,73
P01270	PTHY_HUMAN	PTH	115	25	MIPAKDMAKVMIVMLAICFLTKSDG	11	VMIVMLAICFL	35,9	3,26
P13284	GILT_HUMAN	IFI30	250	26	MTLSPLLLFLPPLLLLLDVPTAAVQA	17	LSPLLLFLPPLLLLLDV	35,9	2,11
O75629	CREG1_HUMAN	CREG1	220	31	MAGLSRGSARALLAALLASTLLALLVSPARG	23	LSRGSARALLAALLASTLLALLV	36,7	1,6
P08620	FGF4_HUMAN	FGF4	206	30	MSGPGTAAVALLPAVLLALLAPWAGRGGAA	19	GTAAVALLPAVLLALLAPW	36,8	1,94
O43323	DHH_HUMAN	DHH	396	22	MALLTNLLPLCCLALLALPAQS	16	LLTNLLPLCCLALLAL	37	2,31
O95393	BMP10_HUMAN	BMP10	424	21	MGSLVLTLCALFCLAAYLVSG	15	LVLTLCALFCLAAYL	38,2	2,55
P19438	TNR1A_HUMAN	TNFRSF1A	455	29	MGLSTVPDLLLPLVLLELLVGIYPSGVIG	20	LSTVPDLLLPLVLLELLVGI	39,2	1,96
O15520	FGF10_HUMAN	FGF10	208	39	MWKWILTHCASAFPHLPGCCCCCFLLLFLVSSVPVTCQA	21	FPHLPGCCCCCFLLLFLVSSV	39,9	1,9
O15263	DFB4A_HUMAN	DEFB4A	64	23	MRVLYLLFSFLFIFLMPLPGVFG	16	VLYLLFSFLFIFLMPL	40,9	2,56
P02776	PLF4_HUMAN	PF4	101	31	MSSAAGFCASRPGLLFLGLLLLPLVVAFASA	17	PGLLFLGLLLLPLVVAF	42,2	2,48
P07998	RNAS1_HUMAN	RNASE1	156	28	MALEKSLVRLLLLVLILLVLGWVQPSLG	18	SLVRLLLLVLILLVLGWV	48,9	2,72

Table S2B

Entry	Entry Name	Gene Names	Protein Length	SP Length	SP sequence	H-Region Length	H-Region	Hydrophobicity	norm
O60486	PLXC1_HUMAN	PLXNC1 VESPR	1568	34	MEVSRRKAPPRPPRPAAPLPLLAYLLALAAPGRG	27	VSRRKAPPRPPRPAAPLPLLAYLLALA	2,60	0,10
Q9ULB4	CADH9_HUMAN	CDH9	789	21	MRTYHYIPLFIWTYMFHTVDT	8	LFIWTYMF	12,90	1,61
P24046	GBRR1_HUMAN	GABRR1	479	21	MLAVPNMRFGIFLLWWGWVLA	10	FGIFLLWWGW	14,20	1,42
Q86WC4	OSTM1_HUMAN	OSTM1 GL HSPC019 UNQ6098/PRO21201	334	31	MEPGPTAAQRRCSLPPWLPLGLLLWSGLALG	9	WLPLGLLLW	15,20	1,69
Q7Z443	PK1L3_HUMAN	PKD1L3	1732	23	MFFKGGSWLWLYIRTSIILGSEL	12	WLWLYIRTSIIL	15,80	1,32
Q8TE23	TS1R2_HUMAN	TAS1R2	839	21	MGPRAKTISSLFFLLWVLAEP	12	AKTISSLFFLLW	16,2	1,35
Q15116	PDCD1_HUMAN	PDCD1 PD1	288	23	MQIPQAPWPVVWAVLQLGWRPGW	10	VVWAVLQLGW	16,30	1,63
P16070	CD44_HUMAN	CD44	742	20	MDKFWWHAAWGLCLVPLSLA	14	FWWHAAWGLCLVPL	16,60	1,19
P10966	CD8B_HUMAN	CD8B CD8B1_iso1	210	18	MRPRLWLLLAAQLTVLHG	12	RLWLLLAAQLTV	17,20	1,43
Q17R55	F187B_HUMAN	FAM187B	369	17	MPPMLWLLLHFAAPALG	9	MLWLLLHFA	17,6	1,96
Q9HCU0	CD248_HUMAN	CD248 CD164L1 TEM1	757	17	MLLRLLLAWAAAGPTLG	8	LLLAWAAA	17,70	2,21
P33681	CD80_HUMAN	CD80	288	34	MGHTRRQGTSPSKCPYLNFFQLLVLAGLSHFCSG	15	YLNFFQLLVLAGLSH	17,9	1,19
P13765	DOB_HUMAN	HLA-DOB	273	24	MGSGWVPWVVALLVNLTRLDSSMT	14	WVPWVVALLVNLTR	17,9	1,28
P13762	DRB4_HUMAN	HLA-DRB4	266	29	MVCLKLPGGSCMAALTVTLTVLSSPLALA	9	ALTVTLTVL	17,9	1,63
O15389	SIGL5_HUMAN	SIGLEC5 CD33L2 OBBP2	551	16	MLPLLLLPLLWGGSLQ	9	PLLLLPLLW	18,70	2,08
Q86V40	TIKI1_HUMAN	TRABD2A C2orf89 TIKI1	505	19	MSPWSWFLLQTLCLLPTGA	10	WFLLQTLCLL	19,20	1,92
Q9UJQ1	LAMP5_HUMAN	LAMP5	280	29	MDLQGRGVPSIDRLRVLLMLFHTMAQIMA	12	LRVLLMLFHTMA	19,40	1,62
Q9UIB8	SLAF5_HUMAN	CD84 SLAMF5	345	21	MAQHHLWILLLCLQTWPEAAG	11	LWILLLCLQTW	20,00	1,82
P49190	PTH2R_HUMAN	PTH2R PTHR2	550	24	MAGLGASLHVWGWLMLGSCLLARA	12	VWGWLMLGSCLL	20,40	1,70
P06280	AGAL_HUMAN	GLA	429	31	MQLRNPELHGCALALRFLALVSWDIPGARA	18	ELHGCALALRFLALVSW	20,60	1,14
P30532	ACHA5_HUMAN	CHRNA5 NACHRA5	468	22	MAARGSGPRALRLLLLVQLVAG	10	ALRLLLLVQL	20,80	2,08
O60939	SCN2B_HUMAN	SCN2B UNQ326/PRO386	215	29	MHRDAWLPRPAFSLTGLSLFFSLVPPGRS	21	AWLPRPAFSLTGLSLFFSLVP	21,50	1,02
Q92956	TNR14_HUMAN	TNFRSF14 HVEA HVEM UNQ329/PRO509	283	38	MEPPGDWGPPPWRSTPKTDVLRLVLYLTFLGAPCYAPA	10	LVLYLTFLGA	21,60	2,16
Q96CP7	TLCD1_HUMAN	TLCD1	247	35	MPRLLHPALPLLLGATLTFRALRRALCRLPLPVHV	12	ALPLLLGATLTF	22,00	1,83
P40197	GPV_HUMAN	GP5	560	16	MLRGTLLCAVLGLLRA	10	GTLLCAVLGL	22,20	2,22
Q12907	LMAN2_HUMAN	LMAN2 C5orf8	356	44	MAAEGWIWRWGWGRRCLGRPGLLGPGPGPTTPLFLLLLLGSVTA	10	TTPLFLLLLL	22,60	2,26
P11215	ITAM_HUMAN	ITGAM CD11B CR3A	1152	16	MALRVLLLTALTLCHG	11	LRVLLLTALTL	22,90	2,08
Q6UWI2	PARM1_HUMAN	PARM1	310	20	MVYKTLFALCILTAGWRVQS	11	TLFALCILTAG	23,00	2,09
O60391	NMD3B_HUMAN	GRIN3B	1043	22	MEFVRALWLGLALALGPGSAGG	10	ALWLGLALAL	23,10	2,31
Q8NCL9	APCDL_HUMAN	APCDD1L	501	24	MPAAMLPYACVLVLLGAHTAPAAG	12	ACVLVLLGAHTA	23,40	1,95
Q9C0A0	CNTP4_HUMAN	CNTNAP4 CASPR4 KIAA1763	1308	25	MGSVTGAVLKTLLLLSTQNWNRVEA	12	VTGAVLKTLLLL	23,50	1,96
P48357	LEPR_HUMAN	LEPR DB OBR	1165	21	MICQKFCVVLLHWEFIYVITA	13	FCVVLLHWEFIYV	23,90	1,84
Q9NPG1	FZD3_HUMAN	FZD3	666	22	MAMTWIVFSLWPLTVFMGHIGG	12	IVFSLWPLTVFM	24,00	2,00
P43489	TNR4_HUMAN	TNFRSF4 TXGP1L	277	28	MCVGARRLGRGPCAALLLLGLGLSTVTG	17	LGRGPCAALLLLGLGLS	24,20	1,42
O15321	TM9S1_HUMAN	TM9SF1	606	27	MTVVGNPRSWSCQWLPILILLLGTGHG	11	WLPILILLLGT	24,40	2,22
P15509	CSF2R_HUMAN	CSF2RA CSF2R CSF2RY	400	19	MLLLVTSLLLCELPHPAFL	11	LLVTSLLLCEL	24,50	2,23
P19224	UD16_HUMAN	UGT1A6 GNT1 UGT1	532	26	MACLLRSFQRISAGVFFLALWGMVVG	13	ISAGVFFLALWGM	24,90	1,92
Q6UVK1	CSPG4_HUMAN	CSPG4 MCSP	2322	29	MQSGPRPPLPAPGLALALTLTMLARLASA	11	LALALTLTMLA	24,90	2,26
P53801	PTTG_HUMAN	PTTG1IP C21orf1 C21orf3	180	32	MAPGVARGPTPYWRLRLGGAALLLLLIPVAAA	18	YWRLRLGGAALLLLLIPV	25,30	1,41
Q13445	TMED1_HUMAN	TMED1	227	23	MMAAGAALALALWLLMPPVEVGG	15	AGAALALALWLLMPP	25,40	2,2
P55808	XG_HUMAN	XG PBDX	180	21	MESWWGLPCLAFLCFLMHARG	14	WWGLPCLAFLCFLM	25,70	1,84
A6H8M9	CDHR4_HUMAN	CDHR4 CDH29 UNQ9392/PRO34300	788	16	MVLLRLLVFLFAPVVS	10	LLRLLVFLFA	26,10	2,61
Q9BXS4	TMM59_HUMAN	TMEM59	323	35	MAAPKGSLWVRTQLGLPPLLLLTMALAGGSGTASA	20	LWVRTQLGLPPLLLLTMALA	26,20	1,31
Q13349	ITAD_HUMAN	ITGAD	1161	17	MTFGTVLLLSVLASYHG	11	FGTVLLLSVLA	26,30	2,39
O95206	PCDH8_HUMAN	PCDH8	1070	29	MSPVRRWGSPCLFPLQLFSLCWVLSVAQS	17	WGSPCLFPLQLFSLCWVLSV	26,70	1,57
P15813	CD1D_HUMAN	CD1D	335	19	MGCLLFLLLWALLQAWGSA	11	LLFLLLWALLQ	26,80	2,44
P32942	ICAM3_HUMAN	ICAM3	547	29	MATMVPSVLWPRACWTLLVCCLLTPGVQG	17	VLWPRACWTLLVCCLLT	27,40	1,61
Q9Y493	ZAN_HUMAN	ZAN	2812	17	MVPPVWTLLLLVGAALF	10	VWTLLLLVGA	27,63	2,45
P12319	FCERA_HUMAN	FCER1A FCE1A	257	25	MAPAMESPTLLCVALLFFAPDGVLA	13	SPTLLCVALLFFA	28,00	2,15
P35916	VGFR3_HUMAN	FLT4 VEGFR3	1363	24	MQRGAALCLRLWLCLGLLDGLVSG	19	GAALCLRLWLCLGLLDGL	28,90	1,52
Q6ZN44	UNC5A_HUMAN	UNC5A KIAA1976 UNC5H1	842	25	MAVRPGLWPALLGIVLAAWLRGSGA	14	LWPALLGIVLAAWL	29,30	2,09
P22310	UD14_HUMAN	UGT1A4 GNT1 UGT1	534	28	MARGLQVPLPRLATGLLLLLSVQPWAES	11	LATGLLLLLSV	29,44	2,61
H3BS89	T178B_HUMAN	TMEM178B	294	23	MAAGRLLLYTGLSLALCALGMLA	14	LLLYTGLSLALCAL	29,50	2,11
P43681	ACHA4_HUMAN	CHRNA4 NACRA4	627	28	MELGGPGAPRLLPPLLLLLGTGLLRASS	14	LLPPLLLLLGTGLL	29,50	2,11
P34998	CRFR1_HUMAN	CRHR1 CRFR CRFR1 CRHR	444	23	MGGHPQLRLVKALLLLGLNPVSA	10	LVKALLLLGL	29,69	2,64
O75074	LRP3_HUMAN	LRP3	770	36	MEKRAAAGLEGAPGARAQLAVVCLVNIFLTGRLSSA	14	AQLAVVCLVNIFLT	29,70	2,12
O94933	SLIK3_HUMAN	SLITRK3 KIAA0848	977	28	MKPSIAEMLHRGRMLWIILLSTIALGWT	10	MLWIILLSTIAL	30,00	3,00
P28472	GBRB3_HUMAN	GABRB3	473	25	MWGLAGGRLFGIFSAPVLVAVVCCA	14	LFGIFSAPVLVAVV	30,21	2,69
P51841	GUC2F_HUMAN	GUCY2F GUC2F RETGC2	1108	50	MFLGLGRFSRLVLWFAAFRKLLGHHGLASAKFLWCLCLLSVMSLPQQVWT	13	FLWCLCLLSVMSL	30,40	2,34
Q9Y5F8	PCDGJ_HUMAN	PCDHGB7	929	30	MGGSCAQRRRAGPRQVLFPLLLPLFYPTLC	10	VLFPLLLPLF	30,98	2,76
P05538	DQB2_HUMAN	HLA-DQB2 HLA-DXB	268	32	MSWKMALQIPGGFWAAAVTVMLVMLSTPVAEA	19	IPGGFWAAAVTVMLVMLST	31,20	1,64
P10586	PTPRF_HUMAN	PTPRF LAR	1907	29	MAPEPAPGRTMVPLVPALVMLGLVAGAHG	12	LVPALVMLGLVA	31,30	2,61
Q5JRA6	TGO1_HUMAN	MIA3 KIAA0268 TANGO UNQ6077/PRO20088	1907	22	MAAAPGLLVWLLVLRLPWRVPG	10	LLVWLLVLRL	31,50	2,80
Q9HD45	TM9S3_HUMAN	TM9SF3 SMBP UNQ245/PRO282	589	28	MRPLPGALGVAAAAALWLLLLLLPRTRA	17	ALGVAAAAALWLLLLLL	31,76	2,83
O14931	NCTR3_HUMAN	NCR3 1C7 LY117	201	18	MAWMLLLILIMVHPGSCA	10	WMLLLILIMV	32,20	3,22
O43184	ADA12_HUMAN	ADAM12 MLTN UNQ346/PRO545	909	28	MAARPLPVSPARALLLALAGALLAPCEA	19	LPVSPARALLLALAGALLA	32,70	1,72
Q9P246	STIM2_HUMAN	STIM2 KIAA1482	746	14	MLVLGLLVAGAADG	10	LVLGLLVAGA	32,79	2,92
Q5VYJ5	MALR1_HUMAN	MALRD1 C10orf112 DIET1	2156	31	MLFFLDRMLAFPMNETFCCLWIACVFNSTLA	10	FCCLWIACVF	33,04	2,94
Q9HB29	ILRL2_HUMAN	IL1RL2 IL1RRP2	575	19	MWSLLLCGLSIALPLSVTA	10	LLLCGLSIAL	33,56	2,99
O00592	PODXL_HUMAN	PODXL PCLP PCLP1	558	22	MRCALALSALLLLLSTPPLLPS	11	CALALSALLLLL	33,70	3,06
P55082	MFAP3_HUMAN	MFAP3	362	21	MKLHCCLFTLVASIIVPAAFV	14	LHCCLFTLVASIIV	33,70	2,41
Q29980	MICB_HUMAN	MICB PERB11.2	383	22	MGLGRVLLFLAVAFPFAPPAAA	12	VLLFLAVAFPFA	33,82	3,02
Q8NC67	NETO2_HUMAN	NETO2 BTCL2 UNQ1926/PRO4401	525	22	MALERLCSVLKVLLITVLVVEG	14	LCSVLKVLLITVLV	34,07	3,04
P32926	DSG3_HUMAN	DSG3 CDHF6	999	23	MMGLFPRTTGALAIFVVVILVHG	17	LFPRTTGALAIFVVVIL	34,30	2,02
P43307	SSRA_HUMAN	SSR1 TRAPA PSEC0262	286	18	MRLLPRLLLLLLLVFPAT	13	LLPRLLLLLLLVF	34,33	3,06
A4D0T7	SIM30_HUMAN	SMIM30	59	24	MTSVSTQLSLVLMSLLLVLPVVEA	14	LSLVLMSLLLVLPV	34,59	3,09
Q96NI6	LRFN5_HUMAN	LRFN5 C14orf146 SALM5	719	17	MEKILFYLFLIGIAVKA	11	ILFYLFLIGIA	35,62	3,18
Q14165	MLEC_HUMAN	MLEC KIAA0152	292	28	MLGAWAVEGTAVALLRLLLLLLPPAIRG	12	AVALLRLLLLLL	35,88	3,20
Q3SY77	UD3A2_HUMAN	UGT3A2 PSEC0073 UNQ842/PRO1780	523	22	MAGQRVLLLVGFLLPGVLLSEA	14	VLLLVGFLLPGVLL	36,13	3,23
Q9Y5E1	PCDB9_HUMAN	PCDHB9 PCDH3H	797	26	MKTRGFSFPRQRQVLFLFLFWGVSLA	8	VLFLFLFW	36,91	3,30
P40967	PMEL_HUMAN	PMEL D12S53E PMEL17 SILV	661	24	MDLVLKRCLLHLAVIGALLAVGAT	10	LAVIGALLAV	37,68	3,37
Q9P2B2	FPRP_HUMAN	PTGFRN CD9P1 EWIF FPRP KIAA1436	879	25	MGRLASRPLLLALLSLALCRGRVVR	10	LLLALLSLAL	37,94	3,39
Q7Z7D3	VTCN1_HUMAN	VTCN1 B7H4 UNQ659/PRO1291	282	24	MASLGQILFWSIISIIIILAGAIA	14	ILFWSIISIIIILA	38,19	3,42
Q5T2D2	TRML2_HUMAN	TREML2 C6orf76 TLT2 UNQ6268/PRO20473	321	18	MAPAFLLLLLLWPQGCVS	9	AFLLLLLLW	38,45	3,44
Q8NC54	KCT2_HUMAN	KCT2 C5orf15 HTGN29	265	49	MAAAVPKRMRGPAQAKLLPGSAIQALVGLARPLVLALLLVSAALSSVVS	12	LVLALLLVSAAL	38,71	3,46
P54753	EPHB3_HUMAN	EPHB3 ETK2 HEK2 TYRO6	998	33	MARARPPPPPSPPPGLLPLLPPLLLLPLLLLPA	13	GLLPLLPPLLLLPLLLL	38,80	2,98
Q96KC8	DNJC1_HUMAN	DNAJC1 HTJ1	554	47	MTAPCSQPAQLPGRRQLGLVPFPPPPPRTPLLWLLLLLLAAVAPARG	13	LLWLLLLLLAAVA	38,97	3,49
Q7L985	LIGO2_HUMAN	LINGO2 LERN3 LRRN6C UNQ9234/PRO31993	606	27	MLHTAISCWQPFLGLAVVLIFMGSTIG	11	FLGLAVVLIFM	39,48	3,53
Q8NFY4	SEM6D_HUMAN	SEMA6D	1073	20	MRVFLLCAYILLLMVSQLRA	13	VFLLCAYILLLMV	39,60	3,05
P42263	GRIA3_HUMAN	GRIA3 GLUR3 GLURC	894	28	MARQKKMGQSVLRAVFFLVLGLLGHSHG	10	AVFFLVLGLL	39,74	3,56
Q5SY80	CTSRE_HUMAN	CATSPERE C1orf101	951	19	MSAREVAVLLLWLSCYGSA	8	VAVLLLWL	40,00	3,58
F5H4A9	CC080_HUMAN	C3orf80	247	35	MWGPGVTAEGLSVAPAPPPLLPLLLLLALALVAPS	13	LLPLLLLLALALV	40,51	3,63
Q8IZU8	DSEL_HUMAN	DSEL C18orf4 NCAG1	1212	20	MALMFTGHLLFLALLMFAFS	9	LLFLALLMF	41,03	3,68
Q9H251	CAD23_HUMAN	CDH23 KIAA1774 KIAA1812 UNQ1894/PRO4340	3354	23	MGRHVATSCHVAWLLVLISGCWG	8	VAWLLVLI	41,29	3,70
Q9Y5F3	PCDB1_HUMAN	PCDHB1	818	28	MAGTRRKSLQNRQVGSLLIFLCISVGDA	9	LLIFLCISV	41,54	3,72
Q15363	TMED2_HUMAN	TMED2 RNP24	201	20	MVTLAELLVLLAALLATVSG	10	LLVLLAALLA	41,80	3,75
Q9NRX6	KISHB_HUMAN	TMEM167B C1orf119 AD-020	74	22	MTNVYSLDGILVFGLLFVCTCA	9	ILVFGLLFV	42,06	3,77
Q9NYQ7	CELR3_HUMAN	CELSR3 CDHF11 EGFL1 FMI1 KIAA0812 MEGF2	3312	32	MMARRPPWRGLGGRSTPILLLLLLSLFPLSQE	10	ILLLLLLSLF	42,32	3,79
Q8IXH8	CAD26_HUMAN	CDH26	832	27	MAMRSGRHPSLLLLLVLLLWLLQVSII	12	LLLLLVLLLWLL	42,57	3,82
Q6PHW0	IYD1_HUMAN	IYD C6orf71 DEHAL1	289	23	MYFLTPILVAILCILVVWIFKNA	14	ILVAILCILVVWIF	42,83	3,84

Fig. 2

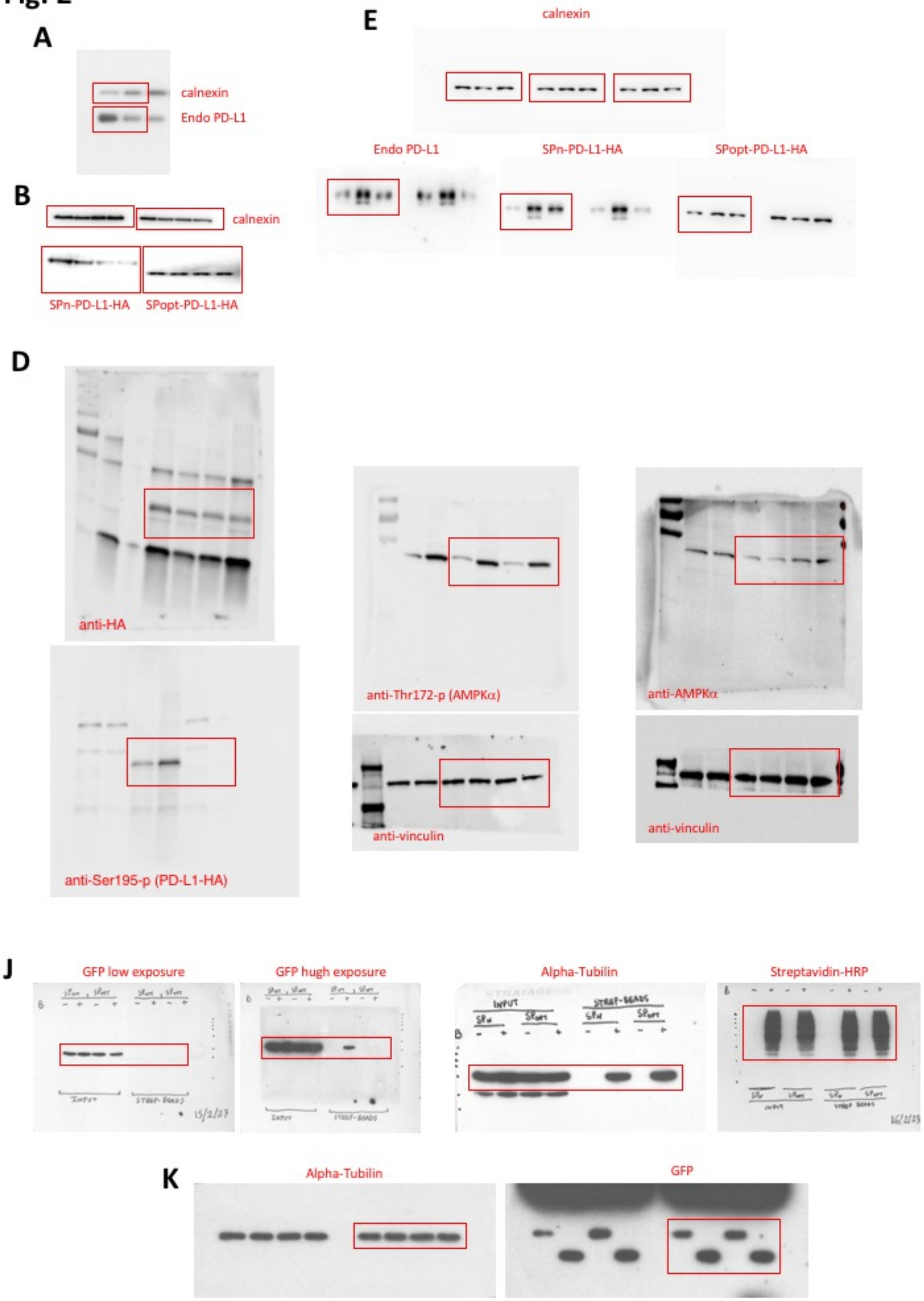
A
calnexin
Endo PD-L1
B
calnexin
SPn-PD-L1-HA
SPopt-PD-L1-HA
E
calnexin
Endo PD-L1
SPn-PD-L1-HA
SPopt-PD-L1-HA
D
anti-HA
anti-Ser195-p (PD-L1-HA)
anti-Thr172-p (AMPKα)
anti-vinculin
anti-AMPKα
anti-vinculin
J
GFP low exposure
GFP hugh exposure
Alpha-Tubilin
Streptavidin-HRP
K
Alpha-Tubilin
GFP

Fig. 3

A

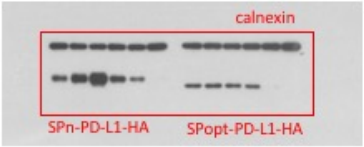
calnexin
SPn-PD-L1-HA
SPopt-PD-L1-HA

B

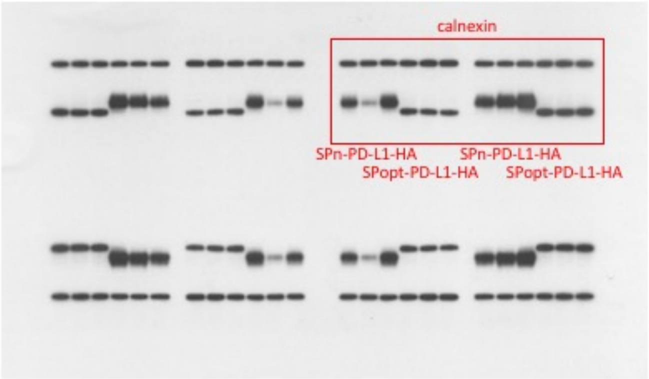
calnexin
SPn-PD-L1-HA
SPopt-PD-L1-HA
SPn-PD-L1-HA
SPopt-PD-L1-HA

F

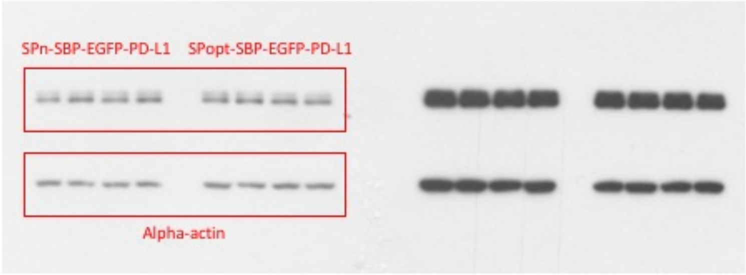
SPn-SBP-EGFP-PD-L1
SPopt-SBP-EGFP-PD-L1
Alpha-actin

Fig. 4

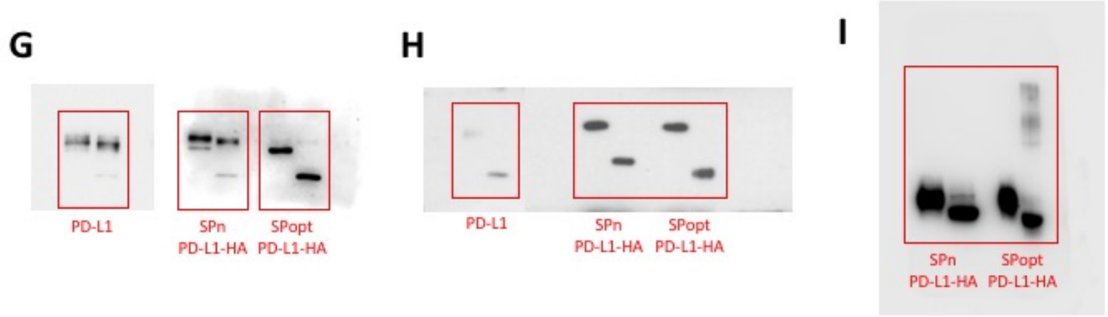
G
PD-L1
SPn PD-L1-HA
SPopt PD-L1-HA
H
PD-L1
SPn PD-L1-HA
SPopt PD-L1-HA
I
SPn PD-L1-HA
SPopt PD-L1-HA

Fig. S2

D

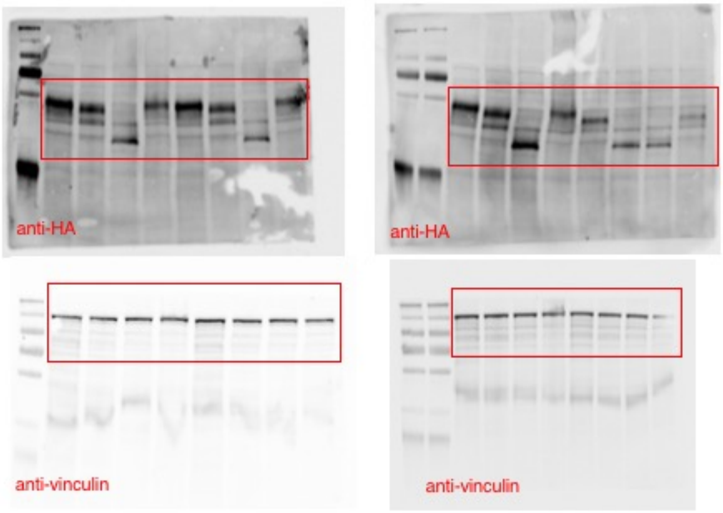

Fig. S3

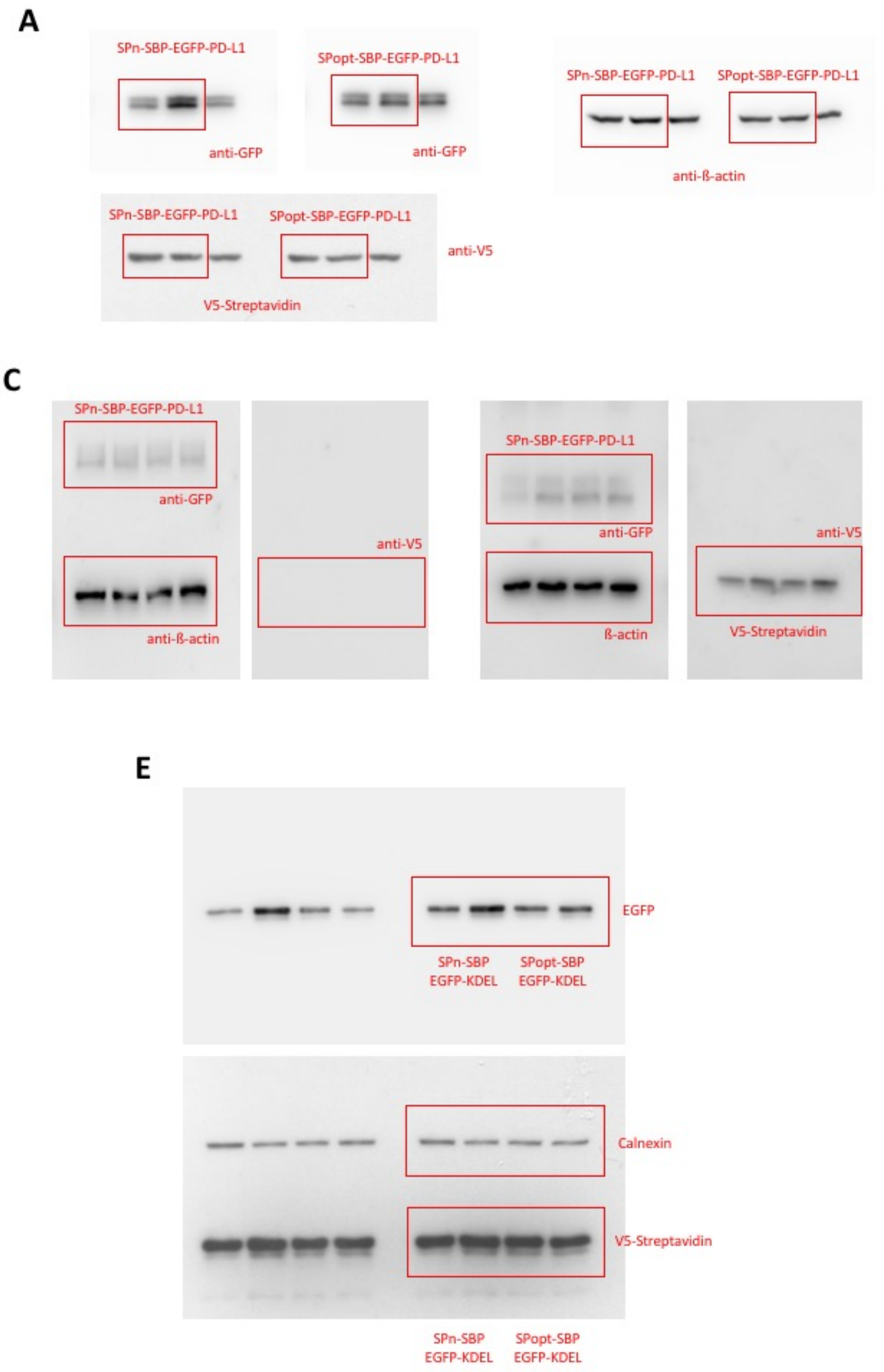
A
SPn-SBP-EGFP-PD-L1
anti-GFP
SPopt-SBP-EGFP-PD-L1
anti-GFP
SPn-SBP-EGFP-PD-L1
SPopt-SBP-EGFP-PD-L1
anti-ß-actin
SPn-SBP-EGFP-PD-L1
SPopt-SBP-EGFP-PD-L1
anti-V5
V5-Streptavidin
C
SPn-SBP-EGFP-PD-L1
anti-GFP
anti-ß-actin
anti-V5
SPn-SBP-EGFP-PD-L1
anti-GFP
ß-actin
anti-V5
V5-Streptavidin
E
EGFP
SPn-SBP
EGFP-KDEL
SPopt-SBP
EGFP-KDEL
Calnexin
V5-Streptavidin
SPn-SBP
EGFP-KDEL
SPopt-SBP
EGFP-KDEL

Fig. S5

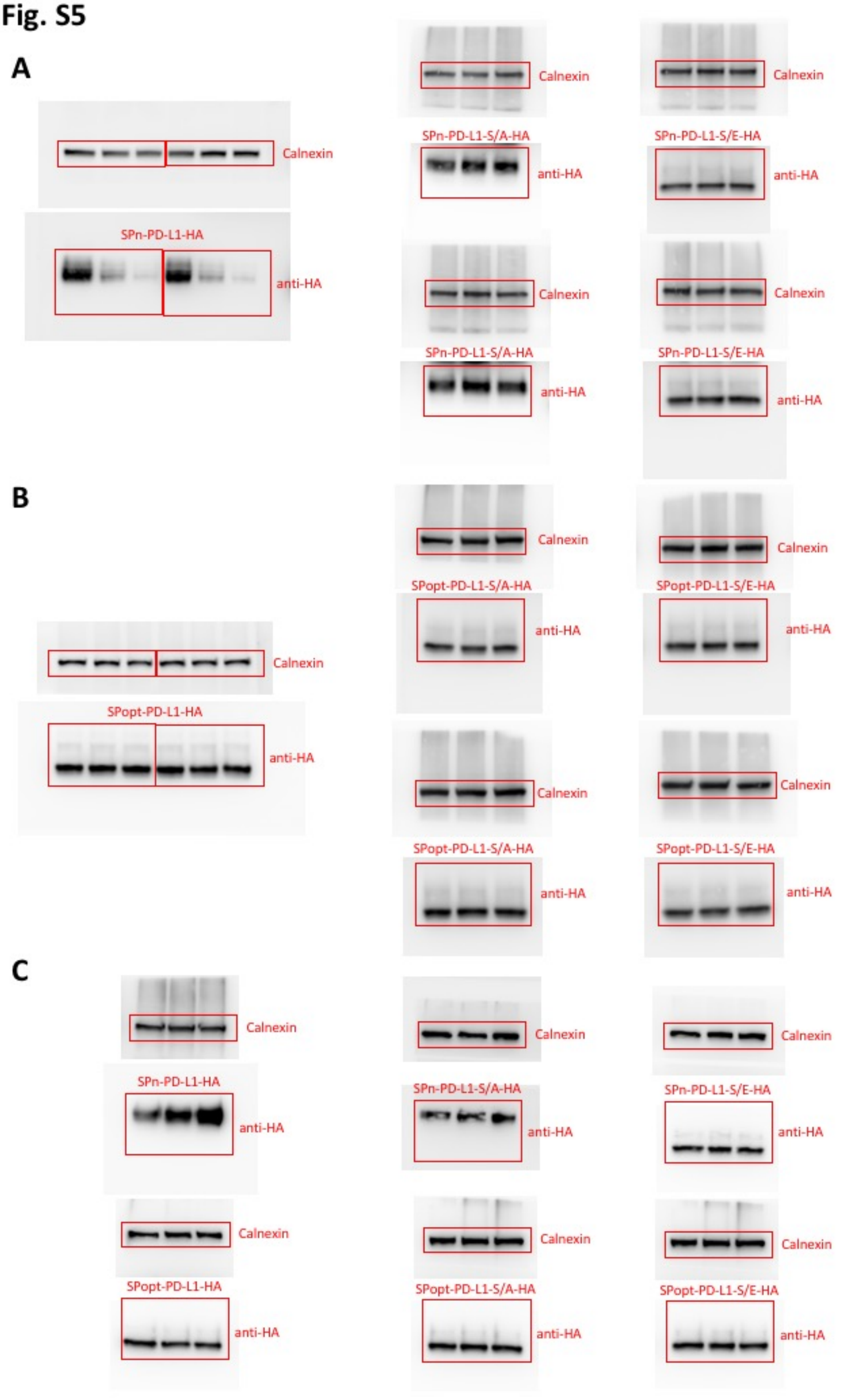
A
Calnexin
SPn-PD-L1-HA
anti-HA
Calnexin
SPn-PD-L1-S/A-HA
anti-HA
Calnexin
SPn-PD-L1-S/E-HA
anti-HA
Calnexin
SPn-PD-L1-S/A-HA
anti-HA
Calnexin
SPn-PD-L1-S/E-HA
anti-HA
B
Calnexin
SPopt-PD-L1-HA
anti-HA
Calnexin
SPopt-PD-L1-S/A-HA
anti-HA
Calnexin
SPopt-PD-L1-S/E-HA
anti-HA
Calnexin
SPopt-PD-L1-S/A-HA
anti-HA
Calnexin
SPopt-PD-L1-S/E-HA
anti-HA
C
Calnexin
SPn-PD-L1-HA
anti-HA
Calnexin
SPn-PD-L1-S/A-HA
anti-HA
Calnexin
SPn-PD-L1-S/E-HA
anti-HA
Calnexin
SPopt-PD-L1-HA
anti-HA
Calnexin
SPopt-PD-L1-S/A-HA
anti-HA
Calnexin
SPopt-PD-L1-S/E-HA
anti-HA

Fig. S6

C

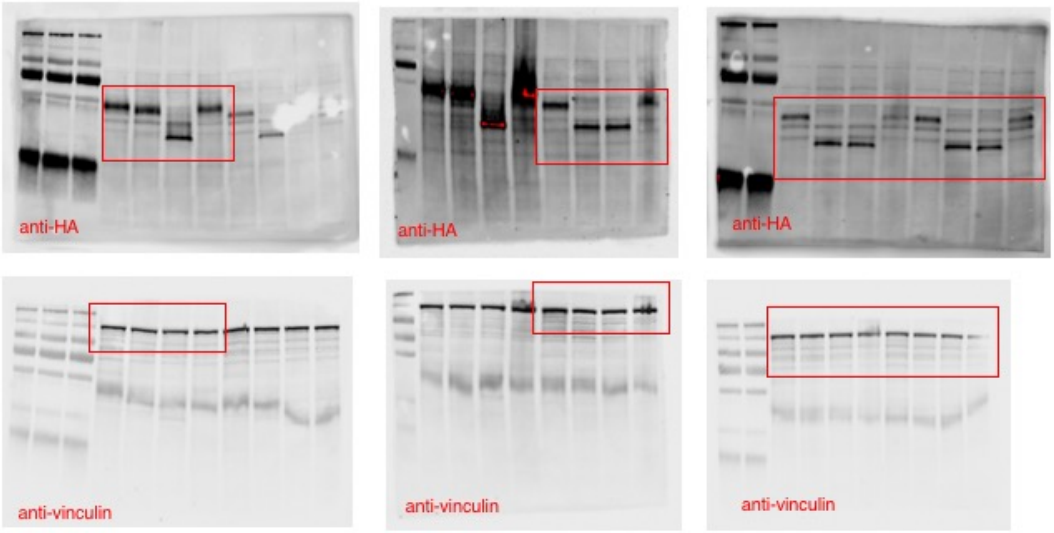
anti-HA
anti-HA
anti-HA
anti-vinculin
anti-vinculin
anti-vinculin

1

Fig. S7

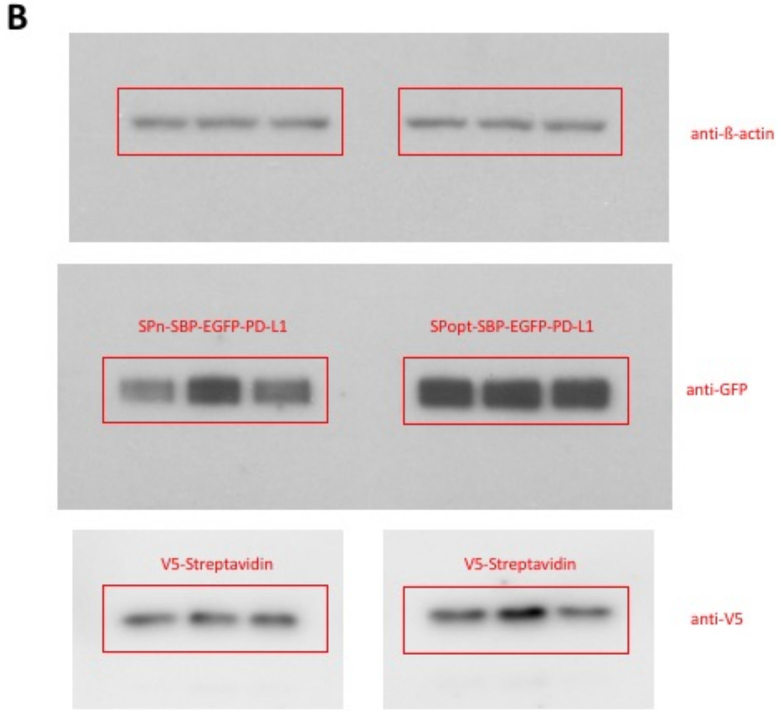
B
anti-ß-actin
SPn-SBP-EGFP-PD-L1
SPopt-SBP-EGFP-PD-L1
anti-GFP
V5-Streptavidin
V5-Streptavidin
anti-V5

1
