# Supplementary material for: A delayed translocation into the endoplasmic reticulum controls the post-translational modifications of PD-L1
Source: Nat Commun. 2026 Apr 11;17:5059. doi: 10.1038/s41467-026-71760-x (PMC13243477; doi:10.1038/s41467-026-71760-x)
Supplement: Supplementary file 2 — Description of Additional Supplementary Information [file 41467_2026_71760_MOESM2_ESM.docx]

**Description of Additional Supplementary Files**

**File Name: Supplementary Data 1**

Description: Hydrophobicity analysis of the H-regions of human signal peptides. The table reports the Kyte-Doolittle hydrophobicity values calculated for the predicted h-region of each human signal peptide (SP). SP sequences were extracted from the Signal Peptide Database (http://www.signalpeptide.de/), and their h-regions were identified using SignalP 6.0 with Eukarya settings. Hydrophobicity values represent the average Kyte-Doolittle score across the h-region. SPs are ranked from lowest to highest hydrophobicity.

**File Name: Supplementary Data 2**

Description: DNA plasmid sequences.

**File Name: Supplementary Data 3**

Description: Experimental conditions.

**File Name: Supplementary Data 4**

Description: Antibodies list.

**File Name: Movie S1**

Description: HeLa cells transiently expressing Cytosolic Streptavidin and SPn-SBP-EGFP-PDL1 in a ratio of 1:1. Release from the ER was induced by addition of biotin 40 μM at 0 min and imaged every 2 minutes by time-lapse confocal microscopy (time scale shown as hr:min) (see also Fig. 1B). Scale bar: 20 μm. Experiments were repeated three times with similar results.

**File Name: Movie S2**

Description: HeLa cells transiently expressing Cytosolic Streptavidin and SPopt-SBP-EGFPPD-L1 in a ratio of 1:1. Release from the ER was induced by addition of biotin 40 μM at 0 min and imaged every 2 minutes by time-lapse confocal microscopy (time scale shown as hr:min) (see also Fig. 1B). Scale bar: 20 μm. Experiments were repeated three times with similar results.

**File Name: Movie S3**

Description: HeLa cells transiently expressing StrepKDEL_SPnA18Y-SBP-EGFP-PD-L1. Streptavidin-KDEL was used as an ER hook. Release from the ER was induced by addition of biotin 40 μM at 0 min and imaged every 2 minutes by time-lapse confocal microscopy (time scale shown as hr:min) (see also Fig. S6G). Scale bar: 20 μm. Experiments were repeated three times with similar results.

**File Name: Movie S4**

Description: HeLa cells transiently expressing StrepKDEL_SPoptA18Y-SBP-EGFP-PD-L1. Streptavidin-KDEL was used as an ER hook. Release from the ER was induced by addition of biotin 40 μM at 0 min and imaged every 2 minutes by time-lapse confocal microscopy (time scale shown as hr:min) (see also Fig. S6G). Scale bar: 20 μm. Experiments were repeated three times with similar results.

**File Name: Movie S5**

Description: HeLa cells transiently expressing StrepKDEL_SPn-SBP-EGFP-PD-L1. StreptavidinKDEL was used as an ER hook. Release from the ER was induced by addition of biotin 40 μM at 0 min and imaged every 2 minutes by time-lapse confocal microscopy (time scale shown as hr:min) (see also Fig. S7A). Scale bar: 20 μm. Experiments were repeated three times with similar results.

**File Name: Movie S6**

Description: HeLa cells transiently expressing StrepKDEL_SPopt-SBP-EGFP-PD-L1. StreptavidinKDEL was used as an ER hook. Release from the ER was induced by addition of biotin 40 μM at 0 min and imaged every 2 minutes by time-lapse confocal microscopy (time scale shown as hr:min) (see also Fig. S7A). Scale bar: 20 μm. Experiments were repeated three times with similar results.
