# Supplementary material for: A delayed translocation into the endoplasmic reticulum controls the post-translational modifications of PD-L1
Source: Nat Commun. 2026 Apr 11;17:5059. doi: 10.1038/s41467-026-71760-x (PMC13243477; doi:10.1038/s41467-026-71760-x)
Supplement: Supplementary file 13 — Transparent Peer Review file [file 41467_2026_71760_MOESM13_ESM.pdf]

nature portfolio

Peer Review File

A delayed translocation into the endoplasmic reticulum controls the post translational modifications of PD-L1

Corresponding Author: Dr Magda Cannata Serio

This file contains all reviewer reports in order by version, followed by all author rebuttals in order by version.

Version 0:

Reviewer comments:

Reviewer #1

(Remarks to the Author)
The authors seek to understand the molecular mechanism underlying a novel role of delayed translocation in the endoplasmic reticulum for PD-L1 post-translational modifications by the cytosolic enzymes and subsequent protein stability control. Using various biochemical and cellular biology approaches, the authors identified a suboptimal signal peptide sequence in PD-L1 and further showed that optimization of the SP can significantly increase its translocation co-translationally to ER. Further studies allowed the authors to come up with a model that the suboptimal SP present in PD-L1 allows the transient exposure of the luminal portion of PD-L1 to cytosolic enzymes to receive various PTM modifications to impact PD-L1 biology. The manuscript is clearly written. However, additional in-depth investigation should be carried out to validate the detailed mechanisms. In addition, the following concerns should be addressed.

1). Figure 1: the authors identified three polar or charged amino acids in the H-region of the SP motif of PD-L1, will mutating these three amino acids in a step wise manner allows the authors to capture a step-wise recovery of the translocation efficacy? Moreover, it will be important to use the CRISPR technology to generate mutation directly on the endogenous PD-L1 gene to monitor the changes in PD-L1 translocation or stability.
2). Figure 2A-B, metformin is not a specific AMPK agonist, it will be better to use specific AMPK agonist like 911 and/or A-769662 to repeat these assays.
3). Figure 2B: This is not a physiological experimental setting, it is better for the authors to use CRISPR technology to generate mutation directly on the endogenous PD-L1 gene to monitor the changes in PD-L1 stability after activating AMPK by AMPK specific agonists, or by physiological cue such as glucose deprivation.
4). Figure 2C, similarly, compound C is not a specific AMPK inhibitor, the authors should use shRNA or CRISPR to deplete endogenous AMPK to monitor its changes in endogenous PD-L1 stability, and in the generated endogenous SP-optimized PD-L1 cell line.
5). It will be nice for the authors to use CRISPR to mutate the S195 residue to Ala and then examine if the protein stability of mutant PDL1 respond to AMPK manipulation (including AMPK agonist treatment, or AMPK depleting).
6). Figure 2; the authors should gather direct evidence to show that mutating SP site can affect the phosphorylation of PD-L1 on Ser195, either by antibody to pick up this specific phosphorylation event, or by mass spectrometry analysis.
7). Figure 3A-C, the authors should perform the same set assays in AMPK KO cell lines to show that they are AMPK-dependent.
8). Figure 4, the biological significance of this finding should be further explored. As PD-L1 protein levels control tumor cell response to PD-1 blockade, it will be important for the authors to carry out syngeneic mouse modeling to illustrate its effects to immune checkpoint blockade treatment.

Reviewer #2

(Remarks to the Author)
Review of manuscript of Serio et al. entitled: ‘A delayed translocation into the endoplasmic reticulum controls the post translational modifications of PD-L1’

This manuscript focuses on the relatively inefficient ER targeting signal peptide of PD-L1 that confers delayed ER insertion that appears to be required for PD-L1 nascent polypeptide modifications on the cytosolic side of the ER membrane,

preceding final membrane translocation. The finding that delayed ER targeting or translocation is required for both cytosolic and ER luminal modifications of the PD-L1 mature polypeptide is very interesting. Another question that the authors may be able to address is how pervasive delayed translocation among signal peptide-containing human proteins is. As some “weak” signal peptides may be targeted by client-selective Sec61 inhibitors, identifying them could have interest for therapy development.

Major points:

1. Could the authors provide more extensive quantitation of the low hydrophobicity of PD-L1 SP relative to all other human SPs? Are there other low hydrophobicity SPs that the authors could identify? It would increase general interest of this manuscript if the authors could identify other signal peptides with delayed insertion properties. I would suggest attempting to identify them bioinformatically and testing delayed translocation with for example the CytoRUSH assay.
2. I am not convinced of the current analysis of the PD-L1 SP hydrophobicity. Please explain how the h-region of each human SP was identified and how was the Kyte-Doolittle score calculated for each SP with differing lengths. For example, were SPs aligned based on the predicted SP cleavage site? I would like to see a more detailed analysis of h-region hydrophobicity of PD-L1 compared to all other human SPs. How different is PD-L1 in hydrophobicity or does it have some other biophysical properties that distinguish it from other SPs? What supports the notion that PD-L1 has a suboptimal SP?
3. In the Discussion the authors discuss competition between prokaryotic SRP and Trigger factor and possible competitive processes with SRP in eukaryotes. Here, it would be useful to discuss the mechanism of competitive protein targeting by NAC and SRP that Ban and Deuerling groups have recently characterized.

Minor points:

1. Page 2. The sentence “Recently, it has been shown that some proteins with a SP can be fully synthetized in the cytosol and translocated into the ER only at the end of the synthesis in a post-translational manner.”. This has in fact been known for quite some time, I would remove “Recently”.
2. Page 3. “Despite lacking a signal sequence for ER translocation, luminal localization has been proposed for these kinases.”. I am not aware of such publications, please provide references or remove this sentence.
3. Page 4. “Previous studies have shown that PD-L1 luminal domain is modified by several cytosolic kinases.”. Please include references for this statement.
4. Page 5. The authors cite metformin as a well-characterized AMPK activator, but no reference is given. It would be ideal if the authors could demonstrate activation of AMPK following their treatment by for examples demonstrating a downstream AMPK substrate phosphorylation.
5. The authors state the PD-L1 SP may be inefficient in membrane translocation, but it is also quite likely inefficient in engaging with SRP and in subsequent ER targeting of the RNC.
6. Page 6. aTubulin should be spelled a-tubulin (with alpha instead of a of course).
7. It is interesting that the slower translation rate prevents exposure of mature part of PD-L1 to the cytosol. Do the authors assume that this is reflecting stalled ER translocation by the native PD-L1 SP? In other words, the native PD-L1 SP would remain bound at the Sec61 translocon or would have low propensity to insert through the Sec61 pore and the lateral gate.
8. Page 9. The authors state: “it was proposed that a fraction of these different cytosolic kinases is translocated into the lumen of the ER, despite the absence of a SP”. Please provide a reference for this statement.
9. Page 10. “pooper insertion of the SP” should read “proper insertion”.

Reviewer #3

(Remarks to the Author)
In the manuscript “A delayed translocation into the endoplasmic reticulum controls the post translational modifications of PD-L1” Cannata Serio et al. describe a how sub-optimal signal-peptide sequences can delay translocation into the ER and open up the opportunity for enzymes to modify the nascent protein in the cytoplasm before being translocated into the ER. The authors use PD-L1 as example and show that an optimized SP, delayed protein translation by cycloheximide or introduction of a large linker, abolish AMPK-dependent phosphorylation of a serine 195 in PD-L1. Moreover, the authors show that protein transport of PD-L1 from the ER is perturbed if the SP is exchanged to an optimized SP, indicating viable post-translational modifications are missing.
The manuscript addresses a mechanism for regulation of expression of transmembrane and secretory proteins that is largely unknown and demonstrates well how different alterations impact the AMPK-dependent phosphorylation of PD-L1. The authors provide mechanistical insights on how cytosolic kinases would be able to add PTMs to proteins that are directly translated into the ER, a mechanism that has not been described before. The authors make clever use of novel assays such as cytoRUSH which was developed for this study, and methods such as TurboID-based biotinylation which was repurposed in this study to clarify the role of SPs in protein modification. The manuscript itself is well structured and I could easily follow the authors train of thought.

Overall, I think quality of the study and the shown technical expertise warrant a publication in Nature Communications. However, the story misses in my opinion two things that should be addressed before I support its publication without reservations:

A) I think it is very exciting that one can not only see that optimization of SP seems to prevent AMPK—mediated degradation of the PD-L1, a fact that is shown by the authors in very convincing fashion, but on the other hand also defects its trafficking from the ER. In its current state the manuscript only briefly covers the observed phenotype but lacks further information and speculation, although I believe addition information could improve the strength of the manuscript. The authors do report that the trafficking is affected but do not speculate about why the trafficking is affected. Is it possible that this is due to another PTM that cannot occur?
Here is my suggestion how this could be addressed:

1) The authors could clarify which step of the secretion pathway is affected:
Therefore, the analysis and interpretation of the microscopy data could be improved.
Importantly, it needs to be noted, that the SPopt-PDL1 seems to accumulate in punctuated structures that are not positive for calnexin but probably positive for KDEL (although the cell that was picked here, shows KDEL signal nearly everywhere, compared to the cell that was picked for the SPn fusion protein).
Furthermore, I would not say that the SPopt-PDL1 shows increased colocalization with the Golgi-marker GM130. From the microscopy image you can clearly see that they barely overlap (also the Pearson suggests that). The fact that the signal is increased, is rather caused by the protein being stuck somewhere along the early secretory pathway.
The authors could perform experiments with an ERGIC (ERGIC53 for example) and ER-exit site marker (Sec16 for example), to pin point which step of the secretion pathway is affected. The performed experiment to test the glycosylation status (Fig. 4 G-H) also strongly suggest that ER export is affected by the optimized SP. In addition, I would advise to use the Manders-coefficient for analysis instead of the Pearson. Specifically, when comparing with KDEL one can see that both proteins seem to perfectly colocalize when the SPopt-variant is used, indicating that it overlaps with KDEL positive structures (likely ER-exit sites).

2) it would be great to propose a reason why the protein is not exported properly. Is it because it is lacking a specific PTM (probably a phosphorylation?). It would be great if the authors could speculate why the protein is stuck in the early secretory pathway to round up the experimental finding.

B) To round up the discussion it would be good to know whether it is a common feature of secretory proteins with a suboptimal SPs to have multiple sites for PTMs in their N-terminal region after the SP? Are the authors aware of other proteins that possess suboptimal SPs? And would these proteins also rely on slower translocation so that specific PTMs can be made?

Minor points:
1) the authors should consider showing the differences in amino acids of SPn and SPopt as part of Fig. 1, to make it easier to follow

2) Fig.1B: The images suggest, the EGFP-PD-L1 expression is higher in SPn cells after biotin addition compared the cells that have the SPopt and thus constantly express the EGFP-PD-L1, however the authors show in FigS2A+B that this not the case, similar to what I would expect. If this is simply caused by different contrast settings for the two sets of images, I would suggest to adjust the contrast for the lower panel to avoid confusion.

3) Fig.1E,F: I don't understand “relative” intensity of EGFP, relative to what? I cannot find this information in the methods (although I might have overlooked it). Also Fig.1F says (extra) instead of surface, is this something different or was overlooked when harmonizing the manuscript?

4) Fig 2H: I assume it is not an anti-streptavidin-antibody but rather HRP-tagged streptavidin?
Why both high expression EGFP and low expression EGFP are shown, when only under high EGFP expression biotinylation can be observed? I suspect two different enhancers are used? Also, it is not discussed in the manuscript why two different expression levels are shown.

Reviewer #4

(Remarks to the Author)
The authors show an interesting and important new regulatory mechanism for PD-L1 expression and modification. They show that the sub-optimal hydrophobicity of the H region of the signal peptide causes “delayed translocation” allowing a temporary exposure of the nascent chain to the cytosolic environment, enabling modifications of exoplasmic domains via cytosolic enzymes. They show optimizing the hydrophobicity of the signal peptide of PD-L1 affects the cytosolic exposure, disrupting PD-L1's trafficking, maturation, and localization. The results are novel and of interest to the large number of investigators interested in immune checkpoints.

1. “This pause allows several amino acids to be synthesized, exposing the nascent chain to the cytosolic environment.” Make some comment on how many amino acids “several” is. Ser195 modification is affected which is more than several.

2. Write out full name of SBP, describe. The SBP-tag sequence is 38 amino acids long (or whatever used) and binds to

streptavidin with an equilibrium dissociation constant of 2.5 nM. Reference Barrette PMCID: PMC3640474. Also say whether it is placed before or after the predicted signal cleavage site.

3. In Fig 1B, indicate time scale hr:min or min:sec?

4. Fig 1C-F needs a control of no biotin for each of the time points.

5. Typo: through PTMs 18 (Cha, 2018 #13)

6. In 1D, why are there 2 probings for vinculin? If this is two blots, separate into 2 groupings.

7. Page 6. “SPopt would alleviate this lagging time”. Explain more clearly in context of preceding sentence. Explain better why “slower translation rate, would prevent exposure of S195 in the cytosol “

8. “the SPn-PD-L1-HA and the SPopt-PD-L1-HA sequences were generated from synthetic genes”. Specify whether these are native sequences of PD-L1 or codon-optimized. If codon-optimized, discuss how this might affect results.

9. In sup fig 1A, C and others showing sequence, use a monospaced font like Courier.

10. Move Sup Fig 1C to main Fig 1.

11. Supp Table S2. Currently calculated for 100 random SP. Replace with a Table S2A with 100 random cell surface SP and Table S2B with 100 random secreted SP.

12. Somewhere in Suppl, indicate treatments shown in each of 6 supplemental videos.

13. If they wish, the authors could comment in the discussion that use of native signal peptides in recombinant constructs will best reflect natural functions of the protein.

Reviewer #5

(Remarks to the Author)
I co-reviewed this manuscript with one of the reviewers who provided the listed reports. This is part of the Nature Communications initiative to facilitate training in peer review and to provide appropriate recognition for Early Career Researchers who co-review manuscripts.

Version 1:

Reviewer comments:

Reviewer #1

(Remarks to the Author)
The authors have addressed most of the raised concerns.

Reviewer #2

(Remarks to the Author)
Review of manuscript of Serio et al. entitled: ‘A delayed translocation into the endoplasmic reticulum controls the post translational modifications of PD-L1’

The authors have carried out most of the analyses and experiments requested by the reviewers and in my view, this has significantly strengthened the conclusions of the manuscript. The finding that both an optimized SP sequence and rate of elongation are both important for production of mature PD-L1 suggests complex requirement for optimization of co-translational ER insertion kinetics. While many mechanistic questions regarding the delayed PD-L1 ER translocation process remain open for future studies, this is an important work highlighting a specialized function of a seemingly inefficient signal peptide with implication for optimized protein folding and quality control with possible relevance in recombinant protein production.

I only have one minor remaining questions for the authors before the article is ready for publication.

1. On page 5. the authors state that preprolactin signal peptide (as well as TRH and STATH) would render the tested PD-L1 construct to be targeted to the ER post-translationally and this would explain the biotin-dependent rescue of cell surface expression. Preprolactin is a well-known efficiently targeting signal peptide and I do not find a reason why it would render the rather long PD-L1 construct to be targeted post-translationally. Without further evidence showing that the tested PD-L1 construct with a preprolactin SP its targeted post-translationally, I would not include this notion in the main text.
2. This figure (Fig. S6) would benefit from showing the SP sequences that were tested and including their calculated h-region hydrophobicity values. At the moment, it is not clear from the figure which SPs are presumed to be efficiently targeted

based on their predicted biophysical properties.

Reviewer #3

(Remarks to the Author)
I am happy with the authors revisions and answers and support publication.

Reviewer #4

(Remarks to the Author)
The authors have done additional experimental work, clarified their explanations, and addressed my concerns. I have a few minor suggestions.

1. Usual abbreviated name is PD-L2, not PD1-L2.

2. “our findings emphasize that the use of native, non-optimized SPs in recombinant constructs is essential to faithfully reproduce the natural maturation, trafficking and regulatory properties of the corresponding proteins. “
This is too definitive a claim, re-phrase more cautiously such as
“our findings emphasize that the use of native, non-optimized SPs in recombinant constructs is important to faithfully reproduce the natural maturation, trafficking and regulatory properties of the corresponding proteins and that the use of optimized SP in recombinantly produced proteins may lead to the loss of some of these features. “

3. Highlight PD-L1 signal sequence in Tables.

4. On page 4 of Supp, “Both SPn and PD-L1 correspond to the native sequences extracted from NCBI.” Say “native DNA sequence” so there is no ambiguity with protein sequence.

Reviewer #5

(Remarks to the Author)
I co-reviewed this manuscript with one of the reviewers who provided the listed reports. This is part of the Nature Communications initiative to facilitate training in peer review and to provide appropriate recognition for Early Career Researchers who co-review manuscripts.

Version 2:

Reviewer comments:

Reviewer #2

(Remarks to the Author)
The authors have now addressed all of my remaining concerns. The manuscript is now ready to be published without delay.

Reviewer #4

(Remarks to the Author)
The authors have addressed my concerns.

Open Access This Peer Review File is licensed under a Creative Commons Attribution 4.0 International License, which permits use, sharing, adaptation, distribution and reproduction in any medium or format, as long as you give appropriate credit to the original author(s) and the source, provide a link to the Creative Commons license, and indicate if changes were made.
In cases where reviewers are anonymous, credit should be given to 'Anonymous Referee' and the source.
The images or other third party material in this Peer Review File are included in the article's Creative Commons license, unless indicated otherwise in a credit line to the material. If material is not included in the article's Creative Commons license and your intended use is not permitted by statutory regulation or exceeds the permitted use, you will need to obtain permission directly from the copyright holder.
To view a copy of this license, visit https://creativecommons.org/licenses/by/4.0/

REVIEWER COMMENTS

REVIEWER #1 (REMARKS TO THE AUTHOR):

The authors seek to understand the molecular mechanism underlying a novel role of delayed translocation in the endoplasmic reticulum for PD-L1 post-translational modifications by the cytosolic enzymes and subsequent protein stability control. Using various biochemical and cellular biology approaches, the authors identified a suboptimal signal peptide sequence in PD-L1 and further showed that optimization of the SP can significantly increase its translocation co-translationally to ER. Further studies allowed the authors to come up with a model that the suboptimal SP present in PD-L1 allows the transient exposure of the luminal portion of PD-L1 to cytosolic enzymes to receive various PTM modifications to impact PD-L1 biology. The manuscript is clearly written. However, additional in-depth investigation should be carried out to validate the detailed mechanisms. In addition, the following concerns should be addressed.

1). Figure 1: the authors identified three polar or charged amino acids in the H-region of the SP motif of PD-L1, will mutating these three amino acids in a step wise manner allows the authors to capture a step-wise recovery of the translocation efficacy? Moreover, it will be important to use the CRISPR technology to generate mutation directly on the endogenous PD-L1 gene to monitor the changes in PD-L1 translocation or stability. PD-L1 expression is controlled at different levels from transcription to trafficking.

We thank the reviewer for this suggestion. Two points are addressed here:

- Regarding stepwise mutagenesis of the H-region: we performed a systematic leucine scanning mutagenesis of the three polar/charged residues in the H-region of the PD-L1 SP (T11→L, Y12→L, H14→L), generating 1L, 2L, and 3L-SPopt variants (new Supplemental Fig. 2A). 1L and 2L behaved similarly to the native SP, leading to normal PD-L1 trafficking with accumulation at the plasma membrane and distribution throughout the entire secretory pathway. In contrast, the triple mutant SP-3L (SPopt) caused a dramatic trafficking defect: PD-L1 accumulated in the endoplasmic reticulum and failed to progress through the secretory pathway (new Supplemental Fig. 2B-C). These results, obtained by cell imaging, were fully supported by biochemical analysis of PD-L1 glycosylation. Native PD-L1, as well as 1L and 2L variants, were fully glycosylated and insensitive to PNGaseF or O-glycosidase, while being sensitive to EndoH, consistent with efficient passage through the Golgi apparatus. In contrast, the 3L-SPopt variant was only partially glycosylated and remained fully sensitive to EndoH and PNGaseF,

indicating ER retention and impaired exit from the ER (new Supplemental Fig. 2D). Together, these data support the notion that replacing polar residues in the H-region with leucines progressively increases hydrophobicity but only the full SPopt mutant disrupts PD-L1 maturation, confirming that the native SP sequence is finely tuned to support proper translocation and trafficking.

- Regarding the analysis of endogenous PD-L1 using a CRISPR/CAS9 approach. We are not studying here the function of PD-L1 but rather uncovering the original pathway followed by this protein to translocate into the ER and the role of this pathway in trafficking and modification of PD-L1. We are not questioning here how PD-L1 expression is globally regulated at multiple levels: epigenetic, transcriptional, post-transcriptional, translational and post-translational. This would require a fully independent study.

2). Figure 2A-B, metformin is not a specific AMPK agonist, it will be better to use specific AMPK agonist like 911 and/or A-769662 to repeat these assays.

We thank the reviewer for this important suggestion. In line with this comment, we tested two more specific AMPK agonists (A769662 (Cool B. *et al.*, Cell Metab, 2006)) and AICAr (Dai Y. L. *et al.*, Int J Biol Sci, 2015, Kai Y. *et al.*, Reprod Biol Endocrinol, 2015)), which we show in the new Supplemental Figure S5. We analysed the effect of these agonists on PD-L1 expression for both SPn and SPopt and in the context of non-phosphorylatable (S195A) or pseudo-phosphorylated (S195E) PD-L1 mutants.

3). Figure 2B: This is not a physiological experimental setting, it is better for the authors to use CRISPR technology to generate mutation directly on the endogenous PD-L1 gene to monitor the changes in PD-L1 stability after activating AMPK by AMPK specific agonists, or by physiological cue such as glucose deprivation.

We thank the reviewer for this comment. As mentioned above, endogenous PD-L1 expression is broadly regulated at multiple levels, including transcription, mRNA stability, translation and diverse post-translational mechanisms. It has been shown before that endogenous PD-L1 stability and expression level can be modified through AMPK modulation (Cha J. H. *et al.*, Mol Cell, 2018). What we report here is that AMPK-dependent modulation of PD-L1 stability is unexpectedly linked to the nature of its SP. Importantly, AMPK signaling controls numerous cellular processes, such as global protein synthesis, metabolic adaptation and chromatin

modification (Gongol B. *et al.*, Int J Mol Sci. 2018), all of which can indirectly influence endogenous PD-L1 abundance. For this reason, pharmacological manipulation of AMPK activity may alter endogenous PD-L1 levels independently of its ability to phosphorylate PD-L1 at S195.

To specifically assess the link between activity and PD-L1 signal peptide, we relied on exogenous PD-L1 cDNA constructs, which bypass upstream physiological regulatory layers. This approach allows us to uncouple SP-dependent translocation and S195 phosphorylation from the broader effects of AMPK activation on endogenous PD-L1 expression. The use of CRISPR/CAS9 will be relevant in subsequent functional studies.

4). Figure 2C, similarly, compound C is not a specific AMPK inhibitor, the authors should use shRNA or CRISPR to deplete endogenous AMPK to monitor its changes in endogenous PD-L1 stability, and in the generated endogenous SP-optimized PD-L1 cell line.

We thank the reviewer for this suggestion. However, shRNA- or CRISPR-mediated depletion of AMPK requires long-term selection, during which cells can undergo metabolic and transcriptional adaptations to the absence of AMPK. Such compensatory changes could indirectly influence endogenous PD-L1 expression and stability, making it difficult to attribute the observed effects specifically to AMPK inhibition or loss of S195 phosphorylation.

To avoid these confounding adaptations, we instead employed the more specific AMPK inhibitor SBI-0206965 (Dite T. A. *et al.*, J Biol Chem, 2018). As now shown in the new Supplemental Figure S5, we tested its effects on both SPn- and SPopt-PD-L1, and in the context of the non-phosphorylatable (S195A) and pseudo-phosphorylated (S195E) PD-L1 mutants.

5). It will be nice for the authors to use CRISPR to mutate the S195 residue to Ala and then examine if the protein stability of mutant PDL1 respond to AMPK manipulation (including AMPK agonist treatment, or AMPK depleting).

We thank the reviewer for this suggestion. As discussed above, editing the endogenous PD-L1 locus by CRISPR to generate an S195A mutation would introduce additional layers of regulation upstream of translation, including transcriptional, epigenetic, and mRNA-stability mechanisms, all of which could confound the interpretation of AMPK-dependent effects on PD-L1 stability. In addition, the role of S195 phosphorylation in tuning PD-L1 stability have

been reported before (Cha J. H. *et al*., Mol Cell, 2018). The originality of our study is to uncover the link with the particular nature of PD-L1 signal peptide.

For this reason, we chose to assess the role of S195 phosphorylation using exogenous PD-L1 constructs combined with pharmacological modulation of AMPK activity. Specifically, we tested both AMPK agonists and inhibitors on non-phosphorylatable (S195A) and pseudo-phosphorylated (S195E) PD-L1 mutants expressed under either the native suboptimal signal peptide (SPn) or the optimized signal peptide (SPopt) (new Supplemental Figure S5).

As expected, ^{SPn}S195A behaves similarly to SPn-PD-L1, showing normal trafficking and glycosylation patterns, consistent with the fact that this mutant simply prevents AMPK-dependent phosphorylation without altering the delayed-translocation mechanism imposed by SPn. In contrast, ^{SPn}S195E is retained in the ER, shows altered glycosylation, and is fully sensitive to PNGase F and Endo H, indicating impaired maturation and ER exit, confirming previously reported data (Cha J. H. *et al*., Mol Cell, 2018) (new Supplemental Figure S5 and S6).

Importantly, both ^{SPopt}S195A and ^{SPopt}S195E mutants are retained in the ER and fully sensitive to PNGase F and Endo H, exactly as observed for SPopt alone. This supports the conclusion that replacing the native suboptimal SP with SPopt abolishes delayed translocation and forces efficient co-translational translocation into the ER, thereby eliminating the cytosolic window in which S195 phosphorylation could normally occur. Consequently, in the SPopt context, the phosphorylation state of S195 no longer contributes to PD-L1 folding, trafficking, or stability, as the protein is not exposed to cytosolic kinases prior to ER entry (new Supplemental Figure S5 and S6).

Altogether, these results validate our approach and confirm that using exogenous PD-L1 mutants allows a more direct and interpretable evaluation of how AMPK activity regulates PD-L1 stability specifically through S195 phosphorylation, independently of endogenous pre-translational regulatory layers.

6). Figure 2; the authors should gather direct evidence to show that mutating SP site can affect the phosphorylation of PD-L1 on Ser195, either by antibody to pick up this specific phosphorylation event, or by mass spectrometry analysis.

This is indeed an important point that we addressed in Figure 2D where we directly assessed S195 phosphorylation using a phospho-Ser195 specific antibody. As shown, PD-L1 is

phosphorylated on S195 only when expressed with its native signal peptide (SPn), and this phosphorylation is further increased upon metformin treatment. In contrast, PD-L1 equipped with SPopt shows no detectable phosphorylation on S195, either in basal conditions or after metformin stimulation.

These data demonstrate that mutations in the SP indeed control the phosphorylation status of S195, thereby establishing a direct functional link between SP-dependent translocation kinetics and AMPK-mediated modification of PD-L1.

7). Figure 3A-C, the authors should perform the same set assays in AMPK KO cell lines to show that they are AMPK-dependent.

We thank the reviewer for this suggestion. However, long-term AMPK ablation may trigger compensatory cellular adaptations that would indirectly alter PD-L1 expression or stability, thereby confounding the interpretation of AMPK-dependent effects.

As shown in the new Supplemental Figure S5, we thus decided employed two additional highly specific AMPK activators and one additional specific AMPK inhibitor. We tested these compounds on both $^{\text{SPn-}}$ and ^{SPopt}PD-L1, in the context of the non-phosphorylatable (S195A) and pseudo-phosphorylated (S195E) PD-L1 mutants. These analyses consistently demonstrate that the observed effects are AMPK-dependent and require both the native SPn and an intact S195 residue.

8). Figure 4, the biological significance of this finding should be further explored. As PD-L1 protein levels control tumor cell response to PD-1 blockade, it will be important for the authors to carry out syngeneic mouse modeling to illustrate its effects to immune checkpoint blockade treatment.

As indicated above, carrying out a functional study of PD-L1, in particular using a mouse model, would require a complete and dedicated project for several reasons: 1) PD-L1 SPs are highly divergent between mouse and human, with very limited sequence conservation. As a consequence, the TY-H/LL-L optimisation that we introduced in the human SP cannot be reproduced in the mouse SP through an equivalent amino-acid substitution. A dedicated optimisation strategy would need to be developed specifically for the mouse SP, requiring a preliminary screening in mouse cell lines to identify which substitutions, if any, recapitulate

the effects observed in the human system. 2) The TY-H/LL-L substitution was designed exclusively to enhance PD-L1 translocation efficiency and to mechanistically dissect the impact of SP optimisation on AMPK-dependent post-translational regulation. Its purpose was not to model a naturally occurring mutation, as this SP variant has not been identified in humans. 3) Our data already demonstrate that SP optimisation profoundly affects PD-L1 maturation, glycosylation and cell-surface expression, features that are expected to increase immune-evasion capacity and resistance to PD-1 blockade. These mechanistic insights fulfill the main biological question raised by the reviewer without requiring in vivo modeling.

It would also require quantitative in vivo analysis of PD-L1 activity in mice which would be expert and demanding. In addition, perturbing delayed translocation would be expected to prevent, or reduce, PD-L1 activity and such a negative result may be also due to confounding effects.

For these reasons, we think that it is beyond the frame of our current study to undertake the extensive effort required to develop a syngeneic mouse model for a mutation that does not naturally occur in vivo and whose mouse equivalent is not predictable.

REVIEWER #2 (REMARKS TO THE AUTHOR):

Review of manuscript of Serio et al. entitled: ‘A delayed translocation into the endoplasmic reticulum controls the post-translational modifications of PD-L1’

This manuscript focuses on the relatively inefficient ER targeting signal peptide of PD-L1 that confers delayed ER insertion that appears to be required for PD-L1 nascent polypeptide modifications on the cytosolic side of the ER membrane, preceding final membrane translocation. The finding that delayed ER targeting or translocation is required for both cytosolic and ER luminal modifications of the PD-L1 mature polypeptide is very interesting. Another question that the authors may be able to address is how pervasive delayed translocation among signal peptide-containing human proteins is. As some “weak” signal peptides may be targeted by client-selective Sec61 inhibitors, identifying them could have interest for therapy development.

Major points:

1. Could the authors provide more extensive quantitation of the low hydrophobicity of PD-L1 SP relative to all other human SPs? Are there other low hydrophobicity SPs that the authors could identify? It would increase general interest of this manuscript if the authors could identify

other signal peptides with delayed insertion properties. I would suggest attempting to identify them bioinformatically and testing delayed translocation with for example the CytoRUSH assay.

We thank the reviewer for this excellent suggestion. In response, we performed an extensive bioinformatic analysis aimed at quantitatively positioning the PD-L1 signal peptide (SP) within the landscape of all human SPs and identifying additional candidate SPs with delayed or non-canonical translocation properties.

First, we extracted 3,125 annotated human SPs from the Signal Peptide Database (http://www.signalpeptide.de). For each protein, the h-region was predicted using SignalP 6.0 (Eukarya model), and its hydrophobicity was computed by assigning Kyte–Doolittle hydropathy values to each residue and averaging across the region. As shown in New Table S3, PD-L1 ranks among the least hydrophobic SPs in the human proteome, confirming its unusually suboptimal nature and quantitatively extending our previous observations.

To address whether other SPs might share properties enabling delayed Sec61 engagement, we applied additional selection criteria known to influence translocation efficiency:

- low h-region hydrophobicity (similar to PD-L1),

- presence of aromatic residues (F/Y/W),

- absence or presence of leucine stretches, which strongly promote co-translational Sec61 engagement (White S. and von Hejine G., Annu Rev Biophys, 2008).

Based on these parameters, we selected some SPs predicted and experimentally tested them using the cytoRUSH assay, as suggested. This analysis successfully identified SPs with suggested delayed translocation properties (CD44, a-galactosidase A (AGAL) Sun *et al.*, J Cell Biol, 2023), which behaved similarly to SPn in cytoRUSH, exhibiting strong biotin-dependent rescue. Conversely, SPs enriched in leucines and with high hydrophobicity (e.g., IL2, CD8α) showed no biotin sensitivity, consistent with efficient co-translational translocation. SPs previously described to undergo post-translational insertion (prolactin (PRL) Lakkaraju *et al.*, Mol Biol Cell, 2012, pro-thyrotropin-releasing hormone (TRH) Lakkaraju *et al.*, Mol Biol Cell, 2012, statherin (STATH) Haßdenteufel S. *et al.*, FEBS Lett, 2019) exhibited robust biotin-dependent rescue, confirming the sensitivity of the assay. Finally, we tested the Programmed cell death 1 ligand 2 (PD1-L2), which shows a SP with low hydrophobicity comparable to PD-L1 but multiple leucine residues. Despite its hydrophobic weakness, PD-1L2 behaved like

SPopt, validating the predictive power of our selection criteria and highlighting how specific sequence features, not hydrophobicity alone, govern Sec61 engagement.

We have incorporated these analyses and experimental validations into the revised manuscript (new Fig. S4 and Table S3), and we believe they significantly strengthen the mechanistic relevance and general interest of our work.

2. I am not convinced of the current analysis of the PD-L1 SP hydrophobicity. Please explain how the h-region of each human SP was identified and how was the Kyte-Doolittle score calculated for each SP with differing lengths. For example, were SPs aligned based on the predicted SP cleavage site? I would like to see a more detailed analysis of h-region hydrophobicity of PD-L1 compared to all other human SPs. How different is PD-L1 in hydrophobicity or does it have some other biophysical properties that distinguish it from other SPs? What supports the notion that PD-L1 has a suboptimal SP?

We thank the reviewer for the insightful comment. In continuation to our answer to the previous point and to clarify our methodology, we extracted all human SPs from the Signal Peptide Database (http://www.signalpeptide.de/?m=searchspdb). The h-region of each SP was then identified using SignalP 6.0 (https://services.healthtech.dtu.dk/services/SignalP-6.0/) with the following parameters: Organism: Eukarya; Output format: Long output; Model mode: Slow. After defining the h-region for each SP, we calculated its hydrophobicity using the Kyte–Doolittle hydropathy scale, which assigns numerical hydropathy values to individual amino acids and averages them across the selected region. All SPs were then ranked from the least to the most hydrophobic based on their h-region Kyte–Doolittle values (Kyte J. and Doolittle R. F., J Mol Biol, 1982). This allowed us to identify SPs with comparatively low hydrophobicity, which we refer to as suboptimal.

Within this distribution, the PD-L1 SP ranks among the least hydrophobic human SPs (Kyte–Doolittle values ranging from 2.6 to 60.7, with PD-L1 positioned at 643 out of 3,125 SPs analyzed). In addition to its low hydrophobicity, we also considered the presence of particular amino-acid features (including aromatic residues) that could further distinguish suboptimal SPs. These combined characteristics support the notion that the PD-L1 SP exhibits suboptimal biophysical properties compared to typical SPs (new Table S3).

3. In the Discussion the authors discuss competition between prokaryotic SRP and Trigger factor and possible competitive processes with SRP in eukaryotes. Here, it would be useful to

discuss the mechanism of competitive protein targeting by NAC and SRP that Ban and Deuerling groups have recently characterized.

We thank the reviewer for this insightful suggestion. We have now incorporated a discussion of the recently characterized competitive targeting mechanism between NAC and SRP into the revised Discussion.

Minor points:

1. Page 2. The sentence "Recently, it has been shown that some proteins with a SP can be fully synthetized in the cytosol and translocated into the ER only at the end of the synthesis in a post-translational manner.". This has in fact been known for quite some time, I would remove "Recently".

As suggested, we removed the word "Recently" from the sentence to avoid implying novelty where the mechanism is already well established.

2. Page 3. "Despite lacking a signal sequence for ER translocation, luminal localization has been proposed for these kinases.". I am not aware of such publications, please provide references or remove this sentence.

We thank the reviewer for pointing this out. We have now added the appropriate references supporting the proposed luminal localization of these kinases.

3. Page 4. "Previous studies have shown that PD-L1 luminal domain is modified by several cytosolic kinases.". Please include references for this statement.

We have added the relevant references supporting the involvement of cytosolic kinases in the modification of the PD-L1 luminal domain.

4. Page 5. The authors cite metformin as a well-characterized AMPK activator, but no reference is given. It would be ideal if the authors could demonstrate activation of AMPK following their treatment by for examples demonstrating a downstream AMPK substrate phosphorylation.

We thank the reviewer for this helpful comment. We have now added an appropriate reference supporting the use of metformin as a well-characterized AMPK activator (Zhou G. *et al.*, J Clin

Invest, 2001). In addition, our data already provide two lines of evidence for AMPK activation upon metformin treatment, both shown in Figure 2D: (i) the phosphorylation of AMPKα at Thr172 (Zhou G. *et al.*, J Clin Invest, 2001), and (ii) the increased phosphorylation of PD-L1 driven by the SPn construct, which is consistent with AMPK pathway activation. These observations together confirm that metformin effectively activates AMPK under our experimental conditions.

5. The authors state the PD-L1 SP may be inefficient in membrane translocation, but it is also quite likely inefficient in engaging with SRP and in subsequent ER targeting of the RNC.

We thank the reviewer for this insightful comment. As suggested, we have now included in the revised Discussion a dedicated paragraph addressing the possibility that the PD-L1 SP may also inefficiently engage SRP and thereby affect ER targeting of the RNC. This new section discusses how suboptimal or weak SPs may delay SRP recruitment and transiently expose the nascent chain to the cytosol, integrating recent mechanistic insights into competitive interactions between SRP, NAC and other ribosome-associated factors.

6. Page 6. aTubulin should be spelled a-tubulin (with alpha instead of a of course).

We thank the reviewer for the observation. The spelling of α-tubulin has been corrected accordingly.

7. It is interesting that the slower translation rate prevents exposure of mature part of PD-L1 to the cytosol. Do the authors assume that this is reflecting stalled ER translocation by the native PD-L1 SP? In other words, the native PD-L1 SP would remain bound at the Sec61 translocon or would have low propensity to insert through the Sec61 pore and the lateral gate.

We thank the reviewer for this insightful mechanistic question. Based on our data, we indeed favour a model in which the native PD-L1 SP has a reduced propensity to efficiently engage and/or insert into the Sec61 translocon, leading to delayed ER translocation. In this scenario, a fraction of the nascent chains would transiently fail to insert into the Sec61 translocon, allowing exposure of the mature PD-L1 region to the cytosol when translation is fast. When the translation rate is reduced, the longer time window likely favours productive engagement of the native SP with the Sec61 complex before the mature domain emerges from the ribosome, thereby preventing its cytosolic exposure. However, our experiments do not allow us to

distinguish whether the native SP remains stably bound at Sec61 in a stalled intermediate or whether it displays repeated, low-efficiency insertion events. We have now clarified this interpretation and its limitations in the revised manuscript.

8. Page 9. The authors state: “it was proposed that a fraction of these different cytosolic kinases is translocated into the lumen of the ER, despite the absence of a SP”. Please provide a reference for this statement.

We thank the reviewer for this comment. We have added the relevant reference supporting the proposed translocation of cytosolic kinases into the ER lumen.

9. Page 10. “pooper insertion of the SP” should read “proper insertion”.

We thank the reviewer for noticing this typo. The error has been corrected to “proper insertion”.

REVIEWER #3 (REMARKS TO THE AUTHOR):

In the manuscript “A delayed translocation into the endoplasmic reticulum controls the post translational modifications of PD-L1” Cannata Serio et al. describe a how sub-optimal signal-peptide sequences can delay translocation into the ER and open up the opportunity for enzymes to modify the nascent protein in the cytoplasm before being translocated into the ER. The authors use PD-L1 as example and show that an optimized SP, delayed protein translation by cycloheximide or introduction of a large linker, abolish AMPK-dependent phosphorylation of a serine 195 in PD-L1. Moreover, the authors show that protein transport of PD-L1 from the ER is perturbed if the SP is exchanged to an optimized SP, indicating viable post-translational modifications are missing.

The manuscript addresses a mechanism for regulation of expression of transmembrane and secretory proteins that is largely unknown and demonstrates well how different alterations impact the AMPK-dependent phosphorylation of PD-L1. The authors provide mechanistical insights on how cytosolic kinases would be able to add PTMs to proteins that are directly translated into the ER, a mechanism that has not been described before. The authors make clever use of novel assays such as cytoRUSH which was developed for this study, and methods such as TurboID-based biotinylation which was repurposed in this study to clarify the role of SPs in protein modification. The manuscript itself is well structured and I could easily follow the authors train of thought.

Overall, I think quality of the study and the shown technical expertise warrant a publication in Nature Communications. However, the story misses in my opinion two things that should be addressed before I support its publication without reservations:

A) I think it is very exciting that one can not only see that optimization of SP seems to prevent AMPK—mediated degradation of the PD-L1, a fact that is shown by the authors in very convincing fashion, but on the other hand also defects its trafficking from the ER. In its current state the manuscript only briefly covers the observed phenotype but lacks further information and speculation, although I believe addition information could improve the strength of the manuscript. The authors do report that the trafficking is affected but do not speculate about why the trafficking is affected. Is it possible that this is due to another PTM that cannot occur?

We thank the reviewer for the very positive assessment and for this insightful suggestion. We agree that the trafficking defect of the SPopt–PD-L1 variant deserves further discussion. Our data show that SP optimization not only prevents AMPK-dependent degradation, but also leads to impaired glycosylation, defective maturation and reduced surface expression, consistent with altered ER export and/or quality control as well as reduced Golgi-dependent transport.

In the revised Discussion, we now speculate that this phenotype may indeed reflect the loss of additional regulatory events that normally occur during delayed translocation. By abolishing the time window in which the luminal domain of PD-L1 is transiently exposed to the cytosol, SPopt may prevent the acquisition of other pre-translocational PTMs or protein–protein interactions that are required for proper folding, glycan processing and forward trafficking. While our current data do not allow us to pinpoint a specific missing modification beyond S195 phosphorylation, we have now explicitly highlighted this possibility as an attractive hypothesis for future investigation.

Here is my suggestion how this could be addressed:

1) The authors could clarify which step of the secretion pathway is affected: Therefore, the analysis and interpretation of the microscopy data could be improved. Importantly, it needs to be noted, that the SPopt-PDL1 seems to accumulate in punctuated structures that are not positive for calnexin but probably positive for KDEL (although the cell that was picked here, shows KDEL signal nearly everywhere, compared to the cell that was picked for the SPn fusion protein).

Furthermore, I would not say that the SPopt-PDL1 shows increased colocalization with the Golgi-marker GM130. From the microscopy image you can clearly see that they barely overlap (also the Pearson suggests that). The fact that the signal is increased, is rather caused by the protein being stuck somewhere along the early secretory pathway.

The authors could perform experiments with an ERGIC (ERGIC53 for example) and ER-exit site marker (Sec16 for example), to pin point which step of the secretion pathway is affected. The performed experiment to test the glycosylation status (Fig. 4 G-H) also strongly suggest that ER export is affected by the optimized SP. In addition, I would advise to use the Manders-coefficient for analysis instead of the Pearson. Specifically, when comparing with KDEL one can see that both proteins seem to perfectly colocalize when the SPopt-variant is used, indicating that it overlaps with KDEL positive structures (likely ER-exit sites).

We thank the reviewer for these very valuable comments and for highlighting the need to more precisely define which step of transport along the secretory pathway is affected by SP optimisation. To address this point, we generated stable HeLa cell lines expressing either ^{SPn}- or ^{SPopt}PD-L1-HA, allowing us to evaluate trafficking defects under steady-state conditions and to avoid artefacts associated with transient overexpression. Importantly, the results obtained in these stable lines fully recapitulated what we observed in GB138 stable cell line, further supporting the robustness of our conclusions.

As recommended, we expanded our analysis to include markers of additional secretory pathway stations. Specifically, we examined co-localisation with TANGO1 (ER exit sites, ERES) and ERGIC-53 (ER-Golgi intermediate compartment, ERGIC). ^{SPn}PD-L1-HA showed minimal overlap with either marker and was predominantly found at the plasma membrane, consistent with efficient ER export and normal trafficking. In contrast, ^{SPopt}PD-L1-HA exhibited strong co-localisation with both TANGO1 and ERGIC-53, indicating that the optimised SP causes PD-L1 to accumulate at very early secretory pathway stations, either at or immediately after ER exit. These data extend and refine the interpretation of our original microscopy, which already indicated increased co-localisation with KDELr and a pronounced retention in ER-associated structures. As correctly noted by the reviewer, ^{SPopt}PD-L1 does not appreciably accumulate in the cis-Golgi (GM130), despite its stronger signal in the perinuclear region. Consistent with this, Manders' coefficient shows only minimal overlap of ^{SPopt}PD-L1 with GM130 compared to ER-resident markers, suggesting that only a small fraction reaches the Golgi (new Supplemental Figure S10).

We also followed the reviewer's suggestion regarding the quantification method: given that PD-L1 distributes across heterogeneous membranous structures, we now use Manders' coefficients instead of Pearson's correlation, as they more accurately capture the fraction of PD-L1 residing in each compartment.

Together, these additional experiments demonstrate that SP optimisation primarily impairs ER export and causes PD-L1 to accumulate at ER, ERES and within the ERGIC, without substantially reaching downstream Golgi stations. These findings confirm and refine the conclusion that the native suboptimal SP plays a critical physiological role in ensuring proper PD-L1 trafficking through the early secretory pathway.

2) it would be great to propose a reason why the protein is not exported properly. Is it because it is lacking a specific PTM (probably a phosphorylation?). It would be great if the authors could speculate why the protein is stuck in the early secretory pathway to round up the experimental finding.

This is indeed an key point. Based on our data, we believe that the impaired export of ^{SPopt}PD-L1 is not primarily due to the absence of a specific PTM but rather reflects a broader consequence of forcing PD-L1 into a strictly co-translational translocation pathway.

As we extensively demonstrated, SP optimisation completely prevents AMPK-dependent phosphorylation of S195 by eliminating the transient cytosolic exposure of the luminal domain. However, we cannot predict whether this forced co-translational translocation may also alter additional phosphorylation events or other early modifications whose physiological relevance remains unknown. What is clear from our data is that forcing the co-translational translocation profoundly affects the early maturation of PD-L1. Indeed, the SPopt variant shows marked alterations in glycosylation and an increased propensity to form high-molecular-weight oligomers (new Fig. 4I), strongly suggesting folding defects. These defects occur well before Golgi entry, as shown by the complete EndoH and PNGaseF sensitivity of ^{SPopt}PD-L1. Such compromised folding is consistent with the idea that efficient SPs bypass the BiP-dependent translocation route, which is normally required for proteins with marginally hydrophobic SPs such as PD-L1 to achieve proper folding.

We therefore propose that the inability of ^{SPopt}PD-L1 to exit the ER is not due to the lack of a specific PTM per se, but rather to defective early maturation caused by an excessively rapid engagement with the Sec61 translocon. This results in a PD-L1 species that enters the ER

prematurely, without adequate opportunities for early folding or chaperone engagement, leading to retention at ER, ERES and ERGIC stations. Even when a fraction of the protein manages to exit the ER, it is likely retrieved back via the KDELr-mediated quality-control pathway, as indicated by its significant co-localisation with KDELr.

In summary, while S195 phosphorylation is clearly abolished by SP optimisation, we believe that the primary cause of ER retention is the disruption of the delicate balance between translocation kinetics and folding machinery engagement, which prevents ^{SPopt}PD-L1 from achieving the conformational state required for efficient ER export.

B) To round up the discussion it would be good to know whether it is a common feature of secretory proteins with a suboptimal SPs to have multiple sites for PTMs in their N-terminal region after the SP? Are the authors aware of other proteins that possess suboptimal SPs? And would these proteins also rely on slower translocation so that specific PTMs can be made?

We thank the reviewer for the very intriguing suggestion. We agree that a delayed translocation could provide an additional level of expression control through pre-translocational PTMs, but we think that proteins may experience delayed translocation kinetics, also to follow a proper folding pathway. The ER resident chaperone BiP (binding immunoglobulin protein) has been described as assisting the folding of post-translationally translocated proteins, as its primary role is to ensure translocon sealing and to provide the necessary force to allow the nascent chain to pass through the channel and enter the ER lumen (Matlack K. E. *et al.*, Cell, 1999). More recently, it has been shown that marginally hydrophobic signal sequences and transmembrane domains cause transient retention at the Sec61 translocon, and the luminal BiP is required for efficient protein translocation. In contrast, more efficient signal sequences bypass the BiP-dependent translocation, but translocated proteins are prone to misfold and aggregate in the ER under limited BiP availability (Sun *et al.*, J Cell Biol, 2023). PD-L1 needs BiP for achieving its proper folding (Chou CW. *et al.*, Am J Cancer Res, 2020), and we think the “weak” nature of the PD-L1 SP might has been selected for following this slower translocation pathway, which only in the second time might has allowed the selection of the additional expression control by pre-translocational PTMs.

Minor points:

1) the authors should consider showing the differences in amino acids of SPn and SPopt as part of Fig. 1, to make it easier to follow.

We have now included the SPn and SPopt amino-acid sequences in Figure 1B to make the comparison clearer.

2) Fig.1B: The images suggest, the EGFP-PD-L1 expression is higher in SPn cells after biotin addition compared the cells that have the SPopt and thus constantly express the EGFP-PD-L1, however the authors show in FigS2A+B that this not the case, similar to what I would expect. If this is simply caused by different contrast settings for the two sets of images, I would suggest to adjust the contrast for the lower panel to avoid confusion.

We thank the reviewer for this helpful observation. The apparent difference in EGFP–PD-L1 signal indeed resulted from automatically adjusted contrast and brightness settings applied to the first frame of each acquisition. We have now reprocessed the images using identical, non-automatic settings for both panels to avoid any misleading differences in signal intensity.

3) Fig.1E,F: I don't understand "relative" intensity of EGFP, relative to what? I cannot find this information in the methods (although I might have overlooked it). Also Fig.1F says (extra) instead of surface, is this something different or was overlooked when harmonizing the manuscript?

We thank the reviewer for bringing up this important point. In Figure 1E and 1F, the term "relative" intensity referred to the ratio between surface EGFP–PD-L1 and total cellular EGFP–PD-L1. We have now clarified this explicitly in the Methods section.

We also thank the reviewer for noticing the inconsistency in Figure 1F: the label "extra" was unintentionally introduced during figure harmonization. The figure has now been corrected to consistently use the term "surface".

4) Fig 2H: I assume it is not an anti-streptavidin-antibody but rather HRP-tagged streptavidin? Why both high expression EGFP and low expression EGFP are shown, when only under high EGFP expression biotinylation can be observed? I suspect two different enhancers are used? Also, it is not discussed in the manuscript why two different expression levels are shown.

We apologise for the mistake and have corrected it in the new version of the manuscript, replacing anti-streptavidin antibody with HRP-conjugated streptavidin.

Moreover, “Exp” in both higher and lower exp refers to exposure instead of expression. We chose to show either higher or lower exposure since the biotinylated-EGFP protein was visible only in higher-exposure view but, in order to compare the total protein expression levels of both SPn- or SPopt-EGFP, we showed the lower exp view since the higher one is totally saturated and does not allow comparison between adjacent bands.

REVIEWER #4 (REMARKS TO THE AUTHOR):

The authors show an interesting and important new regulatory mechanism for PD-L1 expression and modification. They show that the sub-optimal hydrophobicity of the H region of the signal peptide causes “delayed translocation” allowing a temporary exposure of the nascent chain to the cytosolic environment, enabling modifications of exoplasmic domains via cytosolic enzymes. They show optimizing the hydrophobicity of the signal peptide of PD-L1 affects the cytosolic exposure, disrupting PD-L1's trafficking, maturation, and localization. The results are novel and of interest to the large number of investigators interested in immune checkpoints.

1. “This pause allows several amino acids to be synthesized, exposing the nascent chain to the cytosolic environment.” Make some comment on how many amino acids “several” is. Ser195 modification is affected which is more than several.

We appreciate the reviewer’s observation. In the original text, “several amino acids” was meant as a qualitative description of the region of the nascent chain that becomes exposed during the pause. We agree that this phrasing is too vague, especially considering that the modification at Ser195 involves a segment longer than just “several” residues.

To clarify this point, we now specify in the revised manuscript that the pause results in the exposure of approximately 200 amino acids of the nascent chain, sufficient to include Ser195. This correction removes the ambiguity and more accurately reflects the structural context of the modification.

2. Write out full name of SBP, describe. The SBP-tag sequence is 38 amino acids long (or

whatever used) and binds to streptavidin with an equilibrium dissociation constant of 2.5 nM. Reference Barrette PMCID: PMC3640474. Also say whether it is placed before or after the predicted signal cleavage site.

In the revised manuscript, we now spell out the full name of SBP (Streptavidin-Binding Peptide), and also added the requested reference (Barrette-Ng I. H. *et al.*, Acta Crystallogr, 2013). Moreover, we now clearly indicate the position of the SBP-tag relative to the predicted SP cleavage site.

3. In Fig 1B, indicate time scale hr:min or min:sec?

We thank the reviewer for the comment. We now indicate that the time scale in Fig. 1B is expressed in "hr:min", both in the figure legend and in the corresponding text.

4. Fig 1C-F needs a control of no biotin for each of the time points.

We thank the reviewer for this helpful suggestion. In our experimental setup, we typically do not include a no-biotin control for each individual time point. Instead, we use the "overnight biotin" and "overnight no-biotin" conditions as global controls for the entire experiment, which allow us to assess background signal and the overall dynamic range of the assay.

5. Typo: through PTMs 18 (Cha, 2018 #13)

We thank the reviewer for pointing out this typo. The reference has been corrected accordingly.

6. In 1D, why are there 2 probings for vinculin? If this is two blots, separate into 2 groupings.

We thank the reviewer for the comment. The two vinculin probings in Figure 1D correspond to two separate input blots. In the revised version of the figure, we have now made this distinction explicit and visually clearer by separating the blots into two groupings.

7. Page 6. "SPopt would alleviate this lagging time". Explain more clearly in context of preceding sentence. Explain better why "slower translation rate, would prevent exposure of S195 in the cytosol ".

We thank the reviewer for this helpful comment. We have clarified the text accordingly. SPopt, being more hydrophobic and efficiently recognized by SRP, is expected to engage the Sec61 translocon more rapidly, thereby eliminating the lag time that we observe with the native suboptimal SPn. This reduces the window during which the emerging luminal region of PD-L1 can be exposed to the cytosol.

We also explain more clearly why a slower translation rate prevents exposure of S195 to the cytosol. When elongation is slowed down, the suboptimal SPn has more time to productively engage the Sec61 translocon before the S195-containing region emerges from the ribosome. As a consequence, the nascent chain is efficiently directed into the ER, preventing transient cytosolic exposure of S195. This interpretation is consistent with our experimental observations showing that low doses of CHX alleviate metformin-sensitive phosphorylation of S195.

These clarifications have now been incorporated into the revised manuscript.

8. “the SPn-PD-L1-HA and the SPopt-PD-L1-HA sequences were generated from synthetic genes”. Specify whether these are native sequences of PD-L1 or codon-optimized. If codon-optimized, discuss how this might affect results.

We thank the reviewer for the comment. The SPn and the PD-L1 sequences are based on native sequences, extracted from NCBI. This information has now been clarified in the Materials and Methods section.

9. In sup fig 1A, C and others showing sequence, use a monospaced font like Courier. We thank the reviewer for the suggestion. We have updated Supplemental Figure 1A and Supplemental Figure 1C (now Figure 1B), and all other sequence-containing figures to use a monospaced font.

10. Move Sup Fig 1C to main Fig 1.

We thank the reviewer for the helpful suggestion. As requested, Supplemental Figure 1C has been moved to the main Figure 1B.

11. Supp Table S2. Currently calculated for 100 random SP. Replace with a Table S2A with 100 random cell surface SP and Table S2B with 100 random secreted SP.

We thank the reviewer for the suggestion. As requested, we have revised Supplemental Table S2 by separating it into Table S2A, containing 100 random cell-surface SPs, and Table S2B, containing 100 random secreted SPs.

12. Somewhere in Suppl, indicate treatments shown in each of 6 supplemental videos.

We thank the reviewer for the comment. A description of the treatments shown in each of the six supplemental videos is provided on pages 36-37 of the Supplemental Materials.

13. If they wish, the authors could comment in the discussion that use of native signal peptides in recombinant constructs will best reflect natural functions of the protein.

We thank the reviewer for this valuable suggestion. We fully agree that our findings highlight the importance of using native signal peptides when generating recombinant constructs, as optimized or heterologous SPs may fail to reproduce the physiological maturation, trafficking, and regulatory properties of the protein. We have now incorporated this point into the Discussion section of the revised manuscript.

REVIEWER #5 (REMARKS TO THE AUTHOR):

I co-reviewed this manuscript with one of the reviewers who provided the listed reports. This is part of the Nature Communications initiative to facilitate training in peer review and to provide appropriate recognition for Early Career Researchers who co-review manuscripts.

REVIEWER COMMENTS

Reviewer #1 (Remarks to the Author):

The authors have addressed most of the raised concerns.

Reviewer #2 (Remarks to the Author):

Review of manuscript of Serio et al. entitled: ‘A delayed translocation into the endoplasmic reticulum controls the post translational modifications of PD-L1’

The authors have carried out most of the analyses and experiments requested by the reviewers and in my view, this has significantly strengthened the conclusions of the manuscript. The finding that both an optimized SP sequence and rate of elongation are both important for production of mature PD-L1 suggests complex requirement for optimization of co-translational ER insertion kinetics. While many mechanistic questions regarding the delayed PD-L1 ER translocation process remain open for future studies, this is an important work highlighting a specialized function of a seemingly inefficient signal peptide with implication for optimized protein folding and quality control with possible relevance in recombinant protein production.

I only have one minor remaining questions for the authors before the article is ready for publication.

1. On page 5. the authors state that preprolactin signal peptide (as well as TRH and STATH) would render the tested PD-L1 construct to be targeted to the ER post-translationally and this would explain the biotin-dependent rescue of cell surface expression. Preprolactin is a well-known efficiently targeting signal peptide and I do not find a reason why it would render the rather long PD-L1 construct to be targeted post-translationally. Without further evidence showing that the tested PD-L1 construct with a preprolactin SP its targeted post-translationally, I would not include this notion in the main text.

We thank the reviewer for this comment. We agree with the reviewer’s assessment and have removed this statement from the main text.

2. This figure (Fig. S6) would benefit from showing the SP sequences that were tested and including their calculated h-region hydrophobicity values. At the moment, it is not clear from the figure which SPs are presumed to be efficiently targeted based on their predicted biophysical properties.

We thank the reviewer for this comment. We have revised Fig. S2 and S4 to include the tested signal peptide sequences and their calculated h-region hydrophobicity values, clarifying which SPs are predicted to be efficiently targeted.

Reviewer #3 (Remarks to the Author):

I am happy with the authors revisions and answers and support publication.

Reviewer #4 (Remarks to the Author):

The authors have done additional experimental work, clarified their explanations, and addressed my concerns. I have a few minor suggestions.

1. Usual abbreviated name is PD-L2, not PD1-L2.

We have corrected the abbreviation throughout the manuscript, replacing "PD1-L2" with the commonly used and accepted term "PD-L2".

2. "our findings emphasize that the use of native, non-optimized SPs in recombinant constructs is essential to faithfully reproduce the natural maturation, trafficking and regulatory properties of the corresponding proteins. "This is too definitive a claim, re-phrase more cautiously such as: "our findings emphasize that the use of native, non-optimized SPs in recombinant constructs is important to faithfully reproduce the natural maturation, trafficking and regulatory properties of the corresponding proteins and that the use of optimized SP in recombinantly produced proteins may lead to the loss of some of these features".

We thank the reviewer for this valuable suggestion. We have revised the text to adopt a more cautious wording.

3. Highlight PD-L1 signal sequence in Tables.

We thank the reviewer for this suggestion. We have highlighted the PD-L1 signal sequence in Tables S1 and S3.

4. On page 4 of Supp, “Both SPn and PD-L1 correspond to the native sequences extracted from NCBI.” Say “native DNA sequence” so there is no ambiguity with protein sequence.

We thank the reviewer and have revised the text accordingly.

Reviewer #5 (Remarks to the Author):

I co-reviewed this manuscript with one of the reviewers who provided the listed reports. This is part of the Nature Communications initiative to facilitate training in peer review and to provide appropriate recognition for Early Career Researchers who co-review manuscripts.
